# Machine learning for classification of hypertension subtypes using multi-omics: A multi-centre, retrospective, data-driven study

**DOI:** 10.1016/j.ebiom.2022.104276

**Published:** 2022-09-27

**Authors:** Parminder S. Reel, Smarti Reel, Josie C. van Kralingen, Katharina Langton, Katharina Lang, Zoran Erlic, Casper K. Larsen, Laurence Amar, Christina Pamporaki, Paolo Mulatero, Anne Blanchard, Marek Kabat, Stacy Robertson, Scott M. MacKenzie, Angela E. Taylor, Mirko Peitzsch, Filippo Ceccato, Carla Scaroni, Martin Reincke, Matthias Kroiss, Michael C. Dennedy, Alessio Pecori, Silvia Monticone, Jaap Deinum, Gian Paolo Rossi, Livia Lenzini, John D. McClure, Thomas Nind, Alexandra Riddell, Anthony Stell, Christian Cole, Isabella Sudano, Cornelia Prehn, Jerzy Adamski, Anne-Paule Gimenez-Roqueplo, Guillaume Assié, Wiebke Arlt, Felix Beuschlein, Graeme Eisenhofer, Eleanor Davies, Maria-Christina Zennaro, Emily Jefferson

**Affiliations:** a Division of Population Health and Genomics, School of Medicine, University of Dundee, Dundee DD2 4BF, UK; b School of Cardiovascular and Metabolic Health, BHF Glasgow Cardiovascular Research Centre, University of Glasgow, Glasgow G12 8TA, UK; c Institute of Clinical Chemistry and Laboratory Medicine, University Hospital Carl Gustav Carus, TU Dresden, Dresden, Germany; d Institute of Metabolism and Systems Research, College of Medical and Dental Sciences, University of Birmingham, Birmingham, UK; e Centre for Endocrinology, Diabetes and Metabolism, Birmingham Health Partners, Birmingham, UK; f Department of Endocrinology, Queen Elizabeth Hospital Birmingham, University Hospitals Birmingham NHS Foundation Trust, Birmingham, UK; g Klinik für Endokrinologie, Diabetologie und Klinische Ernährung, UniversitätsSpital Zürich (USZ) und Universität Zürich (UZH), Zurich, Switzerland; h Université Paris Cité, PARCC, INSERM, F-75006 Paris, France; i Assistance Publique-Hôpitaux de Paris, Hôpital Européen Georges Pompidou, Unité Hypertension artérielle, Paris, France; j Department of Medicine III, University Hospital Carl Gustav Carus, TU Dresden, Dresden, Germany; k Division of Internal Medicine and Hypertension Unit, Department of Medical Sciences, University of Torino, Italy; l Assistance Publique-Hôpitaux de Paris, Hôpital Européen Georges Pompidou, Centre d'Investigations Cliniques 9201 Paris, France; m Department of Hypertension, National Institute of Cardiology, Warsaw, Poland; n UOC Endocrinologia, Dipartimento di Medicina DIMED, Azienda Ospedaliera-Università di Padova, Padua, Italy; o Medizinische Klinik und Poliklinik IV, Klinikum der Universität München, LMU München, Munich, Germany; p Clinical Chemistry and Laboratory Medicine, Core Unit Clinical Mass Spectrometry, Universitätsklinikum Würzburg, Germany; q Schwerpunkt Endokrinologie/Diabetologie, Medizinische Klinik und Poliklinik I, Universitätsklinikum Würzburg, Germany; r Comprehensive Cancer Center Mainfranken, Universität Würzburg, Würzburg, Germany; s The Discipline of Pharmacology and Therapeutics, School of Medicine, National University of Ireland 33 Galway, Ireland; t Department of Medicine, Section of Vascular Medicine, Radboud University Medical Center, Nijmegen, the Netherlands; u Internal & Emergency Medicine- ESH Specialized Hypertension Center, Department of Medicine-DIMED, University of Padua, Padua, Italy; v Department of Computing and Information Systems, University of Melbourne, Melbourne, Australia; w University Hospital Zurich University Heart Center, Cardiology, and University of Zurich, Zurich, Switzerland; x Metabolomics and Proteomics Core (MPC), Helmholtz Zentrum München, German Research Center for Environmental Health, Neuherberg, Germany; y Institute of Experimental Genetics, Helmholtz Zentrum München, German Research Center for Environmental Health, Ingolstädter Landstraße 1, 85764 Neuherberg, Germany; z Department of Biochemistry, Yong Loo Lin School of Medicine, National University of Singapore, 8 Medical Drive, Singapore 117597, Singapore; aa Institute of Biochemistry, Faculty of Medicine, University of Ljubljana, Vrazov trg 2, 1000 Ljubljana, Slovenia; ab Service de Génétique, Assistance Publique-Hôpitaux de Paris, Hôpital Européen Georges Pompidou, F-75015 Paris, France; ac Université de Paris, Institut Cochin, INSERM, CNRS, F-75014 Paris, France; ad Department of Endocrinology, Center for Rare Adrenal Diseases, Assistance Publique–Hôpitaux de Paris, Hôpital Cochin, 75014 Paris, France; ae NIHR Birmingham Biomedical Research Centre, University Hospitals Birmingham NHS Foundation Trust and University of Birmingham, Birmingham, UK; af Institute of Health & Wellbeing, University of Glasgow, Glasgow G12 8RZ, UK

**Keywords:** Machine learning, Multi-omics, Hypertension, Primary aldosteronism, Pheochromocytoma/paraganglioma, Cushing syndrome, Biomarkers

## Abstract

**Background:**

Arterial hypertension is a major cardiovascular risk factor. Identification of secondary hypertension in its various forms is key to preventing and targeting treatment of cardiovascular complications. Simplified diagnostic tests are urgently required to distinguish primary and secondary hypertension to address the current underdiagnosis of the latter.

**Methods:**

This study uses Machine Learning (ML) to classify subtypes of endocrine hypertension (EHT) in a large cohort of hypertensive patients using multidimensional omics analysis of plasma and urine samples. We measured 409 multi-omics (MOmics) features including plasma miRNAs (PmiRNA: 173), plasma catechol O-methylated metabolites (PMetas: 4), plasma steroids (PSteroids: 16), urinary steroid metabolites (USteroids: 27), and plasma small metabolites (PSmallMB: 189) in primary hypertension (PHT) patients, EHT patients with either primary aldosteronism (PA), pheochromocytoma/functional paraganglioma (PPGL) or Cushing syndrome (CS) and normotensive volunteers (NV). Biomarker discovery involved selection of disease combination, outlier handling, feature reduction, 8 ML classifiers, class balancing and consideration of different age- and sex-based scenarios. Classifications were evaluated using balanced accuracy, sensitivity, specificity, AUC, F1, and Kappa score.

**Findings:**

Complete clinical and biological datasets were generated from 307 subjects (PA=113, PPGL=88, CS=41 and PHT=112). The random forest classifier provided ∼92% balanced accuracy (∼11% improvement on the best mono-omics classifier), with 96% specificity and 0.95 AUC to distinguish one of the four conditions in multi-class ALL-ALL comparisons (PPGL vs PA vs CS vs PHT) on an unseen test set, using 57 MOmics features. For discrimination of EHT (PA + PPGL + CS) vs PHT, the simple logistic classifier achieved 0.96 AUC with 90% sensitivity, and ∼86% specificity, using 37 MOmics features. One PmiRNA (hsa-miR-15a-5p) and two PSmallMB (C9 and PC ae C38:1) features were found to be most discriminating for all disease combinations. Overall, the MOmics-based classifiers were able to provide better classification performance in comparison to mono-omics classifiers.

**Interpretation:**

We have developed a ML pipeline to distinguish different EHT subtypes from PHT using multi-omics data. This innovative approach to stratification is an advancement towards the development of a diagnostic tool for EHT patients, significantly increasing testing throughput and accelerating administration of appropriate treatment.

**Funding:**

European Union's Horizon 2020 Research and Innovation Programme under Grant Agreement No. 633983, Clinical Research Priority Program of the University of Zurich for the CRPP HYRENE (to Z.E. and F.B.), and Deutsche Forschungsgemeinschaft (CRC/Transregio 205/1).

**Research in context**

**Evidence before this study**

We searched PubMed from database inception to Jan 18, 2022, using the search terms ((machine learning) OR (artificial intelligence)) AND ((primary aldosteronism) OR (pheochromocytoma) OR (paraganglioma) OR (Cushing's syndrome) OR (endocrine hypertension) OR (hypertension)) AND (Multi-omics), with no language restrictions. Our search yielded 10 results. Three articles reviewed the use of artificial intelligence in cardiovascular medicine and hepatology. Although one similar review article focussed on primary hypertension, however, it did not include any multi-omics based study on hypertension. Five research articles used machine learning in conjunction with multi-omics for major heart diseases, intraocular pressure and metabolic syndrome. One of the articles studied heart failure in rats. No articles using multi-omics data with machine learning for stratifying endocrine hypertension patients were found.

**Added value of this study**

To the best of our knowledge, this is the first-ever study to investigate machine learning-based classification of endocrine hypertension subtypes using multi-omics data. This large retrospective study included patients from 11 reference centres for endocrine hypertension with access to a complete set of biosamples, and multi-centre omics measurements. Our machine learning pipeline enabled the classification of 5 disease combinations using 8 supervised ML classifiers and to discover the best discriminating features. This approach outperformed the individual omics models when used for stratifying hypertensive subtypes in a multi-class scenario.

**Implications of all the available evidence**

Our multi-omics based machine learning approach to stratification is an advancement towards the development of an innovative tool in the diagnosis of hypertensive patients in clinical practice, greatly increasing throughput and accelerating the administration of appropriate treatment. It will also help identification of previously unsuspected molecules as biomarkers of complex, chronic diseases. The effect of this approach in clinical practice will be further tested in a prospective study and in a randomised controlled trial.

Alt-text: Unlabelled box

## Introduction

Arterial hypertension is one of the major chronic diseases leading to high morbidity and mortality worldwide.^1^ Globally, an estimated 25% of the world's adult population is affected by hypertension, which represents a tremendous public health burden.^2^ Hypertension carries an increased risk for various cardiovascular and renal diseases, such as myocardial infarction, stroke, heart failure, and chronic kidney disease.^3^^,^^4^

The majority of cases are classified as ‘primary’ (essential) hypertension (PHT). PHT is a multifactorial and complex disease with causes and contributing factors still incompletely understood. A combination of genetic and socio-environmental risk factors including salt intake, obesity, alcohol consumption, chronic stress, and urbanisation have been associated with PHT.^4^ In ∼10–15% of cases, a specific cause underlying hypertension can be identified. These cases of hypertension are classified as ‘secondary’ hypertension.^5^ Identification of secondary forms of hypertension is key for targeted management and reduction of cardiovascular complications. Among them, ‘endocrine’ hypertension (EHT), is caused by excess hormone production leading to increased blood pressure, such as primary aldosteronism (PA), pheochromocytoma/catecholamine-producing paraganglioma (PPGL), and endogenous cortisol excess i.e. Cushing's syndrome (CS).^6^ These cases currently account for ∼5–10% of total cases, a rate that reaches ∼25–30% at referral tertiary centres. Among them, PA represents the most common and curable form of secondary hypertension, with a prevalence of ∼6% in primary care and up to 10% in referred patients;^7^, ^8^, ^9^ the prevalence increases up to 20% in patients with resistant hypertension.^10^

Exclusion of secondary hypertension including EHT is necessary to diagnose PHT. This usually entails lengthy evaluation involving blood and urinary tests, imaging procedures and eventually invasive testing.^6^ In the past, this has provided the impetus to investigate and develop reliable and sensitive biomarkers for hypertension subtyping.^11^ Currently, EHT is frequently overlooked and its diagnosis is delayed by several years after the onset of hypertension, exposing patients to an increased risk of renal and cardiovascular damage^12^^,^^13^ and a diminished quality of life.^14^

Over the past two decades, there have been significant advances in various omics technologies, such as genomics, transcriptomics, proteomics, and metabolomics.^15^ The integration of these omics provides insight into the complex pathophysiology of any given disease.^16^ Such multi-omics (MOmics) integration has also been applied to different studies for improved prognostics and predictive accuracy of disease phenotypes.^17^^,^^18^

The primary purpose of this study was to train Machine Learning (ML) algorithms for diagnosing endocrine hypertension subtypes using MOmics data. It also aims to provide an understanding of discriminating features and their importance to different disease combinations.

## Methods

### Patient details

Retrospective plasma and 24h urine samples from patients with PA, PPGL, and CS were provided by 11 collaborating reference centres for endocrine hypertension that were participants of the ENS@T-HT Horizon2020 consortium.^19^ The diagnosis was made following current guidelines.^20^, ^21^, ^22^, ^23^ Patients with PHT and NV were recruited by various partners of the project. Plasma and 24 h urine samples were collected from 487 male and female participants, who suffered from one of four hypertension subtypes (PA = 113, PPGL = 88, CS = 41, PHT = 112), or were normotensive volunteers (NV =133). The plasma and urine samples were stored at −80° C before dispatch and analysis.

### Ethics

All study protocols under which patients were recruited were approved by the local ethics committee at CPP Ile de France II (2012-A00508-35), Comité de Protection des Personnes Ile de France 4 (2015/63NICB), Technische Universität Dresden Ethikkommission (189062010/EK7122010), Munich: Ethikkommission der LMU München (379-10), Zurich: Kantonale Ethikkommission des Kanton Zürich (2017-00771), University of Würzburg (88/11), University Hospital Galway (C.A. 1082), Comitato Etico per la Sperimentazione Clinica della Provincia di Padova (3998/AO/16), Comitato Etico Interaziendale AOU Città della Salute e della Scienza di Torino - AO Ordine Mauriziano di Torino- A.S.L. TO1 (Prot. 0000759 Pratica CS2/112), Medisch-ethische toetsings commissie Oost-Nederland (N157215.091.1.6), National Institute of Cardiology, Warsaw, Poland (1233/2010), West Ethics Committee, Western Infirmary, Glasgow (AHT/JR) and MVLS College Ethics Committee, University of Glasgow (29/4/16, 20/07/2016). All subjects provided written informed consent before participation in protocols.

### Multi-omics data

The biosamples were provided to 5 omics data-generating collaborators of the ENS@T-HT Horizon2020 project (Appendix Table 1). These omics included PmiRNA, PMetas, PSteroids, USteroids, and PSmallMB. The details are as follows:

### Plasma miRNA

Levels of 179 human miRNAs were measured in plasma after extraction of total RNA from 200 µL EDTA-plasma using the miRNeasy Mini kit (QIAGEN, Manchester, UK). 4 µL of undiluted RNA was then reverse-transcribed to cDNA in a 20 µL reaction volume using the Universal cDNA synthesis kit II (Exiqon, Vedbaek, Denmark). Selected plasma miRNAs were quantified using Serum/Plasma Focus microRNA PCR Panels (384-well, V4.M, Exiqon) according to their standard protocol, in combination with ExiLENT SYBR® Green master mix (Exiqon) and ROX solution (Thermo Fisher, Renfrew, UK) on a Quantstudio 12K Flex Real-time PCR System (Thermo Fisher). Raw data generated by the QuantStudio System were analysed using GenEx software (v.6, MultiD Analyses, Vedbaek, Denmark). Quality controls all along the procedure were performed using different spike-in RNAs and cDNAs. Data normalisation was performed using the five miRNAs most stably-expressed across the dataset, as identified by Normfinder software;^24^ of the remaining miRNAs, 1 was excluded on QC grounds, leaving 173 feature miRNAs for analysis. The detailed protocol, quality controls and quantification procedures are described in Appendix Note 1.

### Plasma catechol O-methylated metabolites

Plasma free metanephrines, normetanephrine and metanephrine, and 3-methoxytyramine, the O-methylated metabolites of norepinephrine, epinephrine and dopamine, respectively, were analysed by liquid chromatography tandem mass spectrometry (LC-MS/MS), as described elsewhere.^25^^,^^26^

### Plasma steroids

Sixteen plasma steroid hormone profiles (See Appendix Table 2) were analysed by LC-MS/MS as described elsewhere.^27^

### Urinary steroids

Twenty-seven urinary steroid metabolites derived from glucocorticoid, mineralocorticoid and androgen biosynthesis as well as core steroid precursors were measured using multi-steroid profiling by LC-MS/MS. A description of this methodology has been published previously.^28^^,^^29^

### Plasma small metabolites

The targeted metabolomics approach was based on LC-MS/MS measurements using the Absolute*IDQ*^TM^ p180 Kit (BIOCRATES Life Sciences AG, Innsbruck, Austria). The assay allows the simultaneous quantification of 188 metabolites out of 10 µL plasma. The assay procedures of the Absolute*IDQ*^TM^ p180 Kit, as well as the metabolite nomenclature, have been described in detail previously.^30^, ^31^, ^32^

### Generation of the MOmics dataset

All the omics data was catalogued in RDMP^33^ for systematic access. Although biosamples for 487 patients were included in the study, after quality controls omics measurements for all five omics were available for 408 (CS = 30, PA = 100, PPGL = 69, PHT = 108, and NV = 101) patients (Appendix Table 3). In total 409 MOmics features (PmiRNA: 173, PMetas: 4, PSteroids: 16, USteroids: 27, and PSmallMB: 189) along with age and sex were exported and used to conduct supervised ML experiments (Table 1). The names of features were prefixed with ‘O1_’, ‘O2_’, ‘O3_’, ‘O4_’, ‘O5_’, and ‘O6_’ for PmiRNA, PMetas, PSteroids, USteroids, and PSmallMB respectively. The complete list of all MOmics features and their distribution is included in the Appendix Table 2. The pairwise relationship amongst all features was evaluated by clustering Pearson correlation coefficients (Appendix Figure 1).

**Table 1:** Demographic characteristics for primary aldosteronism (PA), pheochromocytoma or paraganglioma (PPGL), Cushing's syndrome (CS), primary hypertension (PHT) patients and normotensive volunteers (NV). ^†^**The NV are included for information only and not used for any model development or testing.**

Disease	Total (408)	Sex		Age (Percentiles)		
		Male (198)	Female (210)	Median	25^th^	75^th^
Primary Aldosteronism (PA)	100	57 (28.8%)	43 (20.5%)	47.0	42.0	54.3
Pheochromocytoma or Paraganglioma (PPGL)	69	30 (15.2%)	39 (18.6%)	52.0	42.0	64.0
Cushing's Syndrome (CS)	30	3 (1.5%)	27 (12.9%)	49.5	44.3	58.5
Primary Hypertension (PHT)	108	47 (23.7%)	61 (29.0%)	55.5	42.8	65.0
Normotensive Volunteers (NV)^†^	101	61 (30.8%)	40 (19.0%)	27.0	23.0	34.0

### Biomarker discovery using supervised machine learning

**Figure 1:**
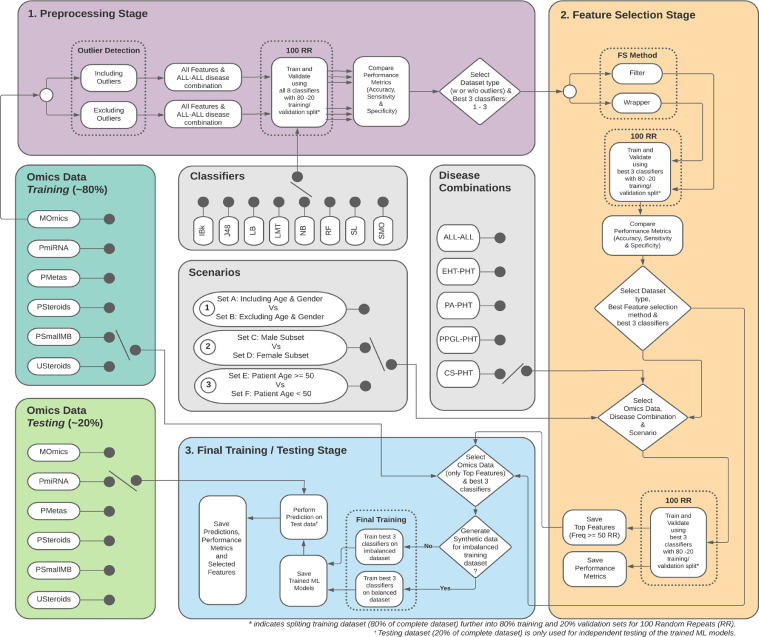
Schematic showing the details of the 3 stages ML-based pipeline.

The biomarker discovery involved the selection of disease combinations, outlier detection, choice of supervised ML classifiers, configuration of experiment parameters, and consideration of different evaluation scenarios (Figure 1).

### Training, validation and test dataset

The supervised machine learning used non-overlapping training, validation and test datasets. These can be defined as: (a) *training dataset*: The sample of data used to fit the model, (b) *validation dataset*: The sample of data used to validate the trained models during the 100 random repeats and (c) *test dataset*: The independent samples of ‘unseen’ data used to test the final trained model.

The MOmics and mono-omics datasets were once randomly split into training (∼80%) and testing (∼20%) set (Appendix Table 4). The training set was then further split into training-validation (80–20%) split for 100 Random Repeats (RR) using random seed for each iteration to ensure reproducibility. Finally, the test set was only used for independent validation of the final trained model and not used during any model training.

### Disease combinations

The five different disease combinations used for classification were: ALL-ALL (PPGL vs PA vs CS vs PHT), EHT (PPGL+PA+CS)-PHT, and each individual endocrine hypertension (i.e., PPGL/PA/CS)-PHT. These combinations did not include NV since the key question addressed was: How can hypertensive patients be stratified amongst themselves? Omics data from NV was used to compare individual biomarkers with patients of different hypertension types. This data was not used for any model development or testing.

### Outlier detection

To study the impact of outliers on classification, two sets of results were analysed, as shown in Stage 1 (Figure 1) i.e., 1) Using data including outliers and 2) applying 3 times 1.5 quartile method to remove extreme outliers (excluding outliers). The outliers were refilled using the maximum value.

### Classifiers, feature selection and classification performance metrics

An assorted set of 8 different classifiers: Decision Trees (J48),^34^ Naïve Bayes (NB),^35^ K-nearest neighbours (IBk),^36^ LogitBoost (LB),^37^ Logistic Model Tree (LMT),^38^ Simple Logistic (SL),^39^ Random Forest (RF),^40^ and Sequential Minimal Optimisation (SMO)^41^ were used (Stage 1 of Figure 1) with given hyperparameter settings (Appendix Table 5). The classification was implemented using the caret^42^ and RWeka^43^ in R^44^ and software code was released.^45^ For feature selection, wrapper (Boruta)^46^ and filter (Correlation-based feature selection – CFS)^47^ methods were compared (Stage 2 of Figure 1). The classifications were evaluated over 100 RR using performance metrics: balanced accuracy (to adjust for the class imbalance problem), sensitivity, specificity, AUC, F1, and Kappa score.

### Evaluation scenarios

One of the key objectives of the analysis was to identify the list of most discriminating features for a given disease combination. Possible bias due to the age or sex of the patients was studied with different sets of scenarios. Table 2 summarises the three scenarios, which were investigated for each disease combination along with their justification and Set combinations. Appendix Table 4 shows the corresponding patient count. The top features from Set A with a cut-off for the feature frequency of 50 was used for the final training/testing stage (Figure 1). This value was chosen empirically as a trade-off for finding the optimal number of reduced features without impacting the classification performance. Also, in order to minimise the impact of the class imbalance problem, additional synthetic samples were generated using the Synthetic Minority Over-sampling TEchnique ^48^ for CS and PPGL. In the case of the EHT-PHT disease combination, a down-sampling approach was used for class balancing instead of synthetic samples.

**Table 2:** Three scenarios using set A, B, C, D, E and F in pairs with their corresponding justifications.

Scenario Number	Scenario Details	Justification
1	**Set A** (using all omic features, age & sex) vs **Set B** (only using all omic features)	To study the impact of age and sex as discriminating features.
2	**Set C** (Male subset) vs **Set D** (Female subset)	To study the influence of sex by comparing the classification accuracy and to find sex-specific discriminating features.
3	**Set E** (Patient age >= 50) vs **Set F** (Patient age <50)	To investigate how omics are affected by age and hormonal status, based on average female menopausal age i.e. 50 years.

Stage 3 of the schematic shows the steps for the final/testing stage using original test data (Figure 1). The omic type and disease combination was selected, followed by the training of the best 3 classifiers. The trained model was then used to classify the test data. The prediction outcomes, the various performance metrics and the list of selected features were then saved and compared at the end of each classification. The characteristics of the final set of discriminating features were then evaluated with respect to NV using Principal Component Analysis. All the classifications shown in Figure 1 were employed on MOmics data and then on all five mono-omics individually.

### Role of the funding source

The funders of the study had no role in study design, data collection, data analysis, data interpretation, writing of the report and decision to submit the paper for publication.

## Results

### Patients and omics measurements

The study included 487 patients with PA, PPGL, CS and PHT as well as normotensive volunteers (NV) (PA = 113, PPGL = 88, CS = 41, PHT = 112, and NV =133), who were recruited by reference centres for adrenal disorders of the ENS@T-HT Horizon2020 consortium.^19^ Diagnosis was based on current guidelines for each disease in each expert centre. Omics studies involved measurements of plasma miRNA (PmiRNA: 173), plasma catechol O-methylated metabolites (PMetas: 4), plasma steroids (PSteroids: 16), urinary steroid metabolites (USteroids: 27), and plasma small metabolites (PSmallMB: 189) in biosamples collected from each patient within 24 h. After completion of omics measurements, quality controls and data cleaning, 408 patients had complete omics sets for further analysis (Appendix Table 3). Patients’ demographic characteristics are summarized in Table 1.

### Biomarker discovery using supervised machine learning

The biomarker discovery comprised three stages (Figure 1): a pre-processing (outlier detection and choice of supervised ML classifiers) in stage 1, feature selection in stage 2 and final training/testing in stage 3. Classification was performed on ALL-ALL (PPGL vs PA vs CS vs PHT), EHT (PPGL+PA+CS)-PHT, and each individual endocrine hypertension (i.e., PPGL/PA/CS)-PHT.

### Evaluation of data-driven pre-processing and feature selection methods

First, the classification performance for ALL-ALL using MOmics data excluding and including outliers was evaluated (Appendix Figure 2a). Excluding outliers provided better classification as observed from various performance metrics. SL provided ∼4%, 5%, and 1% increases in balanced accuracy, sensitivity, and specificity when excluding outliers, respectively. Overall, LB, SL, and RF were the best performing classifiers. Next, feature selection methods were compared using LB, RF, and SL classifiers for ALL-ALL disease combination (Appendix Figure 2b). Both filter and wrapper methods provided comparable classification performance. However, wrapper method was chosen for next stages of analysis since it evaluates the feature subsets as search problem to find key dependencies amongst features.

### Overall classification of primary and endocrine hypertension

The top 3 ML models were trained using the reduced training dataset which used top features from 100 RR for each disease combination (See stage 3 in Figure 1). These trained classifiers were then evaluated on the test set. The corresponding performance metrics and related discriminating features selected by the classifiers were as follows:

### Performance metrics

**Figure 2:**
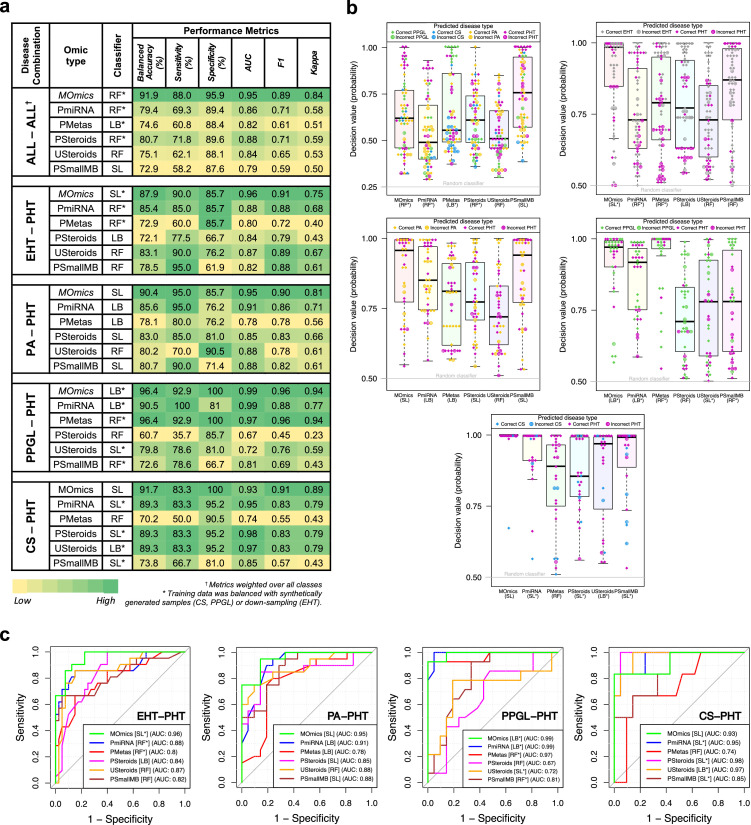
(a) Classification metrics of top-performing classifiers on the test set of 5 disease combinations trained using multi-omics and 5 mono-omics. (b) The prediction performance of top-performing classifiers (on test set) for ALL-ALL and EHT-PHT (top row), PA-PHT and PPGL-PHT (middle row) and CS-PHT (bottom row) combinations. Each symbol represents a test sample. A diamond and crosshair symbol represent a correct and incorrect prediction respectively. The y-axis represents the decision value (probability) of a trained classifier. The value of 0.5 and 0.25 was considered as an outcome of a random classifier for binary (e.g. PA-PHT) and multi-class (e.g. ALL-ALL) data. (c) ROC curves for EHT-PHT, PA-PHT, PPGL-PHT and CS-PHT (left to right) showing the top-performing classifiers and their respective AUC values. The grey line represents the performance of a random classifier.

The MOmics classifier outperformed mono-omics classifiers when considering balanced accuracy, AUC (except CS-PHT), F1, and Kappa score (Figure 2a). Using MOmics data for ALL–ALL combination, RF classifier (trained with balanced dataset) provided better classification performance on test set (∼92% balanced accuracy, 0.95 AUC with 88% sensitivity, and 96% specificity) when compared to other 5 mono-omics. The corresponding decision value (prediction probability) for each test sample was evaluated (Figure 2b). High decision values highlighted the confidence of the classifier in predicting the test sample. Many correctly classified samples had high decision values, which emphasise the fact that MOmics classifier provided better performance in comparison to others. For ALL-ALL, MOmics classifier had 7 incorrectly classified samples (Figure 2b – top row, left). In contrast, the best performing (amongst 5 mono-omics) PSteroids-based RF classifier achieved ∼81% balanced accuracy, 0.88 AUC with ∼72% sensitivity, and ∼90% specificity. The corresponding decision values showed low confidence with 18 incorrectly classified samples. These results were also evaluated as confusion matrices (Appendix Figure 3a).

For the EHT-PHT combination, the SL classifier with MOmics (using balanced data) provided 0.96 AUC (Figure 2c) with 90% sensitivity, and ∼86% specificity (Figure 2a). High decision confidence was observed for most of the correctly classified samples (Figure 2b – top row, right). Although PmiRNA and PMetas-based RF classifier achieved ∼86% specificity (same as MOmics), their AUC was 0.88 and 0.80 respectively. Notably, PSmallMB-based classifier provided the highest sensitivity of 95% however with a lower AUC of 0.82.

For the PA-PHT combination, the SL classifier using MOmics provided highest balanced accuracy and AUC (∼90% and 0.95 respectively) with 95% sensitivity and ∼86% specificity (Figure 2a and c). Although the PmiRNA-based LB classifier provided the highest AUC 0.91 (with 95% sensitivity) amongst mono-omics, USteroids achieved the highest specificity of ∼90%. The decision values highlighted the high confidence of the MOmics classifier in comparison to the others (Figure 2b – middle row, left). In the case of PPGL-PHT combination, the LB classifier using MOmics and RF classifier using PMetas achieved the same balanced accuracy of ∼96% with AUC of 0.99 and 0.97 respectively. The comparative performance of the decision values for these classifiers showed their high confidence (Figure 2b – middle row, right). Also, PmiRNA-based LB classifier provided 0.99 AUC with 81% specificity. Moreover, for the CS-PHT combination, the MOmics-based SL classifier provided 100% specificity and ∼92% balanced accuracy, but with a lower AUC of 0.93. In contrast, mono-omics based classifiers using PSteroids and USteroids achieved higher AUC of 0.98 and 0.97 respectively. The probability values for the test set showed the difference of confidence amongst classifiers (Figure 2b – bottom row).

These classifiers were also tested on the training dataset to understand the effect of overfitting (Appendix Table 6-10). Amongst the three classifiers (LB, RF and SL), evidently RF provided superior classification results when tested on the training set in comparison to the testing set. This highlighted the overfitted training of the RF classifier irrespective of whether the training data was balanced or not. On the other hand, LB and SL classifiers were less overfitted and performed consistently for both training and testing set.

### Discriminating features

**Figure 3:**
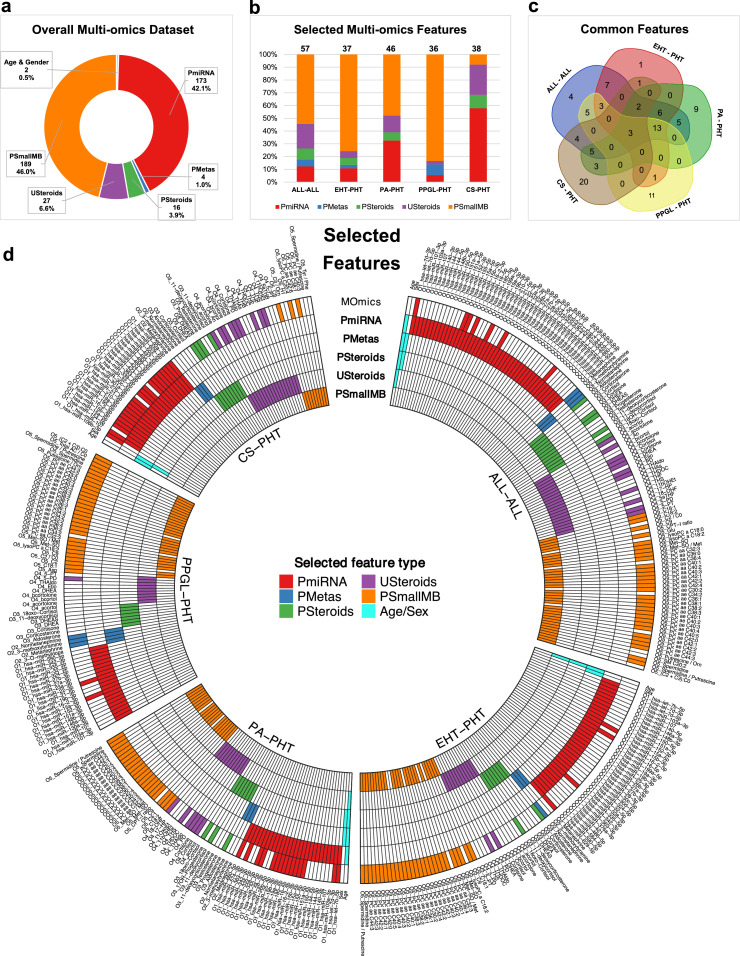
Count and percentage contribution of (a) different omics in the whole multi-omics dataset. (b) Count and percentage contribution of selected features for multi-omics classification within each of 5 disease combinations. Common features amongst disease combinations shown as (c) Venn diagram and (d) Circular heatmap showing the top features selected for the classification of the 5 disease combinations using multi-omics and 5 individual omics.

The final selected set of MOmics features used for classifier training comprised different omics features for each disease combination. The PmiRNA and PSmallMB features represent 88% of the whole MOmics dataset (Figure 3a), a similar share was observed within the final selected set of features (Figure 3b). For example, PSmallMB forms a considerable part of all the disease combinations except CS-PHT where a high number of PmiRNAs were found to be highly discriminating (∼58% of total selected features). In contrast, for PPGL-PHT, very few PmiRNAs (∼5.5% of total features) were selected.

The commonality of selected MOmics features amongst different disease combinations was also investigated (Figure 3c). Two PSmallMB features (O5_PC ae C38:1 and O5_C9) and one PmiRNA (O1_hsa-miR-15a-5p) were present in all five disease combinations (Table 3). Similarly, thirteen PSmallMB features were common amongst 4 disease combinations (i.e. all except CS-PHT). Various unique features were selected for each disease combination. For example, twenty features (15 PmiRNAs, 1 PSteroids, 3 USteroids and 1 PSmallMB) were selected only for CS-PHT. Overall, ALL-ALL has more discriminating features (57 in total) than any of the other four disease combinations. Not unexpectedly, age and sex were not selected in any of the five disease combinations.

**Table 3:** Summary of finally selected MOmics features with regard to disease combinations. The prefix values O1, O2, O3, O4, and O5 represent PmiRNA, PMetas, PSteroids, USteroids, and PSmallMB omics respectively.

Disease Combinations	Total Features	Common Features
ALL - ALL, CS - PHT, EHT - PHT, PA - PHT and PPGL - PHT	3	O1_hsa-miR-15a-5p O5_C9 O5_PC ae C38:1
ALL – ALL, EHT - PHT, PA - PHT and PPGL - PHT	13	O5_Met-SO / Met O5_Spermidine / Putrescine O5_PC ae C40:4 O5_Spermidine O5_PC ae C38:3 O5_PC ae C40:5 O5_PC ae C44:3 O5_PC ae C42:2 O5_PC ae C36:1 O5_PC ae C40:3 O5_Met-SO O5_PC aa C32:3 O5_PC ae C38:2
ALL - ALL, CS - PHT, EHT - PHT and PA - PHT	2	O3_11-deoxycortisol O1_hsa-miR-16-2-3p
ALL – ALL, EHT - PHT and PA - PHT	6	O5_PC ae C40:2 O5_PC aa C40:3 O3_Aldosterone O5_PC ae C42:1 O5_PC aa C42:4 O4_THAldo
ALL - ALL, EHT - PHT and PPGL - PHT	3	O5_C18:1 O5_PC aa C42:2 O2_Normetanephrine
ALL - ALL, CS - PHT and PA - PHT	5	O4_PD O1_hsa-miR-19a-3p O4_Cortisone O4_Cortisol O4_THS
ALL - ALL and EHT - PHT	7	O5_PC aa C42:1 O5_PC ae C40:1 O5_PC aa C40:2 O4_DHEA O5_PC ae C30:2 O5_PC ae C42:3 O5_PC aa C40:1
ALL - ALL and PA - PHT	5	O4_18-OHF O3_18oxo-Cortisol O1_hsa-miR-155-5p O5_C18:2 O1_hsa-let-7d-3p
ALL - ALL and PPGL - PHT	5	O2_3-methoxytyramine O4_5-PD O2_Metanephrine O5_lysoPC a C18:2 O1_hsa-miR-22-3p
ALL - ALL and CS - PHT	4	O4_An O3_DHEAS O4_acortol O3_DHEA
EHT - PHT and PPGL - PHT	1	O5_PC ae C42:0
CS - PHT and EHT - PHT	1	O1_hsa-miR-629-5p
CS - PHT and PA - PHT	3	O1_hsa-miR-32-5p O1_hsa-miR-15b-3p O1_hsa-miR-363-3p
ALL - ALL	4	O5_Glu O1_hsa-miR-423-5p O4_5-PT O5_PC aa C36:0
EHT - PHT	1	O1_hsa-miR-335-5p
PA - PHT	9	O1_hsa-miR-25-3p O1_hsa-miR-16-5p O5_C4:1 O1_hsa-miR-19b-3p O5_PC aa C34:3 O1_hsa-miR-660-5p O1_hsa-miR-185-5p O1_hsa-let-7b-5p O1_hsa-miR-328-3p
PPGL - PHT	11	O5_(C2 + C3):C0 O5_PC aa C36:4 O5_Putrescine / Orn O5_C2 O5_PC ae C34:2 O5_Asp O5_PC ae C34:3 O5_PC ae C36:3 O5_C2 / C0 O5_PC aa C36:2 O5_Total AC / C0
CS - PHT	20	O1_hsa-miR-495-3p O1_hsa-miR-485-3p O1_hsa-miR-107 O1_hsa-miR-21-5p O1_hsa-miR-497-5p O1_hsa-miR-486-5p O3_Progesterone O1_hsa-miR-210-3p O4_THDOC O1_hsa-miR-106b-3p O5_lysoPC a C20:4 O4_bcortol O1_hsa-miR-92a-3p O1_hsa-miR-301a-3p O1_hsa-miR-27b-3p O4_THF O1_hsa-miR-424-5p O1_hsa-miR-339-5p O1_hsa-miR-502-3p O1_hsa-miR-195-5p

The discriminating features selected for mono-omics classifiers were also examined (Figure 3d). Age and sex were selected in most of the mono-omics classifiers (except PSmallMB) in all disease combinations other than PPGL-PHT. A higher number of PmiRNAs were selected in the case of ALL-ALL and EHT-PHT in comparison to other disease combinations. On the contrary, for PSmallMB classifier only 9 features were selected in CS-PHT amongst all disease combinations.

The contribution of each omic in the final selected MOmics features (with regard to the count in the whole dataset) was also analysed. For example, 3 out of 4 PMetas features (75%) were selected in ALL–ALL and PPGL-PHT disease combinations. None of the PSteroids features was selected in PPGL-PHT. No PMetas were selected in PA-PHT and CS-PHT combinations (Figure 3d).

The close examination of features amongst MOmics and mono-omics classifiers highlighted that all the features selected in MOmics were part of the individual omics classifiers, except PSmallMB and PmiRNA (Figure 3d). In case of PSmallMB, some features were exclusively selected in MOmics classifiers (For example, O5_PC aa C34:3 and O5_PC ae C40:2 in PA-PHT). Similarly, in CS-PHT, PmiRNA O1_hsa−miR−106b−3p was exclusively selected for MOmics classifier.

The MOmics features selected in ALL-ALL disease combination were compared with corresponding omic features for NV in the training set (Appendix Figure 4). Also, Principal Component Analysis was conducted for all five disease combinations alongside NV (Appendix Figure 5). The first component of ALL-ALL and EHT-PHT accounted for ∼40% and 57% of the explained variance respectively.

### In-depth analysis of primary and endocrine hypertension

The training set of the MOmics data was studied for a different set of scenarios (Table 2). These scenarios include the use of age and sex as features and understanding the effect of age and sex segregated subsets on feature selection in different disease combinations (See stage 2 in Figure 1).

### Scenario 1: including (Set A) vs excluding (Set B) age and sex as features

For Scenario 1, MOmics data provided better performance for all disease combinations (Appendix Figure 6). In intra-set comparison, MOmics achieved similar performance in Sets A and B across all disease combinations. Hence, excluding age and sex (in Set B) as features did not materially alter the performance of the classification. However, for PMetas, balanced accuracy dropped when age and sex were excluded (Set B) from the feature set (except for PPGL-PHT). For example, balanced accuracy was down by 5% and 7% for ALL-ALL and EHT-PHT respectively.

The remaining four mono-omics provided comparable performance irrespective of age and sex being used as features. For example, in the case of USteroids, the detailed summary of features selected during the 100 RR show that almost the same features are selected approximately equal number of times for Set A and B (Appendix Figure 7c). Similar trends were observed for other mono-omics (Appendix Figures 7 – 9). Notably, for MOmics despite including age and sex as features (Set A), the selection frequencies were below the threshold in 100 RR and therefore they were not designated as top features (Appendix Figure 10).

### Scenario 2: males (Set C) vs females (Set D)

The classification performance of Sets C and D was not comparable since they used different numbers of samples for training and testing. However, it is noticeable that the female subset provided better accuracy for EHT-PHT and PA-PHT in comparison to male subset for MOmics data (Appendix Figure 6). The intra-set comparison highlighted the superior performance of MOmics dataset for ALL-ALL and EHT-PHT disease combinations in comparison to mono-omics irrespective of classifier selection. For PPGL-PHT, PMetas outperformed the MOmics classification for both the Sets. However, in case of CS-PHT, Set C could not be run due to an insufficient number of male CS samples for classifier training (Appendix Table 4).

From the perspective of feature selection, in the MOmics dataset, different features were selected for Sets C and D (Appendix Figure 10). For example, in ALL-ALL, O1_hsa-miR-15a-5p, O5_Spermidine and O5_Spermidine/Putrescine were only selected for male dataset. On the contrary, various other features such as O5_PC ae C38:1, O3_18oxo-Cortisol, and O4_18-OHF were only selected for female dataset. On close examination, it was evident that the union of Set C and Set D features approximately intersect with both Sets A and B. Similar trends were also observed across most of the disease combinations in MOmics and mono-omics datasets (Appendix Figures 7 – 9).

### Scenario 3: older (Set E) vs younger (Set F)

Overall, MOmics data provided better classification performance in comparison to mono-omics (except PPGL-PHT), irrespective of the cohort age (Appendix Figure 6). When considering the inter-set comparison, Set E (age >= 50) provided better results than Set F (age < 50) for almost all disease combinations. A higher number of unique features were selected for both the cohorts for all disease combinations (Appendix Figure 10). Similar trends were noticed for mono-omics datasets (Appendix Figures 7 – 9).

## Discussion

This study investigates MOmics ML integration for stratification of arterial hypertension. Our results show that the MOmics approach provided improved discriminatory power in comparison to single omics (mono-omics) data analysis and was able to correctly identify different forms of endocrine hypertension with high sensitivity and specificity, providing potential diagnostic biomarker combinations for diagnosing hypertension subtypes. In particular, it was able to classify subtypes of hypertension with 96% specificity and 0.95 AUC in the ALL vs ALL disease combination, achieving ∼11% improvement in balanced accuracy on an unseen test set in comparison to the best performing mono-omics classifier using a reduced set of 57 features. The feature set had distinct characteristics when compared with normotensive volunteers and hence can be potentially utilised as biomarker in clinical application.

With the availability of recent high-throughput experimental and computational technologies, ML-based integration will facilitate the discovery of biomarkers for diagnosis and improve the understanding of complex diseases such as arterial hypertension. However, obtaining MOmics data can be logistically challenging when biosamples are sourced from multiple recruitment sites and require multi-centre omics measurements. This can lead to fewer samples with all available omics for integration. The ENS@T-HT study, by obtaining a complete set of omics for ∼84% of the total patients, provided a straightforward example that this challenge can be successfully addressed. Although a few mono-omics studies on identification of endocrine forms of hypertension have been published,^32^^,^^49^ to the best of our knowledge, no other study exists that collected and analysed MOmics data for hypertension stratification and predicting hypertension subtypes.

This study predicted EHT subtypes using a dedicated and customisable ML pipeline. The imbalance of classes is a well-known problem in ML which does not allow the classifier to learn from the minority class. This was corrected for CS and PPGL patients with the use of Synthetic Minority Over-sampling TEchnique.^48^ Evaluating classification performance was one of the key outcomes for this study. The method used also enabled the assessment of top discriminating features and comparison of these to the NV.

Despite the strong classification performance, the analysis had a few shortcomings. Firstly, since CS is a rare disease, samples for CS patients were limited. Secondly, advanced ML techniques such as deep learning could not be used for this analysis as they require a much larger number of samples than was available in this study. Finally, all the samples could not be used for MOmics integration because of limitations in sample volume or specific quality measures, which is a common problem for a study with multi-site biosamples and multi-centre omics measurement. However, a major strength of this study was to rely on unambiguous diagnosis of the major subtypes of EHT according to guidelines by expert centres. In addition, our analysis only explored the MOmics data using a ML based data-driven approach. The discovered top discriminating features need further investigation in terms of biological significance and pathway network analysis.

This study provided a valuable insight into a complex multi-class problem of endocrine hypertension stratification. It uses multi-omics data and evaluated its superior performance in comparison to mono-omics. Although internal validation was conducted using an unseen test set, further validation on external cohorts before any translation in clinical setting will be valuable. The ENS@T-HT study is currently capturing data from a wider population in a prospective manner to measure the most discriminating features of the new samples and perform independent validation (ClinicalTrials.gov Identifier: NCT02772315).This would allow the classifier to become more robust and well-trained for a formal clinical deployment. The refined algorithm could be deployed as a webserver-based classification tool and utilised to screen patients at primary care to refer patients identified as being at risk of endocrine hypertension to centres with appropriate expertise for subsequent evaluation if required. The developed ML pipeline is fully customisable and can be deployed for other mono/MOmics data- based biomarker discovery and analysis studies. For example, it can be used to investigate MOmics signatures for other forms of secondary hypertension such as renal artery stenosis.

To conclude, in this study we have developed an innovative approach to predict different subtypes of arterial hypertension using multi-omics data. A machine learning pipeline using 5 disease combinations and 8 supervised ML classifiers was introduced and scenarios evaluated based on age and sex bifurcation. The ML pipeline provided promising classification outcomes and the reduced features have the potential to further contribute as clinical biomarkers for detecting hypertension subtypes. This is expected to improve stratification of patients with hypertension for implementation of targeted treatment and prevention of cardiovascular complications.

## Contributors

All authors read and approved the final version of the manuscript. M-C.Z., E.J., E.D. and P.S.R. conceived and designed the study. P.S.R. and S.Reel developed and implemented ML pipeline under the supervision of E.J.. J.C.v.K, K.Lang, C.K.L., C.Pamporaki, S.M.M., A.E.T., M.P., J.D.M, C.Prehn, J.A., G.E., E.D. developed the methodology for omics data generation. J.C.v.K., S.Robertson, S.M.M., J.D.M. and A.R. used GENEX software for miRNA data. T.N. and A.S. developed software for cataloguing the omics (RDMP) and phenotypic data (ENSAT-HT registry) respectively. P.S.R., S.Reel, J.C.v.K., K.Lang, C.K.L., S.Robertson, S.M.M., A.E.T., M.P., J.D.M., A.R., C.Prehn, J.A., M-C.Z., E.D. and E.J. validated the various phases of the study. J.C.v.K., K.Langton, K.Lang, Z.E., C.K.L., C.Pamporaki, S.Robertson, A.E.T., M.P., J.D.M., A.R., C.Prehn, J.A. and E.D. performed formal analysis and investigation on omics data. K.Langton, Z.E., L.A., C.Pamporaki, P.M., A.B., M.Kabat, M.P., F.C., C.S., M.R., M.Kroiss, M.C.D., A.Pecori, S.M., J.D., G.P.R., L.L., I.S., A-P.G-R., G.A., F.B., G.E. and E.D. provided resources such as bio-samples for omics data. P.S.R., S.Reel, K.Langton, K.Lang, Z.E., C.Pamporaki, M.P., W.A.and G.E. curated the omics data. P.S.R, M-C.Z. and E.J. verified the data. P.S.R. and S.Reel wrote the initial draft of the manuscript with assistance from M-C.Z. and E.J. The details of methodology of omics data generation was provided by Z.E., S. Robertson, S.M.M., M.P., W.A., G.E., E.D. and M-C.Z.. The following drafts were reviewed and edited by P.S.R., S.Reel, Z.E., L.A., P.M., A.B., S.Robertson, S.M.M., M.P., M.R., M.Kroiss, J.D., G.P.R., L.L., J.D.M., A.R., C.C., W.A., F.B., G.E., E.D.,M-C.Z. and E.J.. P.S.R. and S.Reel drafted the data visualisations. S.M.M., W.A., G.E., E.D., M-C.Z. and E.J. supervised the study. P.S.R., L.A., P.M., A.B., S.M.M., W.A., F.B., G.E., E.D., M-C.Z. and E.J. were involved in the planning and execution of this study. L.A., P.M., S.M.M., M.R., J.D., G.P.R., J.D.M., W.A., F.B., G.E., E.D., M-C.Z. and E.J. acquired the financial support for the ENSAT-HT project leading to the work in this manuscript.

## Data sharing statement

The data related to the results presented in this article can be obtained upon reasonable request. The data can be made available to the researchers who provide a methodologically sound proposal, subject to ENSAT-HT executive committee's approval (which includes representatives of the biosamples collections and omics data generation centres). The researcher would need to complete a Data Sharing Agreement. All requests should be directed by email to the corresponding author. The study used well-established computational packages and libraries as referenced in the Methods section. The codebase used in this study is available online (https://doi.org/10.5281/zenodo.7033087).

## Declaration of interests

P.S.R., S.Reel, K.Lang, A.E.T., M.R., W.A., F.B., G.E., M-C.Z. and E.J. are listed as co-inventors on patents filed related to Biomarkers for Diagnosis and Treatment of Endocrine Hypertension, and Methods of Identification thereof.

P.S.R., S.R., J.C.v.K., K.Langton, K.Lang, Z.E., C.K.L., L.A., P.M., A.B., M.Kabat, S.Robertson, S.M.M., A.E.T., M.P., F.C., M.Kroiss, M.C.D., S.M., J.D., G.P.R., L.L., J.D.M., A.R., A.S., I.S., J.A., A-P.G-R., G.A., W.A., F.B., G.E., E.D., M-C.Z. and E.J.reports grants from European Union's Horizon 2020 Research and Innovation Programme under Grant Agreement No. 633983, during the conduct of the study

K. Langton reports grants from CRC/TRR 205 Project B 15 and Patient Cohorts and Biobanks Fonds 041_526513, outside the submitted work. P.M. reports personal fees from DIASORIN, outside the submitted work. J.D. reports grants from Idorsia and Damian Pharma, outside the submitted work. G.E. reports grant from DFG, outside the submitted work.

The other authors declare no conflict of interest.

## Appendix Supplementary materials

Image, application 1

Image, application 2

## References

[1] Rahimi K, Emdin CA, MacMahon S. (2015). The epidemiology of blood pressure and its worldwide management. Circul Res.

[2] Zhou B, Bentham J, Di Cesare M (2017). Worldwide trends in blood pressure from 1975 to 2015: a pooled analysis of 1479 population-based measurement studies with 19·1 million participants. Lancet.

[3] Lewington S, Clarke R, Qizilbash N, Peto R, Collins R, Prospective Studies Collaboration (2002). Age-specific relevance of usual blood pressure to vascular mortality: a meta-analysis of individual data for one million adults in 61 prospective studies. Lancet.

[4] Members AF, Mancia G, Fagard R (2013). 2013 ESH/ESC Guidelines for the management of arterial hypertension the task force for the management of arterial hypertension of the European Society of Hypertension (ESH) and of the European Society of Cardiology (ESC). Eur Heart J.

[5] Rimoldi SF, Scherrer U, Messerli FH. (2014). Secondary arterial hypertension: when, who, and how to screen?. Eur Heart J.

[6] 2018 ESC/ESH Guidelines for the management of arterial hypertension | European Heart Journal | Oxford Academic. https://academic.oup.com/eurheartj/article/39/33/3021/5079119. Accessed 4 March 2020.

[7] Hannemann A, Wallaschofski H. (2012). Prevalence of primary aldosteronism in patient's cohorts and in population-based studies – a review of the current literature. Horm Metab Res.

[8] Monticone S, Burrello J, Tizzani D (2017). Prevalence and clinical manifestations of primary aldosteronism encountered in primary care practice. J Am Coll Cardiol.

[9] Rossi GP, Bernini G, Caliumi C (2006). A prospective study of the prevalence of primary aldosteronism in 1,125 hypertensive patients. J Am Coll Cardiol.

[10] Rossi GP, Rossitto G, Amar L (2022). Drug-resistant hypertension in primary aldosteronism patients undergoing adrenal vein sampling: the AVIS-2-RH study. Eur J Prev Cardiol.

[11] Touyz RM, Burger D. (2012). Special Issues in Hypertension.

[12] Olsen MH, Angell SY, Asma S (2016). A call to action and a lifecourse strategy to address the global burden of raised blood pressure on current and future generations: the Lancet commission on hypertension. Lancet.

[13] Velema MS, Nooijer AH de, Burgers VWG (2017). Health-related quality of life and mental health in primary aldosteronism: a systematic review. Horm Metab Res.

[14] Lim YY, Shen J, Fuller PJ, Yang J. (2018). Current pattern of primary aldosteronism diagnosis: delayed and complicated. Aust J Gen Pract.

[15] Barh D, Blum K, Madigan MA (2011).

[16] Reel PS, Reel S, Pearson E, Trucco E, Jefferson E (2021). Using machine learning approaches for multi-omics data analysis: a review. Biotechnol Adv.

[17] Sharifi-Noghabi H, Zolotareva O, Collins CC, Ester M. (2019). MOLI: multi-omics late integration with deep neural networks for drug response prediction. Bioinformatics.

[18] Xicota L, Ichou F, Lejeune F-X (2019). Multi-omics signature of brain amyloid deposition in asymptomatic individuals at-risk for Alzheimer's disease: the INSIGHT-preAD study. EBioMedicine.

[19] ENSAT-HT Project. ENSAT-HT Project. http://www.ensat-ht.eu/. Accessed 26 February 2020.

[20] Funder JW, Carey RM, Mantero F (2016). The management of primary aldosteronism: case detection, diagnosis, and treatment: an endocrine society clinical practice guideline. J Clin Endocrinol Metab.

[21] Mulatero P, Monticone S, Deinum J (2020). Genetics, prevalence, screening and confirmation of primary aldosteronism: a position statement and consensus of the working group on endocrine hypertension of the european society of hypertension∗. J Hypertens.

[22] Lenders JWM, Duh Q-Y, Eisenhofer G (2014). Pheochromocytoma and paraganglioma: an endocrine society clinical practice guideline. J Clin Endocrinol Metab.

[23] Nieman LK, Biller BMK, Findling JW (2008). The diagnosis of cushing's syndrome: an endocrine society clinical practice guideline. J Clin Endocrinol Metab.

[24] Andersen CL, Jensen JL, Ørntoft TF. (2004). Normalization of real-time quantitative reverse transcription-PCR data: a model-based variance estimation approach to identify genes suited for normalization, applied to bladder and colon cancer data sets. Cancer Res.

[25] Peitzsch M, Prejbisz A, Kroiß M (2013). Analysis of plasma 3-methoxytyramine, normetanephrine and metanephrine by ultraperformance liquid chromatographytandem mass spectrometry: utility for diagnosis of dopamine-producing metastatic phaeochromocytoma. Ann Clin Biochem.

[26] Peitzsch M, Adaway JE, Eisenhofer G. (2015). Interference from 3-O-methyldopa with ultra–high performance LC-MS/MS measurements of plasma metanephrines: chromatographic separation remains important. Clin Chem.

[27] Peitzsch M, Dekkers T, Haase M (2015). An LC–MS/MS method for steroid profiling during adrenal venous sampling for investigation of primary aldosteronism. J Steroid Biochem Mol Biol.

[28] Sagmeister MS, Taylor AE, Fenton A (2019). Glucocorticoid activation by 11β-hydroxysteroid dehydrogenase enzymes in relation to inflammation and glycaemic control in chronic kidney disease: a cross-sectional study. Clin Endocrinol.

[29] Bancos I, Taylor AE, Chortis V (2020). Urine steroid metabolomics for the differential diagnosis of adrenal incidentalomas in the EURINE-ACT study: a prospective test validation study. Lancet Diabetes Endocrinol.

[30] Römisch-Margl W, Prehn C, Bogumil R, Röhring C, Suhre K, Adamski J. (2012). Procedure for tissue sample preparation and metabolite extraction for high-throughput targeted metabolomics. Metabolomics.

[31] Zukunft S, Sorgenfrei M, Prehn C, Möller G, Adamski J. (2013). Targeted metabolomics of dried blood spot extracts. Chromatographia.

[32] Erlic Z, Reel P, Reel S (2021). Targeted metabolomics as a tool in discriminating endocrine from primary hypertension. J Clin Endocrinol Metab.

[33] Nind T, Galloway J, McAllister G (2018). The research data management platform (RDMP): a novel, process driven, open-source tool for the management of longitudinal cohorts of clinical data. GigaScience.

[34] Breiman L (1998).

[35] Zhang H. (2004). In FLAIRS2004 conference.

[36] Bentley JL. (1975). Multidimensional binary search trees used for associative searching. Commun ACM.

[37] Friedman J, Hastie T, Tibshirani R. (1998). Additive logistic regression: a statistical view of boosting. Ann Stat.

[38] Landwehr N, Hall M, Frank E. (2005). Logistic model trees. Mach Learn.

[39] Sumner M, Frank E, Hall M. (2005). Proceedings of the 9th European conference on European conference on machine learning and principles and practice of knowledge discovery in databases.

[40] Breiman L. (2001). Random forests. Mach Learn.

[41] Platt J. Fast training of support vector machines using sequential minimal optimization. 1998; published online January 1. https://www.microsoft.com/en-us/research/publication/fast-training-of-support-vector-machines-using-sequential-minimal-optimization/. Accessed 26 February 2020.

[42] Kuhn M. (2008). Building predictive models in R using the caret package. J Stat Softw.

[43] Hornik K, Buchta C, Zeileis A. (2009). Open-source machine learning: R meets Weka. Comput Stat.

[44] R Core Team (2013). http://www.R-project.org/.

[45] Reel P, Reel S, Cole C, Zenaro M-C, Jefferson E (2022). MOmicsML: v0.0.1-beta - Multi-omics ML predictor for endocrine hypertension. Zenodo.

[46] Kursa MB, Rudnicki WR. (2010). Feature selection with the Boruta package. J Stat Softw.

[47] Hall MA. Correlation-based Feature selection for machine learning. 1999.

[48] Chawla NV, Bowyer KW, Hall LO, Kegelmeyer WP. (2002). SMOTE: synthetic minority over-sampling technique. J Artif Intell Res.

[49] Eisenhofer G, Durán C, Cannistraci CV (2020). Use of steroid profiling combined with machine learning for identification and subtype classification in primary aldosteronism. JAMA Netw Open.

