# Supplementary material for: Machine learning for classification of hypertension subtypes using multi-omics: A multi-centre, retrospective, data-driven study
Source: eBioMedicine. 2022 Sep 27;84:104276. doi: 10.1016/j.ebiom.2022.104276 (PMC9520210; doi:10.1016/j.ebiom.2022.104276)
Supplement: Supplementary file 1 [file mmc1.pdf]

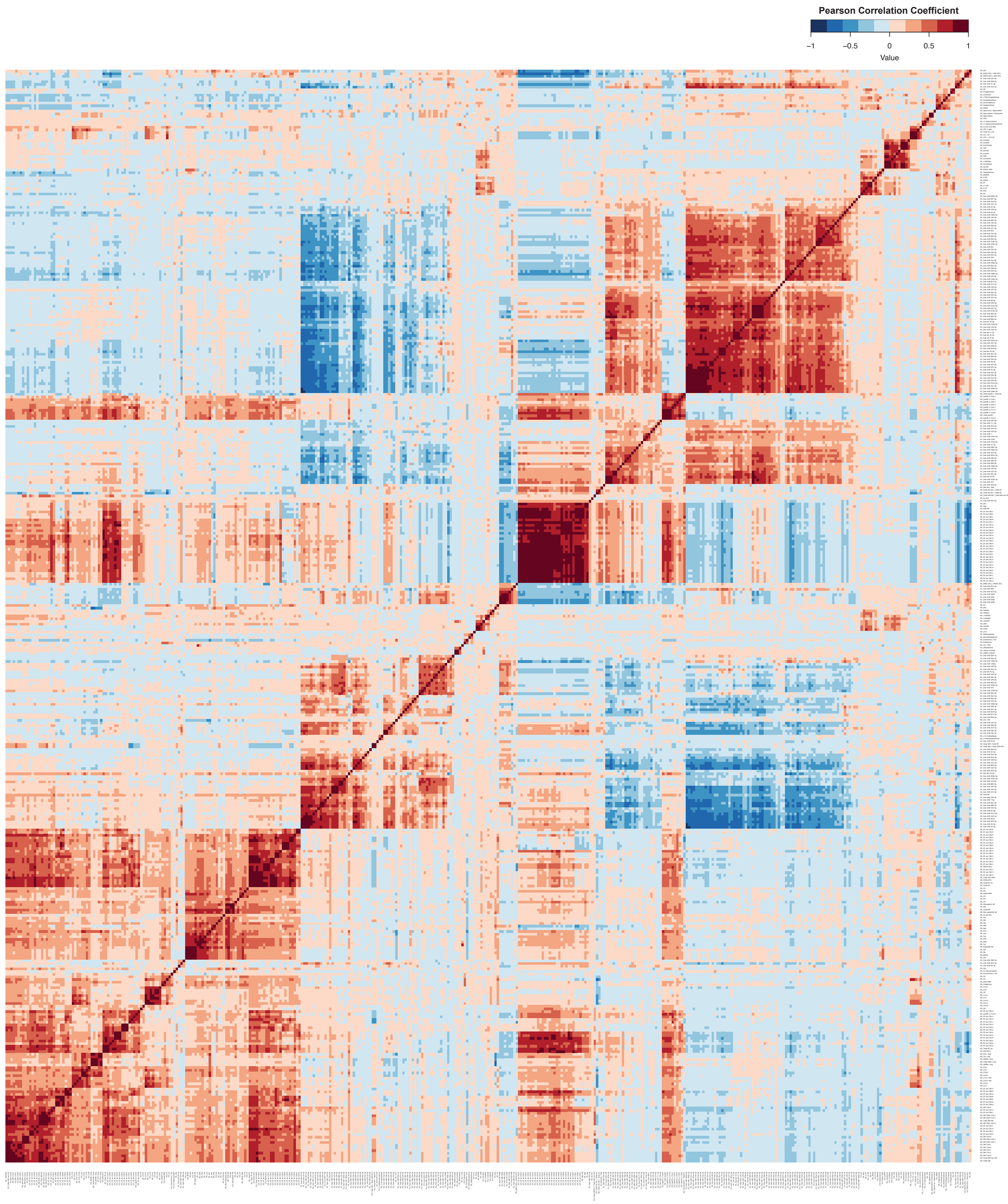

Supplementary Figure 1. The pairwise relationship amongst all multi-omics features shown as clustered Pearson correlation coefficients.

a

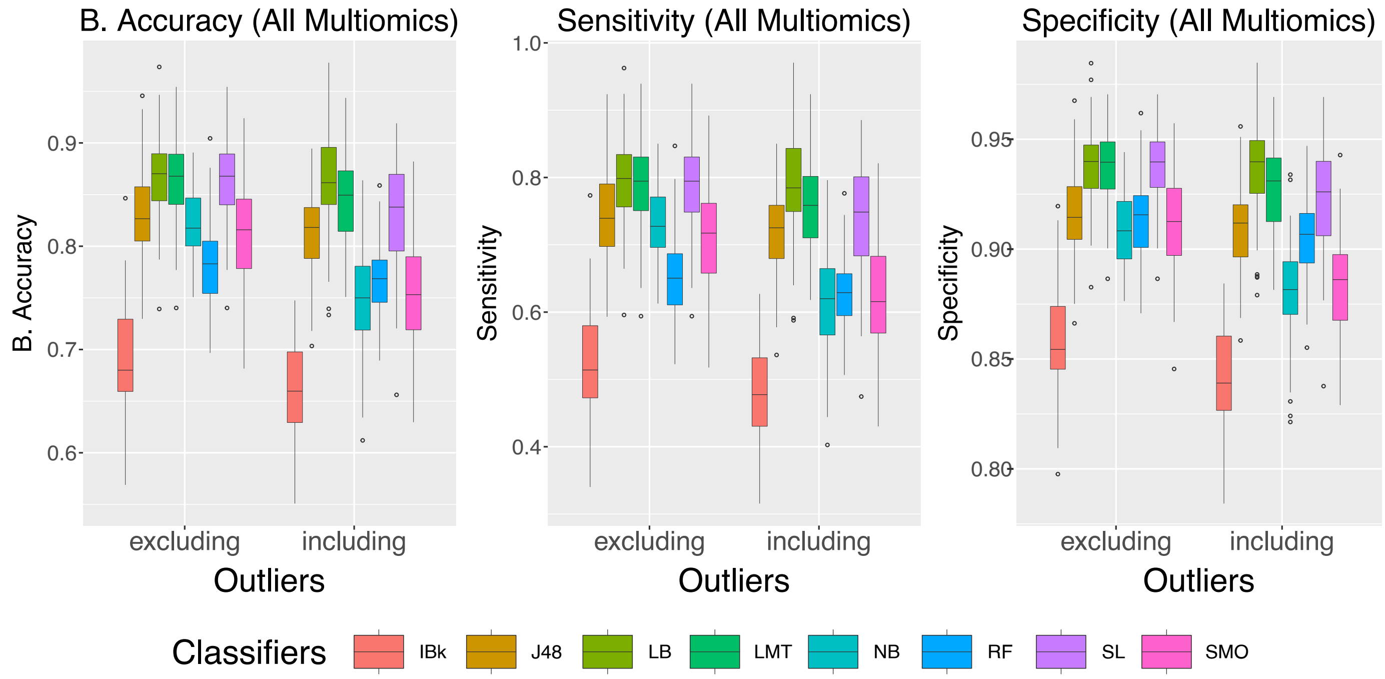

b

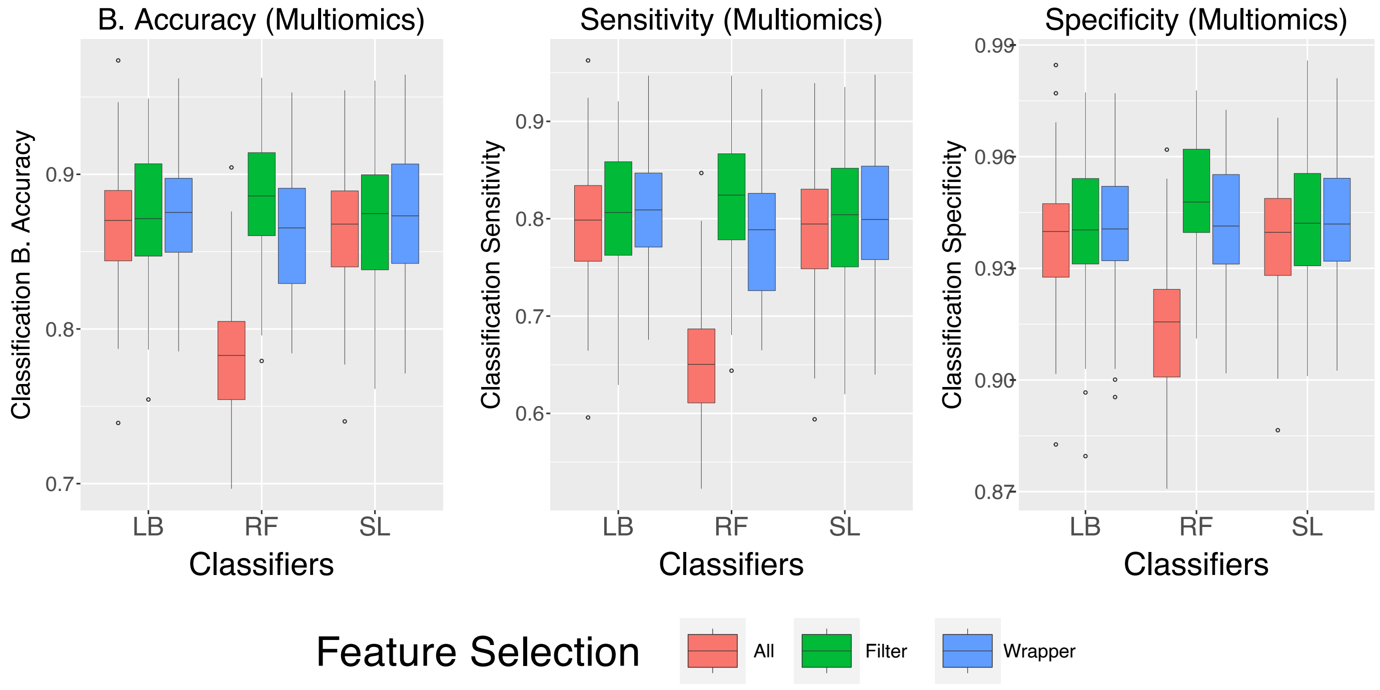

Supplementary Figure 2. Classification results for evaluating (a) best classifier and (b) best feature selection method on ALL-ALL disease combination using training set of multi-omics data.

a

Predicted \ Actual	PPGL	CS	PA	PHT	Σ
PPGL	13	1	0	1	15
CS	0	5	0	0	5
PA	0	0	19	3	22
PHT	1	0	1	17	19
Σ	14	6	20	21	61

MOmics **(RF*)**

Predicted \ Actual	PPGL	CS	PA	PHT	Σ
PPGL	9	1	2	0	12
CS	0	4	1	0	5
PA	4	1	15	6	26
PHT	1	0	2	15	18
Σ	14	6	20	21	61

PmiRNA **(RF*)**

Predicted \ Actual	PPGL	CS	PA	PHT	Σ
PPGL	13	0	0	0	13
CS	0	2	6	6	14
PA	1	2	11	2	16
PHT	0	2	3	13	18
Σ	14	6	20	21	61

PMetas **(LB*)**

Predicted \ Actual	PPGL	CS	PA	PHT	Σ
PPGL	8	0	0	4	12
CS	1	5	1	0	7
PA	1	1	17	4	23
PHT	4	0	2	13	19
Σ	14	6	20	21	61

PSteroids **(RF*)**

Predicted \ Actual	PPGL	CS	PA	PHT	Σ
PPGL	6	3	2	0	11
CS	0	3	0	0	3
PA	3	0	14	3	20
PHT	5	0	4	18	27
Σ	14	6	20	21	61

USteroids **(RF)**

Predicted \ Actual	PPGL	CS	PA	PHT	Σ
PPGL	6	0	1	1	8
CS	1	2	0	1	4
PA	5	2	17	4	28
PHT	2	2	2	15	21
Σ	14	6	20	21	61

PSmallMB **(SL)**

b

Predicted \ Actual	EHT	PHT	Σ
EHT	36	3	39
PHT	4	18	22
Σ	40	21	61

MOmics **(SL*)**

Predicted \ Actual	EHT	PHT	Σ
EHT	34	3	37
PHT	6	18	24
Σ	40	21	61

PmiRNA **(RF*)**

Predicted \ Actual	EHT	PHT	Σ
EHT	24	3	27
PHT	16	18	34
Σ	40	21	61

PMetas **(RF*)**

Predicted \ Actual	EHT	PHT	Σ
EHT	31	7	38
PHT	9	14	23
Σ	40	21	61

PSteroids **(LB)**

Predicted \ Actual	EHT	PHT	Σ
EHT	36	5	41
PHT	4	16	20
Σ	40	21	61

USteroids **(RF)**

Predicted \ Actual	EHT	PHT	Σ
EHT	38	8	46
PHT	2	13	15
Σ	40	21	61

PSmallMB **(RF)**

c

Predicted \ Actual	PA	PHT	Σ
PA	19	3	22
PHT	1	18	19
Σ	20	21	41

MOmics **(SL)**

Predicted \ Actual	PA	PHT	Σ
PA	19	5	24
PHT	1	16	17
Σ	20	21	41

PmiRNA **(LB)**

Predicted \ Actual	PA	PHT	Σ
PA	16	5	21
PHT	4	16	20
Σ	20	21	41

PMetas **(LB)**

Predicted \ Actual	PA	PHT	Σ
PA	17	4	21
PHT	3	17	20
Σ	20	21	41

PSteroids **(SL)**

Predicted \ Actual	PA	PHT	Σ
PA	14	2	16
PHT	6	19	25
Σ	20	21	41

USteroids **(RF)**

Predicted \ Actual	PA	PHT	Σ
PA	18	6	24
PHT	2	15	17
Σ	20	21	41

PSmallMB **(SL)**

d

Predicted \ Actual	PPGL	PHT	Σ
PPGL	13	0	13
PHT	1	21	22
Σ	14	21	35

MOmics **(LB*)**

Predicted \ Actual	PPGL	PHT	Σ
PPGL	14	4	18
PHT	0	17	17
Σ	14	21	35

PmiRNA **(LB*)**

Predicted \ Actual	PPGL	PHT	Σ
PPGL	13	0	13
PHT	1	21	22
Σ	14	21	35

PMetas **(RF*)**

Predicted \ Actual	PPGL	PHT	Σ
PPGL	5	3	8
PHT	9	18	27
Σ	14	21	35

PSteroids **(RF)**

Predicted \ Actual	PPGL	PHT	Σ
PPGL	11	4	15
PHT	3	17	20
Σ	14	21	35

USteroids **(SL*)**

Predicted \ Actual	PPGL	PHT	Σ
PPGL	11	7	18
PHT	3	14	17
Σ	14	21	35

PSmallMB **(RF*)**

e

Predicted \ Actual	CS	PHT	Σ
CS	5	0	5
PHT	1	21	22
Σ	6	21	27

MOmics **(SL)**

Predicted \ Actual	CS	PHT	Σ
CS	5	1	6
PHT	1	20	21
Σ	6	21	27

PmiRNA **(SL*)**

Predicted \ Actual	CS	PHT	Σ
CS	3	2	5
PHT	3	19	22
Σ	6	21	27

PMetas **(RF)**

Predicted \ Actual	CS	PHT	Σ
CS	5	1	6
PHT	1	20	21
Σ	6	21	27

PSteroids **(SL*)**

Predicted \ Actual	CS	PHT	Σ
CS	5	1	6
PHT	1	20	21
Σ	6	21	27

USteroids **(LB*)**

Predicted \ Actual	CS	PHT	Σ
CS	4	4	8
PHT	2	17	19
Σ	6	21	27

PSmallMB **(SL*)**

Supplementary Figure 3. Confusion matrices of top performing classifiers on test set for (a) ALL-ALL, (b) EHT-PHT, (c) PA-PHT, (d) PPGL-PHT and (e) CS-PHT disease combination.

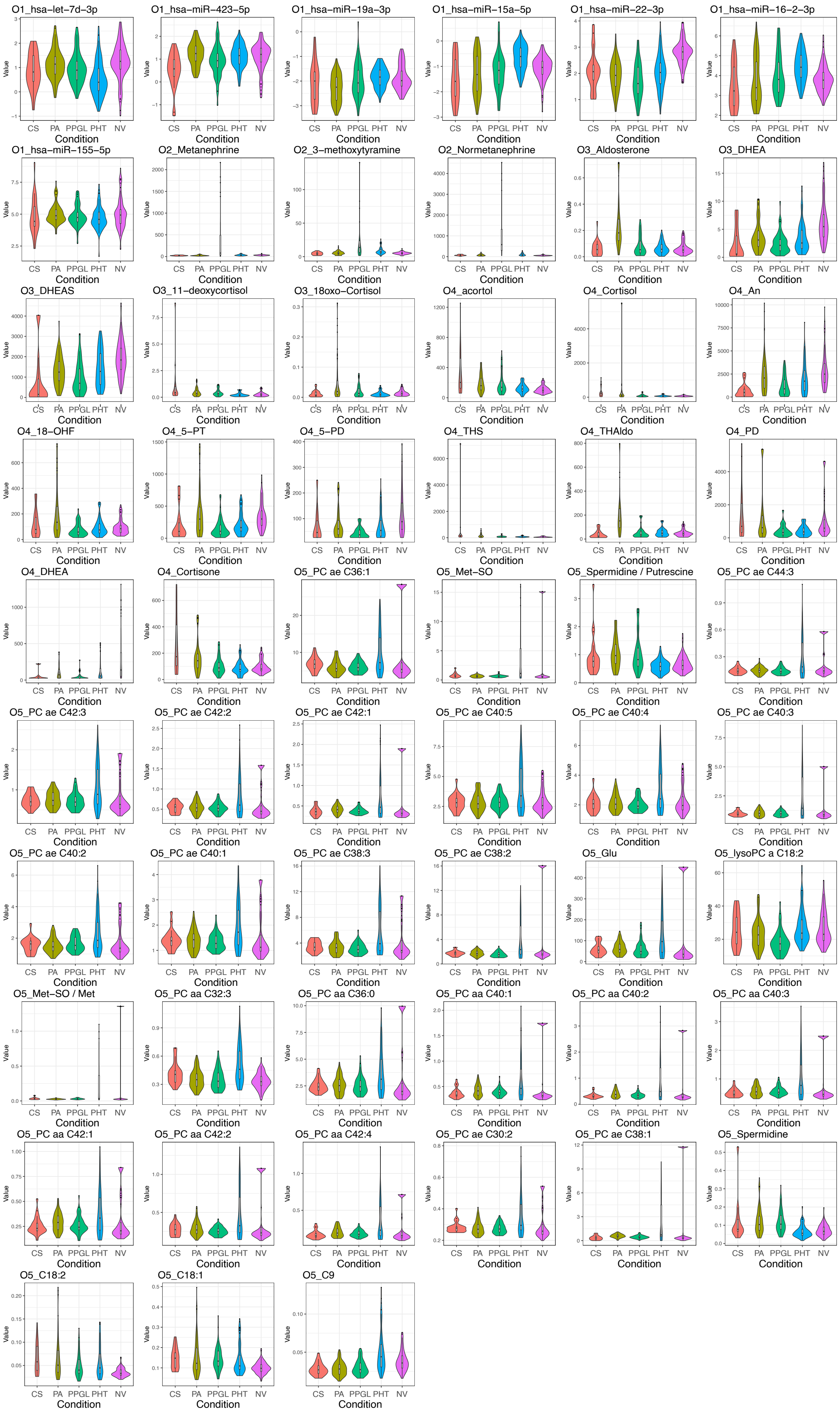

Supplementary Figure 4. Violin plots of most discriminating PmiRNA, PMetas, PSteroids and USteroids features selected for ALL-ALL disease combination in multi-omics classifier and corresponding values for NV.

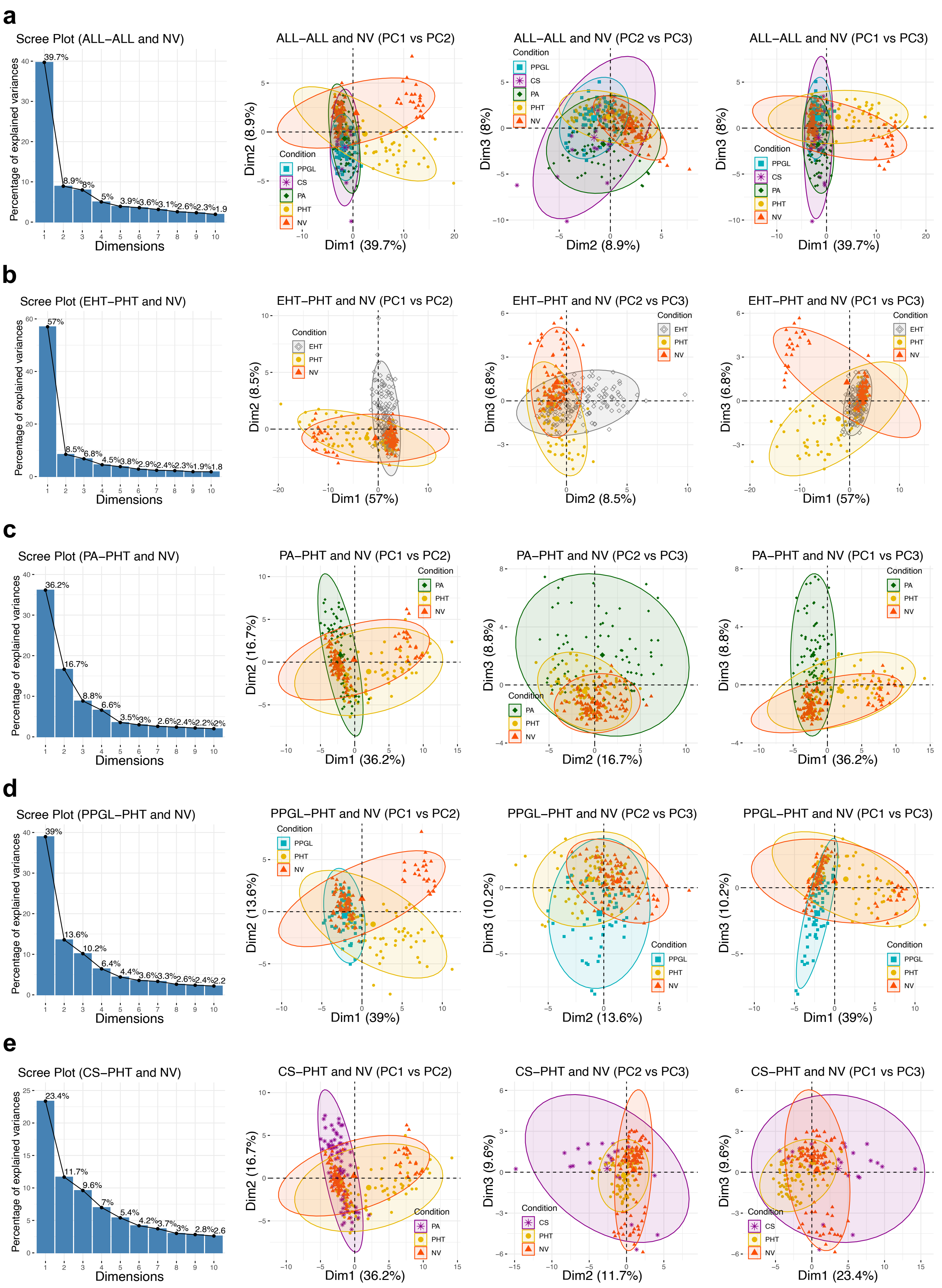

Supplementary Figure 5. Principal component analysis using top features of training data for (a) ALL-ALL, (b) EHT-PHT, (c) PA-PHT, (d) PPGL-PHT and (e) CS-PHT disease combination along with NV samples. The percentage of variation explained by each PC axis is given within parenthesis. The large dots represent the cluster centroid of each condition.

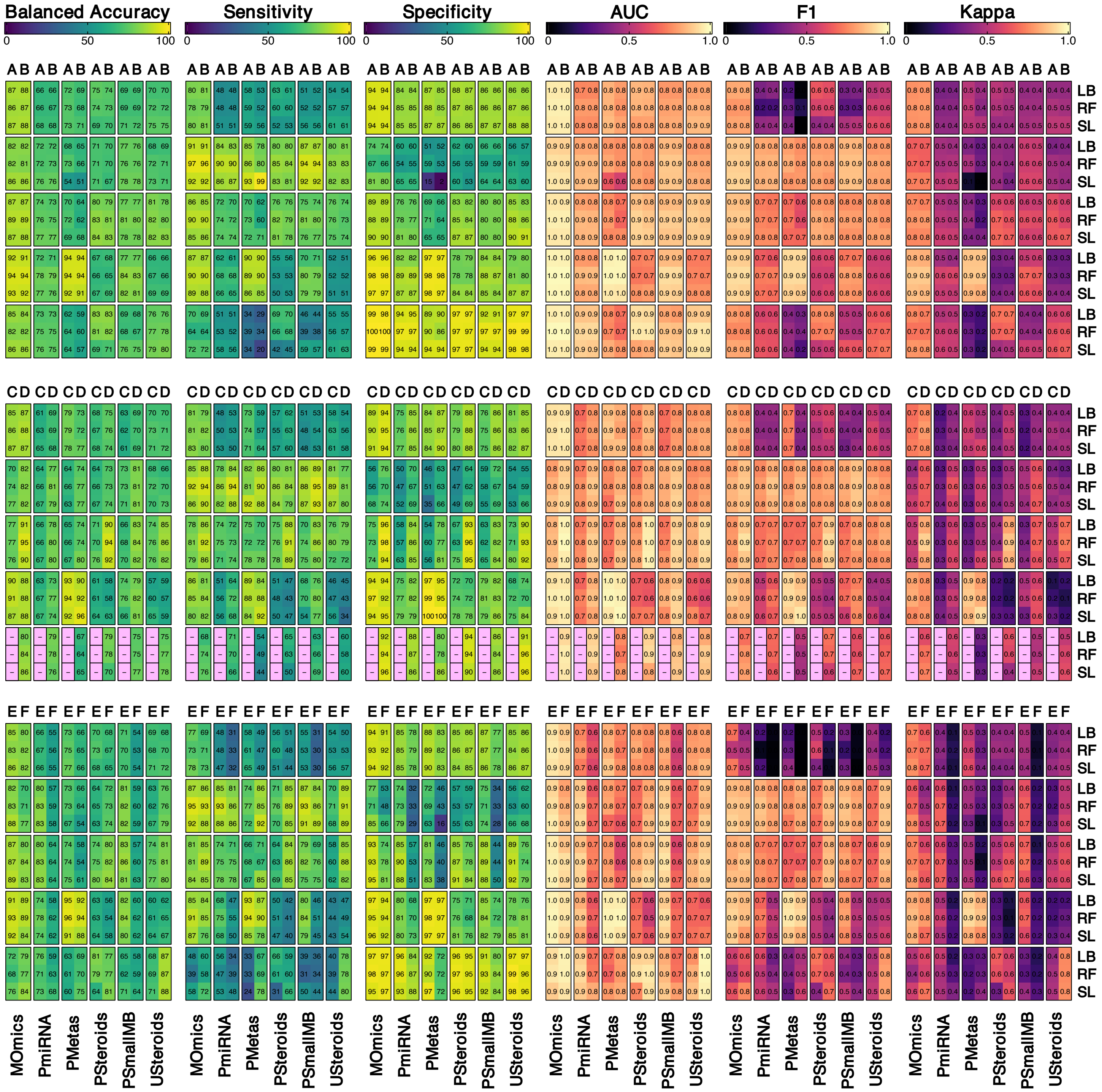

Supplementary Figure 6. Heatmap showing the mean classification performance metrics (over 100 random repeats) using multi-omics and 5 individual omics with 3 best classifiers for 5 disease combinations in Scenario 1 (Set A & B), 2 (Set C & D) and 3 (Set E & F).

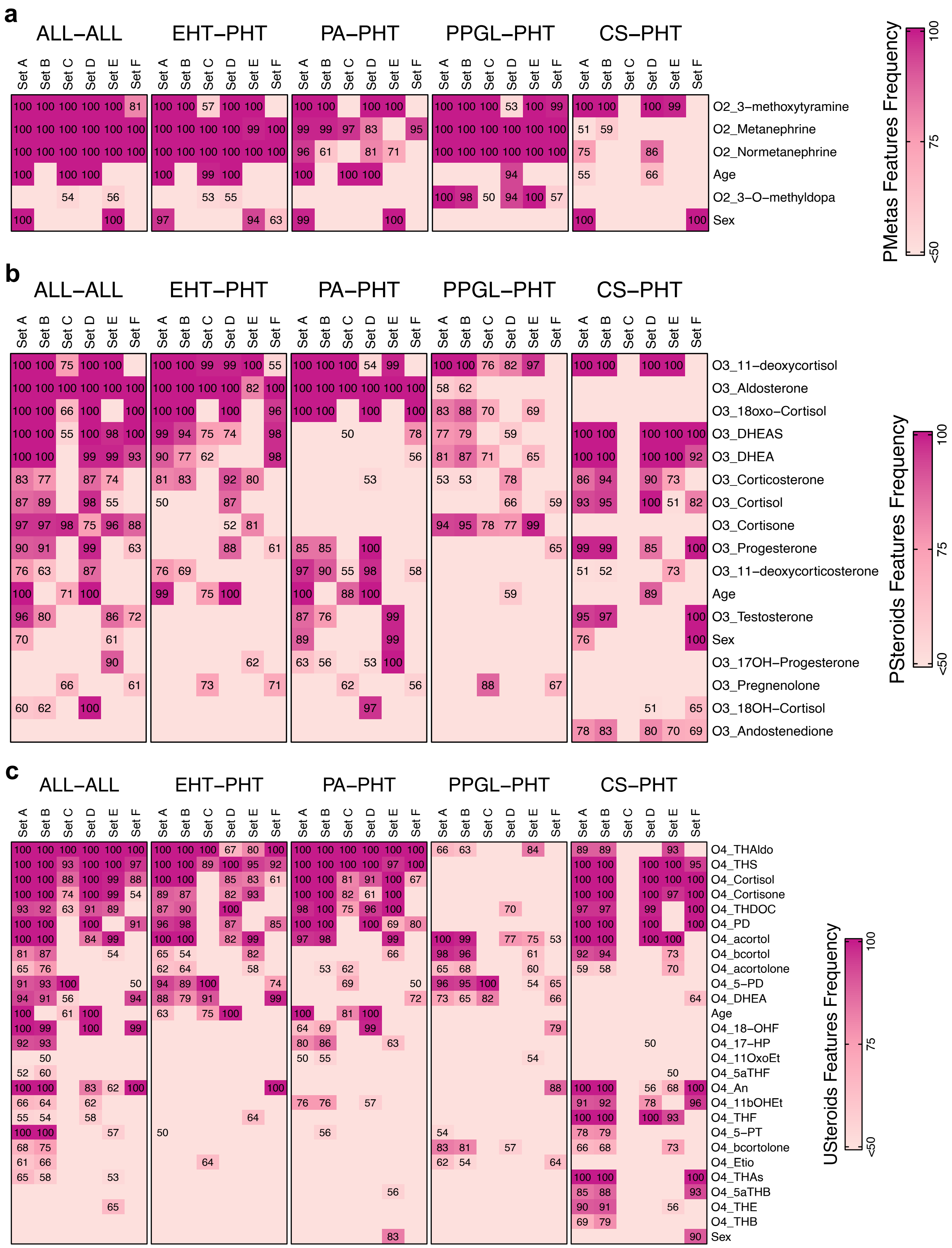

Supplementary Figure 7. Joint heatmap for 5 disease combinations showing the list of top features truncated with repeat frequency cutoff value of 50 (over 100 random repeats) selected during the classification using (a) PMetas, (b) PSteroids and (c) USteroids data for Set A - F.

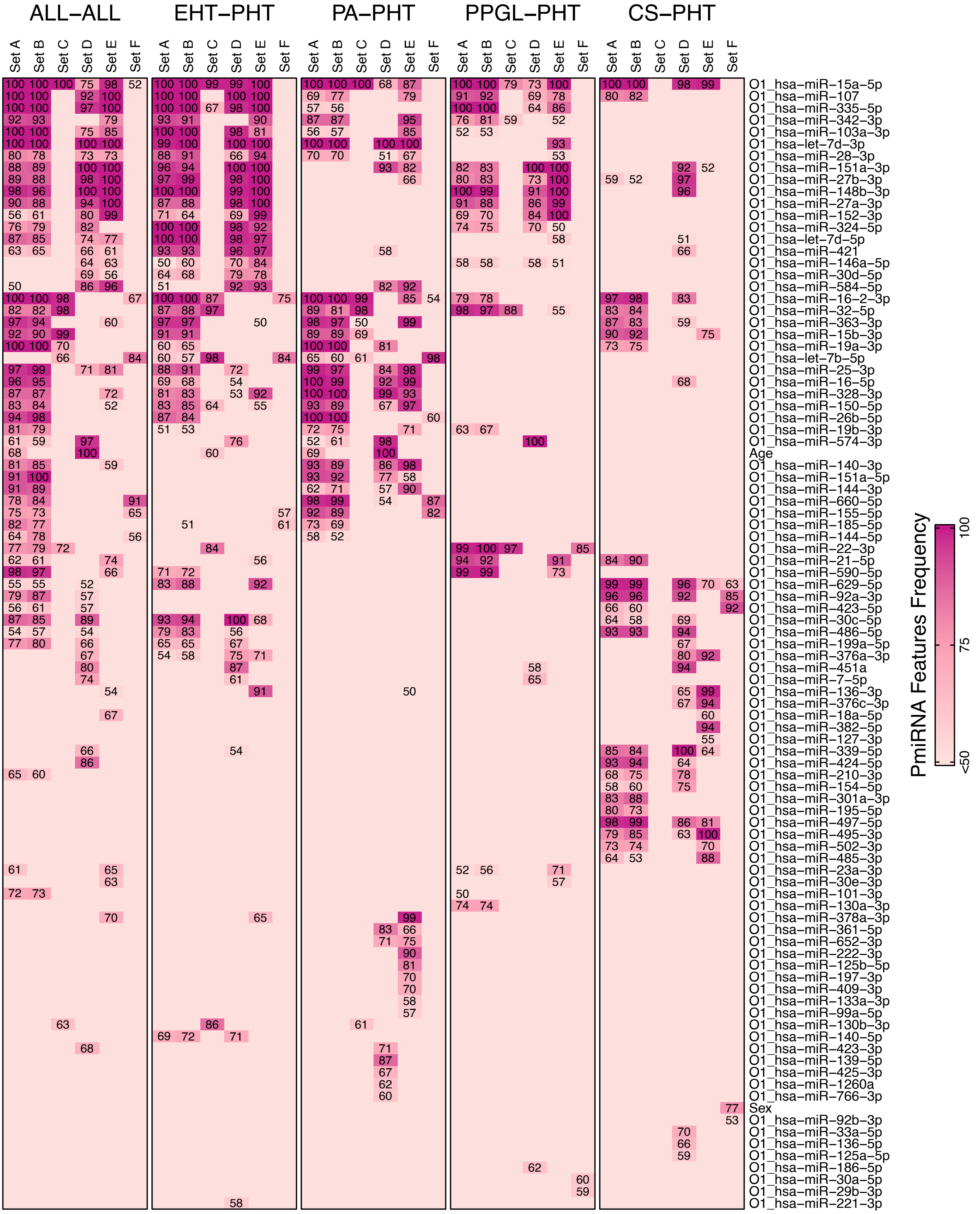

Supplementary Figure 8. Joint heatmap for 5 disease combinations showing the list of top features truncated with repeat frequency cutoff value of 50 (over 100 random repeats) selected during the classification using PmiRNA data for Set A - F.

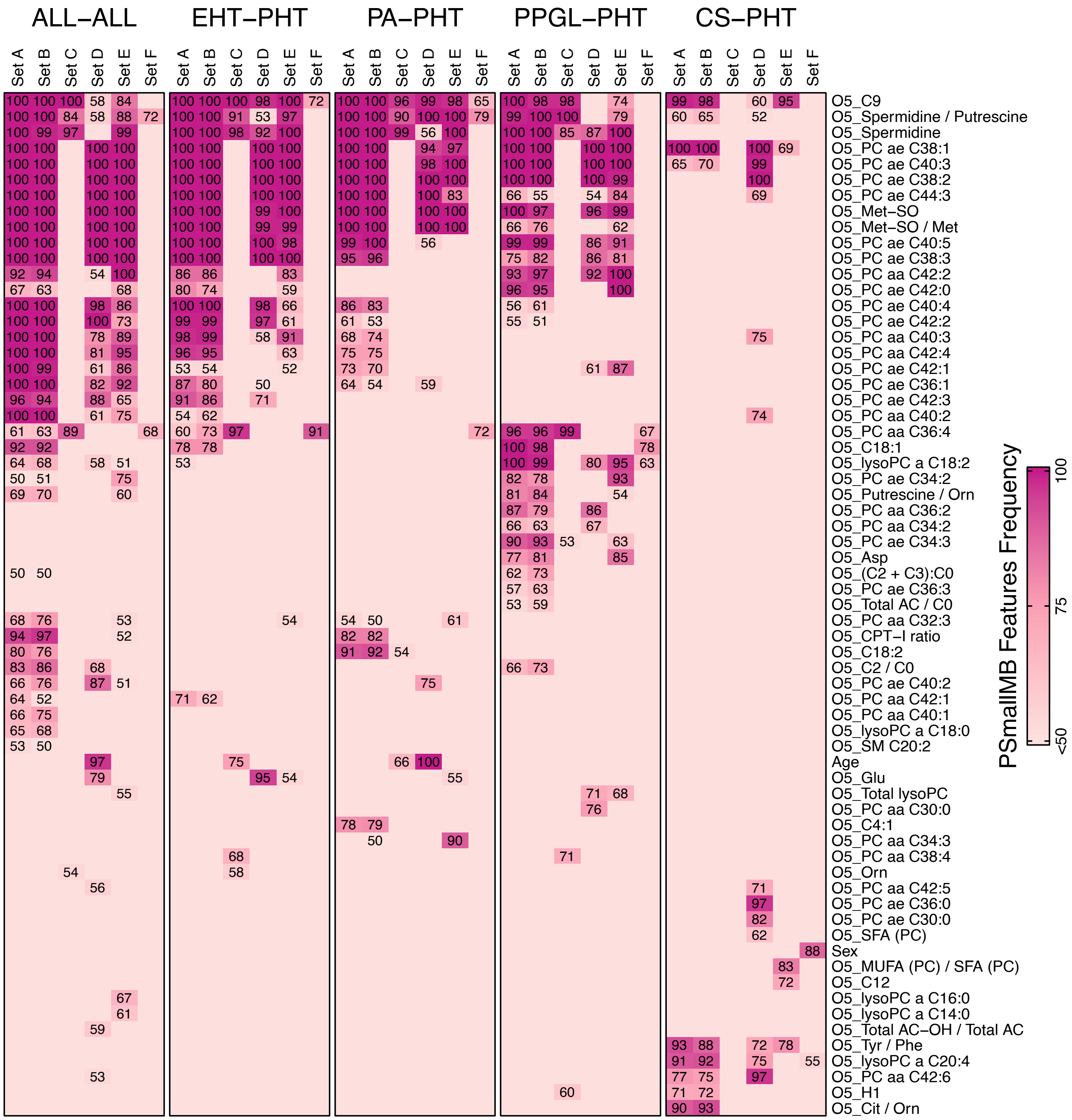

Supplementary Figure 9. Joint heatmap for 5 disease combinations showing the list of top features truncated with repeat frequency cutoff value of 50 (over 100 random repeats) selected during the classification using PSmallMB data for Set A - F.

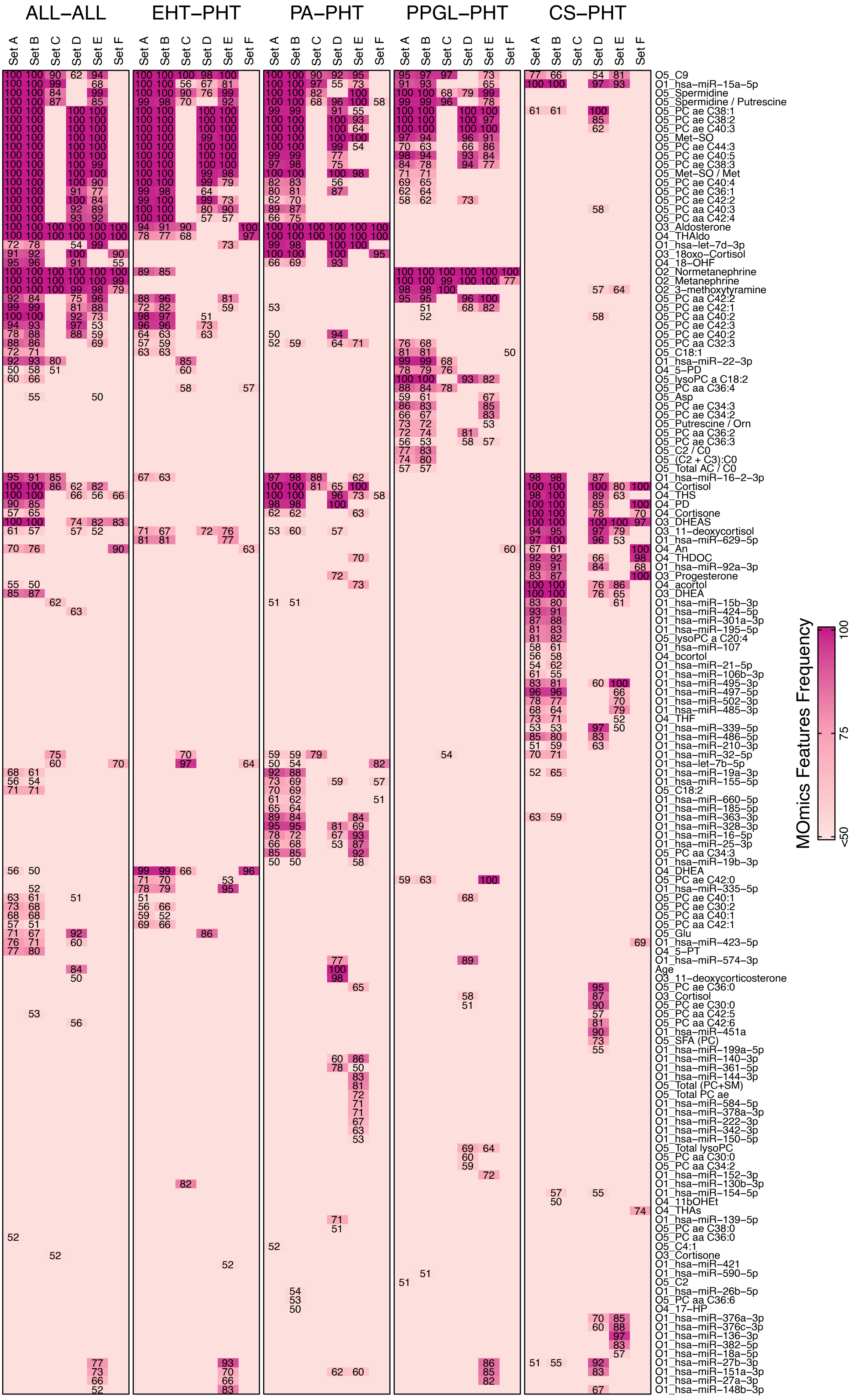

Supplementary Figure 10. Joint heatmap for 5 disease combinations showing the list of top features truncated with repeat frequency cutoff value of 50 (over 100 random repeats) selected during the classification using MOmics data for Set A - F.
