# Supplementary material for: Machine learning for classification of hypertension subtypes using multi-omics: A multi-centre, retrospective, data-driven study
Source: eBioMedicine. 2022 Sep 27;84:104276. doi: 10.1016/j.ebiom.2022.104276 (PMC9520210; doi:10.1016/j.ebiom.2022.104276)
Supplement: Supplementary file 2 [file mmc2.docx]

**Supplementary Table 1**: Sample material and volumes used for different omics measurements.

| **Type of omics** | **material** | **volume** |
| --- | --- | --- |
| PmiRNA | EDTA Plasma | 0.5 ml |
| PMetas | Lithium-Heparin Plasma | 0.6 mL |
| PSteroids | EDTA Plasma | 1 mL |
| USteroids | 24h Urines | 2 ml |
| PSmallMB | EDTA Plasma | 50µl |

**Supplementary Table 2**: List of multi-omics features and their distribution.

|  |  | **PA** | | **PPGL** | | **CS** | | **PHT** | | **NV** | |
| --- | --- | --- | --- | --- | --- | --- | --- | --- | --- | --- | --- |
| **Features** | **Omic**  **Type** | ***Mean*** | ***S.D*** | ***Mean*** | ***S.D*** | ***Mean*** | ***S.D*** | ***Mean*** | ***S.D*** | ***Mean*** | ***S.D*** |
| **O1_hsa-let-7a-5p** | PmiRNA | -1.16 | 0.79 | -1.34 | 0.75 | -1.25 | 1.33 | -1.55 | 0.68 | -1.17 | 0.79 |
| **O1_hsa-let-7b-3p** | PmiRNA | 5.42 | 0.97 | 5.54 | 0.88 | 5.06 | 1.16 | 5.19 | 0.78 | 5.30 | 1.18 |
| **O1_hsa-let-7b-5p** | PmiRNA | 0.85 | 0.59 | 0.77 | 0.63 | 0.60 | 0.84 | 0.71 | 0.80 | -0.18 | 0.67 |
| **O1_hsa-let-7c-5p** | PmiRNA | 3.69 | 1.02 | 3.34 | 0.75 | 3.41 | 1.20 | 3.22 | 0.72 | 3.59 | 0.74 |
| **O1_hsa-let-7d-3p** | PmiRNA | 1.13 | 0.63 | 1.01 | 0.63 | 0.89 | 0.69 | 0.55 | 0.75 | 1.23 | 0.75 |
| **O1_hsa-let-7d-5p** | PmiRNA | 2.20 | 1.00 | 1.84 | 0.83 | 2.26 | 1.16 | 2.07 | 0.72 | 2.24 | 0.85 |
| **O1_hsa-let-7e-5p** | PmiRNA | 4.49 | 1.45 | 4.20 | 1.12 | 4.17 | 1.68 | 4.02 | 1.00 | 4.48 | 1.43 |
| **O1_hsa-let-7f-5p** | PmiRNA | 1.04 | 1.07 | 0.89 | 0.86 | 1.00 | 1.39 | 0.76 | 0.81 | 1.30 | 0.84 |
| **O1_hsa-miR-1** | PmiRNA | 7.33 | 2.68 | 6.62 | 1.85 | 8.51 | 3.75 | 6.73 | 2.18 | 7.72 | 2.50 |
| **O1_hsa-miR-100-5p** | PmiRNA | 6.09 | 1.68 | 6.02 | 1.62 | 6.23 | 2.35 | 5.76 | 1.45 | 4.86 | 1.58 |
| **O1_hsa-miR-101-3p** | PmiRNA | 0.01 | 0.68 | 0.33 | 0.72 | 0.15 | 0.74 | 0.31 | 0.52 | -0.16 | 0.43 |
| **O1_hsa-miR-103a-3p** | PmiRNA | -1.15 | 0.99 | -1.53 | 0.81 | -1.05 | 1.00 | -1.24 | 0.68 | -1.09 | 0.80 |
| **O1_hsa-miR-106b-3p** | PmiRNA | 5.70 | 1.42 | 5.67 | 1.64 | 6.59 | 2.52 | 5.24 | 1.26 | 6.38 | 1.66 |
| **O1_hsa-miR-106b-5p** | PmiRNA | 1.18 | 0.43 | 1.19 | 0.43 | 1.53 | 0.47 | 1.30 | 0.42 | 0.93 | 0.36 |
| **O1_hsa-miR-107** | PmiRNA | 1.40 | 0.97 | 0.97 | 0.82 | 1.42 | 1.05 | 1.41 | 0.73 | 1.59 | 0.87 |
| **O1_hsa-miR-10b-5p** | PmiRNA | 5.26 | 1.58 | 5.28 | 1.68 | 4.55 | 1.52 | 5.05 | 1.25 | 4.85 | 1.36 |
| **O1_hsa-miR-122-5p** | PmiRNA | 0.74 | 1.84 | 0.82 | 2.24 | 0.33 | 2.05 | 0.82 | 2.07 | -0.51 | 1.53 |
| **O1_hsa-miR-125a-5p** | PmiRNA | 2.64 | 1.04 | 2.35 | 0.91 | 2.03 | 1.34 | 2.35 | 0.85 | 2.54 | 1.20 |
| **O1_hsa-miR-125b-5p** | PmiRNA | 2.88 | 0.94 | 3.00 | 1.02 | 2.69 | 1.21 | 2.62 | 0.96 | 1.84 | 0.70 |
| **O1_hsa-miR-1260a** | PmiRNA | 3.82 | 1.42 | 3.53 | 1.47 | 2.29 | 2.71 | 3.58 | 1.26 | 3.13 | 1.14 |
| **O1_hsa-miR-126-3p** | PmiRNA | -2.51 | 0.72 | -2.59 | 0.59 | -2.53 | 0.93 | -2.76 | 0.54 | -2.22 | 0.56 |
| **O1_hsa-miR-126-5p** | PmiRNA | 2.15 | 1.89 | 2.15 | 0.70 | 2.13 | 1.37 | 2.03 | 0.67 | 2.78 | 0.52 |
| **O1_hsa-miR-127-3p** | PmiRNA | 5.63 | 2.07 | 5.27 | 1.89 | 6.87 | 3.05 | 4.98 | 2.03 | 5.95 | 2.58 |
| **O1_hsa-miR-128-3p** | PmiRNA | 4.84 | 1.08 | 4.76 | 1.02 | 5.17 | 1.62 | 4.39 | 0.80 | 5.18 | 1.16 |
| **O1_hsa-miR-130a-3p** | PmiRNA | 3.50 | 0.78 | 2.98 | 0.94 | 3.75 | 0.75 | 3.18 | 1.06 | 4.35 | 0.77 |
| **O1_hsa-miR-130b-3p** | PmiRNA | 6.24 | 0.83 | 5.92 | 1.01 | 6.33 | 1.48 | 6.03 | 1.00 | 6.97 | 1.08 |
| **O1_hsa-miR-132-3p** | PmiRNA | 4.63 | 0.64 | 4.86 | 0.78 | 4.84 | 1.12 | 4.50 | 0.59 | 4.90 | 1.08 |
| **O1_hsa-miR-133a-3p** | PmiRNA | 5.95 | 1.80 | 5.04 | 1.59 | 5.92 | 2.46 | 5.13 | 1.28 | 6.22 | 1.73 |
| **O1_hsa-miR-133b** | PmiRNA | 4.93 | 1.42 | 4.44 | 1.83 | 5.38 | 2.60 | 4.40 | 1.36 | 5.17 | 1.54 |
| **O1_hsa-miR-136-3p** | PmiRNA | 7.99 | 3.06 | 7.15 | 2.53 | 9.16 | 3.47 | 6.86 | 2.68 | 9.65 | 3.19 |
| **O1_hsa-miR-136-5p** | PmiRNA | 7.17 | 3.95 | 6.04 | 3.45 | 8.45 | 4.34 | 6.06 | 3.15 | 7.29 | 3.47 |
| **O1_hsa-miR-139-5p** | PmiRNA | 3.13 | 0.96 | 2.69 | 0.81 | 2.90 | 1.30 | 2.77 | 0.80 | 3.11 | 0.87 |
| **O1_hsa-miR-140-3p** | PmiRNA | 0.55 | 1.05 | 0.79 | 0.86 | 0.39 | 0.89 | 1.39 | 1.74 | 0.50 | 0.52 |
| **O1_hsa-miR-140-5p** | PmiRNA | 3.67 | 0.65 | 3.57 | 0.58 | 3.74 | 0.97 | 3.83 | 0.58 | 4.34 | 0.76 |
| **O1_hsa-miR-141-3p** | PmiRNA | 7.75 | 1.76 | 7.52 | 1.53 | 8.66 | 2.42 | 7.59 | 1.61 | 7.99 | 2.32 |
| **O1_hsa-miR-142-3p** | PmiRNA | -1.03 | 0.88 | -1.14 | 0.74 | -0.98 | 1.10 | -0.87 | 0.87 | -0.91 | 0.88 |
| **O1_hsa-miR-142-5p** | PmiRNA | 2.27 | 0.93 | 2.04 | 0.97 | 2.66 | 0.97 | 1.99 | 0.90 | 3.08 | 0.84 |
| **O1_hsa-miR-143-3p** | PmiRNA | 3.25 | 1.09 | 3.24 | 1.06 | 3.38 | 1.31 | 2.94 | 1.02 | 2.94 | 1.03 |
| **O1_hsa-miR-144-3p** | PmiRNA | 0.65 | 1.98 | 0.83 | 1.63 | 0.43 | 1.37 | 1.54 | 1.52 | -0.52 | 0.66 |
| **O1_hsa-miR-144-5p** | PmiRNA | 4.31 | 1.38 | 4.85 | 1.43 | 4.21 | 1.62 | 4.90 | 1.14 | 4.29 | 1.26 |
| **O1_hsa-miR-145-5p** | PmiRNA | 2.74 | 1.04 | 2.57 | 0.89 | 2.07 | 0.99 | 2.23 | 0.90 | 2.34 | 0.84 |
| **O1_hsa-miR-146a-5p** | PmiRNA | 1.50 | 1.18 | 1.32 | 0.98 | 1.76 | 1.13 | 0.83 | 1.19 | 2.19 | 0.92 |
| **O1_hsa-miR-146b-5p** | PmiRNA | 6.91 | 0.94 | 6.79 | 0.74 | 7.01 | 1.48 | 6.73 | 0.83 | 7.39 | 1.32 |
| **O1_hsa-miR-148a-3p** | PmiRNA | 2.19 | 0.57 | 2.18 | 0.68 | 2.06 | 0.77 | 2.13 | 0.76 | 2.55 | 1.05 |
| **O1_hsa-miR-148b-3p** | PmiRNA | 1.52 | 0.53 | 1.46 | 0.44 | 1.77 | 0.45 | 1.25 | 0.83 | 1.99 | 0.70 |
| **O1_hsa-miR-150-5p** | PmiRNA | 0.40 | 1.48 | 0.27 | 1.13 | -0.39 | 1.33 | -0.04 | 1.29 | -0.57 | 0.97 |
| **O1_hsa-miR-151a-3p** | PmiRNA | 2.93 | 0.99 | 2.92 | 1.05 | 3.27 | 1.29 | 2.32 | 0.99 | 3.47 | 0.71 |
| **O1_hsa-miR-151a-5p** | PmiRNA | 0.86 | 1.15 | 0.63 | 0.82 | 0.87 | 1.24 | 0.40 | 0.72 | 1.06 | 0.68 |
| **O1_hsa-miR-152-3p** | PmiRNA | 3.71 | 0.92 | 3.65 | 0.80 | 4.39 | 1.84 | 3.17 | 1.00 | 4.08 | 1.23 |
| **O1_hsa-miR-154-5p** | PmiRNA | 5.94 | 2.49 | 5.58 | 2.33 | 7.07 | 3.28 | 4.87 | 2.30 | 6.78 | 2.72 |
| **O1_hsa-miR-155-5p** | PmiRNA | 5.11 | 0.95 | 4.82 | 0.84 | 4.78 | 1.38 | 4.82 | 1.05 | 5.11 | 1.46 |
| **O1_hsa-miR-15a-5p** | PmiRNA | -1.31 | 0.81 | -1.21 | 0.69 | -1.45 | 0.86 | -0.66 | 0.50 | -1.08 | 0.46 |
| **O1_hsa-miR-15b-3p** | PmiRNA | 2.87 | 0.51 | 3.15 | 0.56 | 2.68 | 0.87 | 3.24 | 0.40 | 3.19 | 0.55 |
| **O1_hsa-miR-15b-5p** | PmiRNA | 1.89 | 0.65 | 1.96 | 0.51 | 1.93 | 0.62 | 1.79 | 0.68 | 2.46 | 0.56 |
| **O1_hsa-miR-16-2-3p** | PmiRNA | 3.74 | 1.19 | 3.98 | 1.00 | 3.45 | 1.09 | 4.44 | 0.76 | 3.85 | 0.75 |
| **O1_hsa-miR-16-5p** | PmiRNA | -6.22 | 0.94 | -5.95 | 0.88 | -6.24 | 0.89 | -5.49 | 0.79 | -6.19 | 0.60 |
| **O1_hsa-miR-17-5p** | PmiRNA | 4.46 | 0.52 | 4.52 | 0.66 | 4.70 | 1.22 | 4.36 | 0.42 | 4.57 | 0.80 |
| **O1_hsa-miR-181a-5p** | PmiRNA | 1.65 | 0.89 | 1.48 | 0.80 | 1.69 | 1.27 | 1.01 | 0.78 | 1.33 | 0.92 |
| **O1_hsa-miR-185-5p** | PmiRNA | -2.28 | 0.46 | -2.04 | 0.45 | -2.38 | 0.57 | -1.99 | 0.32 | -2.25 | 0.47 |
| **O1_hsa-miR-186-5p** | PmiRNA | 5.75 | 0.84 | 5.99 | 1.14 | 6.25 | 1.54 | 5.65 | 0.80 | 5.72 | 1.05 |
| **O1_hsa-miR-18a-5p** | PmiRNA | 1.03 | 0.55 | 1.06 | 0.60 | 1.38 | 0.72 | 0.89 | 0.42 | 1.23 | 0.43 |
| **O1_hsa-miR-18b-5p** | PmiRNA | 1.34 | 0.48 | 1.40 | 0.49 | 1.51 | 0.44 | 1.14 | 0.36 | 1.47 | 0.35 |
| **O1_hsa-miR-191-5p** | PmiRNA | -0.15 | 0.95 | -0.50 | 0.91 | -0.23 | 1.00 | -0.57 | 0.70 | 0.03 | 0.62 |
| **O1_hsa-miR-192-5p** | PmiRNA | 2.57 | 1.08 | 2.82 | 1.17 | 2.64 | 1.28 | 3.14 | 1.22 | 2.18 | 0.72 |
| **O1_hsa-miR-193a-5p** | PmiRNA | 7.01 | 1.66 | 7.18 | 1.45 | 6.43 | 1.67 | 7.10 | 1.49 | 6.38 | 1.22 |
| **O1_hsa-miR-194-5p** | PmiRNA | 4.60 | 1.09 | 4.91 | 1.27 | 4.40 | 0.99 | 5.15 | 1.09 | 4.19 | 0.72 |
| **O1_hsa-miR-195-5p** | PmiRNA | 6.58 | 1.22 | 6.54 | 1.20 | 7.66 | 2.53 | 6.28 | 1.09 | 6.78 | 1.80 |
| **O1_hsa-miR-197-3p** | PmiRNA | 2.29 | 0.94 | 2.17 | 0.82 | 1.92 | 1.04 | 1.74 | 0.73 | 2.22 | 0.80 |
| **O1_hsa-miR-199a-3p** | PmiRNA | 1.74 | 1.29 | 1.48 | 1.15 | 2.12 | 1.17 | 1.24 | 1.15 | 2.43 | 0.92 |
| **O1_hsa-miR-199a-5p** | PmiRNA | 3.20 | 1.77 | 2.54 | 1.69 | 3.97 | 2.38 | 2.16 | 1.46 | 3.48 | 1.50 |
| **O1_hsa-miR-19a-3p** | PmiRNA | -2.32 | 0.55 | -1.98 | 0.67 | -2.12 | 0.82 | -1.85 | 0.42 | -1.84 | 0.68 |
| **O1_hsa-miR-19b-3p** | PmiRNA | -3.44 | 0.70 | -3.21 | 0.72 | -3.33 | 0.78 | -3.04 | 0.42 | -3.11 | 0.58 |
| **O1_hsa-miR-200a-3p** | PmiRNA | 10.56 | 3.50 | 10.94 | 3.64 | 12.21 | 3.98 | 9.88 | 3.37 | 12.02 | 4.31 |
| **O1_hsa-miR-200c-3p** | PmiRNA | 7.37 | 2.18 | 6.94 | 2.03 | 7.86 | 2.89 | 6.79 | 1.52 | 7.97 | 2.54 |
| **O1_hsa-miR-205-5p** | PmiRNA | 8.58 | 2.35 | 8.53 | 2.02 | 9.28 | 3.07 | 8.40 | 2.06 | 6.76 | 1.86 |
| **O1_hsa-miR-20a-5p** | PmiRNA | -1.92 | 0.28 | -1.79 | 0.34 | -1.87 | 0.44 | -1.78 | 0.25 | -1.83 | 0.27 |
| **O1_hsa-miR-20b-5p** | PmiRNA | 6.83 | 1.42 | 6.99 | 1.22 | 6.54 | 1.54 | 7.65 | 1.42 | 6.73 | 1.59 |
| **O1_hsa-miR-210-3p** | PmiRNA | 3.64 | 1.02 | 3.66 | 0.84 | 3.88 | 2.00 | 4.06 | 0.68 | 4.12 | 1.39 |
| **O1_hsa-miR-2110** | PmiRNA | 6.15 | 0.74 | 6.20 | 0.82 | 5.66 | 1.01 | 6.21 | 0.81 | 6.03 | 1.13 |
| **O1_hsa-miR-215-5p** | PmiRNA | 3.74 | 1.07 | 4.04 | 1.26 | 3.80 | 1.21 | 4.30 | 1.24 | 3.34 | 0.88 |
| **O1_hsa-miR-21-5p** | PmiRNA | -2.34 | 0.45 | -2.16 | 0.46 | -2.10 | 0.64 | -2.53 | 0.55 | -2.04 | 0.60 |
| **O1_hsa-miR-221-3p** | PmiRNA | -0.21 | 1.38 | -0.69 | 1.15 | 0.09 | 1.44 | -0.91 | 1.30 | 0.33 | 0.97 |
| **O1_hsa-miR-222-3p** | PmiRNA | -4.89 | 0.97 | -4.99 | 0.87 | -5.07 | 0.98 | -5.31 | 0.82 | -4.35 | 0.71 |
| **O1_hsa-miR-223-5p** | PmiRNA | 5.98 | 0.87 | 6.31 | 1.51 | 6.58 | 2.65 | 5.71 | 0.87 | 6.75 | 1.84 |
| **O1_hsa-miR-22-3p** | PmiRNA | 1.92 | 0.60 | 1.69 | 0.66 | 2.17 | 0.95 | 2.00 | 0.68 | 2.79 | 0.48 |
| **O1_hsa-miR-22-5p** | PmiRNA | 5.87 | 1.24 | 5.85 | 1.67 | 6.33 | 1.75 | 5.87 | 1.22 | 7.07 | 1.81 |
| **O1_hsa-miR-23a-3p** | PmiRNA | -2.71 | 0.91 | -2.75 | 0.78 | -2.58 | 0.89 | -3.17 | 0.73 | -2.30 | 0.71 |
| **O1_hsa-miR-23b-3p** | PmiRNA | -0.77 | 0.95 | -0.83 | 0.78 | -0.83 | 0.97 | -1.18 | 0.73 | -0.39 | 0.73 |
| **O1_hsa-miR-24-3p** | PmiRNA | -2.02 | 0.88 | -2.17 | 0.76 | -1.97 | 0.85 | -2.48 | 0.73 | -1.51 | 0.63 |
| **O1_hsa-miR-25-3p** | PmiRNA | -1.91 | 0.91 | -1.63 | 0.79 | -2.15 | 0.87 | -1.26 | 0.75 | -1.94 | 0.51 |
| **O1_hsa-miR-26a-5p** | PmiRNA | 1.47 | 1.12 | 1.25 | 0.86 | 1.54 | 1.22 | 1.34 | 0.79 | 1.71 | 0.74 |
| **O1_hsa-miR-26b-5p** | PmiRNA | 2.56 | 0.71 | 2.59 | 0.63 | 2.91 | 0.88 | 2.59 | 0.56 | 2.53 | 0.68 |
| **O1_hsa-miR-27a-3p** | PmiRNA | 1.57 | 1.00 | 1.50 | 0.79 | 1.89 | 0.96 | 0.98 | 0.90 | 1.91 | 0.72 |
| **O1_hsa-miR-27b-3p** | PmiRNA | 0.09 | 0.98 | 0.04 | 0.87 | 0.41 | 0.87 | -0.53 | 0.80 | 0.36 | 0.73 |
| **O1_hsa-miR-28-3p** | PmiRNA | 3.90 | 1.14 | 3.72 | 0.96 | 3.83 | 1.49 | 3.13 | 0.91 | 4.04 | 0.90 |
| **O1_hsa-miR-28-5p** | PmiRNA | 3.44 | 1.33 | 3.68 | 2.01 | 4.00 | 2.29 | 3.17 | 1.00 | 4.12 | 1.55 |
| **O1_hsa-miR-29a-3p** | PmiRNA | 3.68 | 0.77 | 3.60 | 0.70 | 3.72 | 0.92 | 3.57 | 0.73 | 3.73 | 0.74 |
| **O1_hsa-miR-29b-3p** | PmiRNA | 4.27 | 0.60 | 4.15 | 0.59 | 4.42 | 0.75 | 4.46 | 0.55 | 4.63 | 0.63 |
| **O1_hsa-miR-29c-3p** | PmiRNA | 3.28 | 1.04 | 3.19 | 0.73 | 3.42 | 1.04 | 3.58 | 0.69 | 3.47 | 0.57 |
| **O1_hsa-miR-301a-3p** | PmiRNA | 3.27 | 1.02 | 3.24 | 0.95 | 3.95 | 1.70 | 3.02 | 0.67 | 3.33 | 0.83 |
| **O1_hsa-miR-30a-5p** | PmiRNA | 4.76 | 1.06 | 4.96 | 1.17 | 4.92 | 1.19 | 4.59 | 0.92 | 4.52 | 1.05 |
| **O1_hsa-miR-30b-5p** | PmiRNA | 0.27 | 0.85 | 0.18 | 0.73 | 0.35 | 0.97 | 0.03 | 0.60 | 0.40 | 0.61 |
| **O1_hsa-miR-30c-5p** | PmiRNA | 0.64 | 0.79 | 0.59 | 0.68 | 0.28 | 0.97 | 0.84 | 0.78 | 1.03 | 0.76 |
| **O1_hsa-miR-30d-5p** | PmiRNA | -0.66 | 0.59 | -0.72 | 0.48 | -0.76 | 0.56 | -1.10 | 0.61 | -0.55 | 0.48 |
| **O1_hsa-miR-30e-3p** | PmiRNA | 6.16 | 1.11 | 6.06 | 0.89 | 5.81 | 1.00 | 5.78 | 0.99 | 6.41 | 1.28 |
| **O1_hsa-miR-30e-5p** | PmiRNA | 0.17 | 1.08 | 0.44 | 1.17 | 0.55 | 1.33 | 0.42 | 1.06 | 0.61 | 1.07 |
| **O1_hsa-miR-320a** | PmiRNA | -0.99 | 0.47 | -0.93 | 0.51 | -1.22 | 0.74 | -1.11 | 0.50 | -1.11 | 0.51 |
| **O1_hsa-miR-320b** | PmiRNA | 1.56 | 0.51 | 1.60 | 0.57 | 1.27 | 0.70 | 1.41 | 0.55 | 1.44 | 0.56 |
| **O1_hsa-miR-320c** | PmiRNA | 2.68 | 0.49 | 2.72 | 0.53 | 2.32 | 0.76 | 2.62 | 0.53 | 2.67 | 0.55 |
| **O1_hsa-miR-320d** | PmiRNA | 3.78 | 0.57 | 3.81 | 0.63 | 3.49 | 0.90 | 3.64 | 0.55 | 3.73 | 0.61 |
| **O1_hsa-miR-324-3p** | PmiRNA | 3.31 | 0.45 | 3.20 | 0.46 | 3.41 | 0.87 | 3.39 | 0.41 | 3.29 | 0.78 |
| **O1_hsa-miR-324-5p** | PmiRNA | 3.42 | 0.74 | 3.26 | 1.05 | 3.80 | 1.34 | 3.48 | 0.67 | 3.88 | 1.28 |
| **O1_hsa-miR-32-5p** | PmiRNA | 4.26 | 1.22 | 4.27 | 1.34 | 3.81 | 5.15 | 4.51 | 0.63 | 4.29 | 0.78 |
| **O1_hsa-miR-326** | PmiRNA | 6.28 | 2.14 | 5.86 | 2.65 | 5.72 | 2.24 | 5.30 | 2.31 | 6.63 | 2.70 |
| **O1_hsa-miR-328-3p** | PmiRNA | 2.37 | 1.15 | 2.10 | 0.88 | 2.28 | 1.01 | 1.63 | 0.86 | 2.38 | 0.80 |
| **O1_hsa-miR-331-3p** | PmiRNA | 3.04 | 0.92 | 2.83 | 0.92 | 3.00 | 0.97 | 2.65 | 0.82 | 3.16 | 0.63 |
| **O1_hsa-miR-335-3p** | PmiRNA | 6.03 | 2.31 | 5.42 | 1.85 | 6.20 | 3.19 | 5.27 | 2.35 | 6.40 | 2.34 |
| **O1_hsa-miR-335-5p** | PmiRNA | 6.48 | 1.13 | 6.47 | 1.01 | 7.00 | 1.60 | 5.84 | 1.39 | 7.31 | 1.22 |
| **O1_hsa-miR-338-3p** | PmiRNA | 4.46 | 1.15 | 4.18 | 1.05 | 4.47 | 1.22 | 3.99 | 1.06 | 5.13 | 1.32 |
| **O1_hsa-miR-339-3p** | PmiRNA | 5.35 | 1.46 | 5.03 | 1.15 | 5.52 | 1.76 | 4.62 | 1.01 | 5.77 | 1.72 |
| **O1_hsa-miR-339-5p** | PmiRNA | 2.53 | 1.15 | 2.01 | 0.98 | 2.28 | 1.43 | 2.12 | 0.86 | 3.00 | 1.51 |
| **O1_hsa-miR-33a-5p** | PmiRNA | 5.88 | 1.93 | 5.06 | 1.68 | 6.74 | 2.50 | 4.98 | 1.36 | 6.49 | 1.76 |
| **O1_hsa-miR-342-3p** | PmiRNA | 1.94 | 0.77 | 1.96 | 0.73 | 1.52 | 0.97 | 1.48 | 0.71 | 1.35 | 0.87 |
| **O1_hsa-miR-34a-5p** | PmiRNA | 8.05 | 2.60 | 7.53 | 2.81 | 9.30 | 3.83 | 7.90 | 2.63 | 8.50 | 3.63 |
| **O1_hsa-miR-361-5p** | PmiRNA | 1.90 | 0.71 | 1.83 | 0.68 | 1.79 | 0.72 | 1.44 | 0.67 | 2.28 | 0.72 |
| **O1_hsa-miR-362-3p** | PmiRNA | 5.60 | 1.37 | 6.03 | 1.50 | 6.07 | 1.89 | 6.02 | 1.36 | 6.33 | 2.03 |
| **O1_hsa-miR-363-3p** | PmiRNA | 2.86 | 1.20 | 2.93 | 0.92 | 2.69 | 1.17 | 3.56 | 0.82 | 2.76 | 0.57 |
| **O1_hsa-miR-365a-3p** | PmiRNA | 6.19 | 1.51 | 6.23 | 2.15 | 6.27 | 2.61 | 6.48 | 1.74 | 6.12 | 2.12 |
| **O1_hsa-miR-374a-5p** | PmiRNA | 4.47 | 2.31 | 4.44 | 1.97 | 4.09 | 4.49 | 4.37 | 2.24 | 4.91 | 1.05 |
| **O1_hsa-miR-374b-5p** | PmiRNA | 3.56 | 1.39 | 3.39 | 0.96 | 3.99 | 1.58 | 3.41 | 0.91 | 4.22 | 1.13 |
| **O1_hsa-miR-375** | PmiRNA | 5.71 | 1.70 | 5.70 | 2.04 | 4.99 | 2.06 | 5.54 | 1.61 | 4.33 | 1.31 |
| **O1_hsa-miR-376a-3p** | PmiRNA | 6.19 | 2.20 | 5.96 | 1.92 | 6.86 | 1.90 | 5.18 | 1.75 | 6.74 | 1.97 |
| **O1_hsa-miR-376c-3p** | PmiRNA | 4.32 | 2.10 | 3.98 | 1.58 | 5.19 | 2.22 | 3.26 | 1.65 | 4.71 | 1.59 |
| **O1_hsa-miR-382-5p** | PmiRNA | 4.65 | 2.22 | 4.01 | 1.62 | 5.30 | 2.59 | 3.54 | 1.83 | 4.49 | 1.47 |
| **O1_hsa-miR-409-3p** | PmiRNA | 4.31 | 2.19 | 3.85 | 1.83 | 4.80 | 2.39 | 3.20 | 1.75 | 4.16 | 1.44 |
| **O1_hsa-miR-421** | PmiRNA | 5.53 | 0.89 | 5.56 | 1.07 | 6.15 | 1.81 | 5.00 | 1.10 | 6.10 | 1.46 |
| **O1_hsa-miR-423-3p** | PmiRNA | 1.28 | 0.81 | 0.89 | 0.89 | 1.34 | 1.17 | 0.82 | 0.80 | 1.47 | 0.64 |
| **O1_hsa-miR-423-5p** | PmiRNA | 1.18 | 0.52 | 0.97 | 0.65 | 0.55 | 0.71 | 1.13 | 0.48 | 1.09 | 0.60 |
| **O1_hsa-miR-424-5p** | PmiRNA | 4.54 | 1.01 | 4.61 | 0.84 | 3.96 | 1.31 | 4.83 | 0.89 | 4.59 | 0.91 |
| **O1_hsa-miR-425-3p** | PmiRNA | 4.31 | 0.65 | 4.21 | 0.79 | 4.44 | 1.28 | 4.03 | 0.87 | 4.76 | 0.81 |
| **O1_hsa-miR-451a** | PmiRNA | -6.02 | 1.66 | -5.95 | 1.58 | -6.62 | 1.44 | -5.02 | 1.54 | -6.67 | 0.62 |
| **O1_hsa-miR-454-3p** | PmiRNA | 4.57 | 1.47 | 4.59 | 2.37 | 4.35 | 1.93 | 5.02 | 1.44 | 4.08 | 2.16 |
| **O1_hsa-miR-483-5p** | PmiRNA | 7.70 | 1.83 | 7.35 | 2.10 | 7.24 | 2.90 | 7.30 | 1.72 | 6.48 | 1.60 |
| **O1_hsa-miR-484** | PmiRNA | 0.76 | 0.44 | 0.80 | 0.59 | 0.69 | 0.83 | 0.56 | 0.48 | 0.74 | 0.40 |
| **O1_hsa-miR-485-3p** | PmiRNA | 4.91 | 2.13 | 4.41 | 1.90 | 6.46 | 3.43 | 4.14 | 1.61 | 5.56 | 2.73 |
| **O1_hsa-miR-486-5p** | PmiRNA | -2.04 | 1.51 | -2.04 | 1.33 | -3.03 | 1.25 | -1.35 | 1.21 | -2.73 | 0.66 |
| **O1_hsa-miR-495-3p** | PmiRNA | 5.70 | 2.21 | 5.30 | 2.55 | 7.98 | 3.63 | 5.00 | 1.93 | 6.83 | 2.60 |
| **O1_hsa-miR-497-5p** | PmiRNA | 8.03 | 1.80 | 8.05 | 2.29 | 9.44 | 2.86 | 7.49 | 1.85 | 8.64 | 2.80 |
| **O1_hsa-miR-501-3p** | PmiRNA | 6.32 | 1.34 | 6.21 | 1.26 | 5.72 | 1.75 | 6.14 | 1.08 | 6.16 | 1.96 |
| **O1_hsa-miR-502-3p** | PmiRNA | 5.74 | 1.30 | 5.65 | 0.93 | 5.11 | 1.22 | 5.96 | 0.79 | 5.84 | 1.29 |
| **O1_hsa-miR-505-3p** | PmiRNA | 4.93 | 0.85 | 5.04 | 0.72 | 4.64 | 0.84 | 4.82 | 0.83 | 5.19 | 1.12 |
| **O1_hsa-miR-532-3p** | PmiRNA | 4.54 | 0.69 | 4.76 | 0.63 | 4.32 | 0.98 | 4.52 | 0.65 | 4.26 | 0.69 |
| **O1_hsa-miR-532-5p** | PmiRNA | 4.20 | 0.73 | 4.35 | 0.60 | 4.08 | 1.08 | 4.60 | 0.78 | 4.13 | 0.79 |
| **O1_hsa-miR-543** | PmiRNA | 5.90 | 2.39 | 5.14 | 2.12 | 6.22 | 2.61 | 4.94 | 1.97 | 6.36 | 2.35 |
| **O1_hsa-miR-574-3p** | PmiRNA | 3.50 | 1.13 | 3.37 | 1.23 | 2.01 | 4.25 | 2.93 | 0.65 | 3.45 | 0.87 |
| **O1_hsa-miR-584-5p** | PmiRNA | 5.58 | 1.25 | 5.29 | 1.00 | 5.13 | 1.00 | 4.79 | 1.07 | 5.60 | 0.87 |
| **O1_hsa-miR-590-5p** | PmiRNA | 3.23 | 0.53 | 3.49 | 0.62 | 3.62 | 1.05 | 3.12 | 0.68 | 3.54 | 0.64 |
| **O1_hsa-miR-629-5p** | PmiRNA | 6.00 | 1.27 | 5.86 | 1.00 | 5.13 | 1.41 | 6.58 | 1.50 | 6.29 | 2.10 |
| **O1_hsa-miR-652-3p** | PmiRNA | 1.99 | 0.81 | 1.70 | 0.77 | 2.14 | 0.86 | 1.45 | 0.65 | 1.98 | 0.55 |
| **O1_hsa-miR-660-5p** | PmiRNA | 2.62 | 1.07 | 2.84 | 0.91 | 2.51 | 0.90 | 3.12 | 0.76 | 2.56 | 0.54 |
| **O1_hsa-miR-7-1-3p** | PmiRNA | 6.34 | 1.46 | 6.21 | 1.46 | 6.46 | 2.63 | 5.76 | 0.81 | 6.82 | 1.67 |
| **O1_hsa-miR-7-5p** | PmiRNA | 6.18 | 1.27 | 6.31 | 1.21 | 5.85 | 1.42 | 6.81 | 1.18 | 5.40 | 0.99 |
| **O1_hsa-miR-766-3p** | PmiRNA | 3.64 | 1.37 | 3.20 | 1.01 | 3.83 | 2.50 | 2.89 | 1.34 | 3.60 | 1.35 |
| **O1_hsa-miR-874-3p** | PmiRNA | 6.27 | 1.26 | 6.11 | 1.07 | 6.46 | 1.68 | 6.16 | 0.85 | 5.86 | 1.15 |
| **O1_hsa-miR-877-5p** | PmiRNA | 7.39 | 1.54 | 7.35 | 1.73 | 7.33 | 2.50 | 7.14 | 1.69 | 8.10 | 2.70 |
| **O1_hsa-miR-885-5p** | PmiRNA | 6.34 | 2.10 | 6.23 | 2.40 | 6.36 | 2.64 | 6.37 | 1.84 | 5.60 | 1.61 |
| **O1_hsa-miR-92a-3p** | PmiRNA | -3.13 | 0.91 | -3.02 | 0.85 | -3.78 | 1.02 | -2.83 | 0.69 | -3.59 | 0.46 |
| **O1_hsa-miR-92b-3p** | PmiRNA | 7.35 | 1.30 | 7.60 | 1.08 | 7.21 | 2.01 | 7.32 | 1.04 | 7.46 | 1.58 |
| **O1_hsa-miR-93-3p** | PmiRNA | 4.13 | 0.62 | 4.31 | 0.81 | 4.31 | 1.82 | 3.97 | 0.61 | 4.10 | 1.13 |
| **O1_hsa-miR-93-5p** | PmiRNA | -2.08 | 0.42 | -2.04 | 0.33 | -2.11 | 0.43 | -1.94 | 0.27 | -2.17 | 0.27 |
| **O1_hsa-miR-99a-5p** | PmiRNA | 4.47 | 0.85 | 4.48 | 1.04 | 4.10 | 1.05 | 4.22 | 0.75 | 4.19 | 0.92 |
| **O1_hsa-miR-99b-5p** | PmiRNA | 3.33 | 1.09 | 3.26 | 0.86 | 3.15 | 1.26 | 2.93 | 0.84 | 3.44 | 1.16 |
| **O1_hsa-miR-378a-3p** | PmiRNA | 3.05 | 0.85 | 3.21 | 1.05 | 2.87 | 1.35 | 3.18 | 0.68 | 2.67 | 0.74 |
| **O2_3-O-methyldopa** | PMetas | 16.93 | 7.13 | 32.44 | 59.97 | 17.54 | 9.29 | 16.36 | 4.64 | 17.61 | 4.90 |
| **O2_Metanephrine** | PMetas | 24.86 | 11.86 | 447.19 | 721.07 | 22.67 | 9.41 | 33.59 | 14.34 | 31.57 | 10.71 |
| **O2_3-methoxytyramine** | PMetas | 5.44 | 3.24 | 91.92 | 444.10 | 4.74 | 2.58 | 8.88 | 6.29 | 5.27 | 2.30 |
| **O2_Normetanephrine** | PMetas | 74.94 | 36.50 | 1470.33 | 2682.42 | 72.70 | 36.45 | 105.91 | 59.03 | 58.35 | 27.41 |
| **O3_Aldosterone** | PSteroids | 0.25 | 0.30 | 0.08 | 0.07 | 0.07 | 0.06 | 0.08 | 0.06 | 0.07 | 0.05 |
| **O3_Andostenedione** | PSteroids | 0.80 | 0.37 | 0.78 | 0.42 | 1.03 | 0.79 | 0.73 | 0.41 | 1.07 | 0.46 |
| **O3_Corticosterone** | PSteroids | 3.86 | 4.45 | 3.34 | 2.88 | 4.00 | 2.50 | 2.54 | 3.05 | 4.07 | 4.20 |
| **O3_Cortisol** | PSteroids | 119.85 | 58.77 | 153.06 | 190.85 | 164.27 | 56.97 | 112.54 | 44.90 | 127.28 | 49.75 |
| **O3_Cortisone** | PSteroids | 15.90 | 6.22 | 20.17 | 5.64 | 19.76 | 5.28 | 17.06 | 5.53 | 22.87 | 5.30 |
| **O3_DHEA** | PSteroids | 3.67 | 2.53 | 2.67 | 1.95 | 2.39 | 2.80 | 3.45 | 2.51 | 6.26 | 3.76 |
| **O3_DHEAS** | PSteroids | 1236.66 | 678.30 | 1050.07 | 831.02 | 764.53 | 1320.37 | 1403.98 | 841.57 | 1924.93 | 853.96 |
| **O3_Pregnenolone** | PSteroids | 1.29 | 1.48 | 0.95 | 1.05 | 1.04 | 1.14 | 1.23 | 1.52 | 3.34 | 2.42 |
| **O3_Progesterone** | PSteroids | 0.68 | 2.04 | 1.12 | 4.69 | 1.49 | 6.60 | 0.20 | 1.17 | 1.25 | 3.43 |
| **O3_Testosterone** | PSteroids | 2.49 | 2.35 | 2.03 | 2.34 | 0.53 | 1.04 | 2.14 | 2.49 | 3.77 | 3.12 |
| **O3_11-deoxycorti**  **costerone** | PSteroids | 0.12 | 0.16 | 0.09 | 0.08 | 0.18 | 0.28 | 0.07 | 0.04 | 0.10 | 0.05 |
| **O3_11-deoxycortisol** | PSteroids | 0.53 | 0.54 | 0.46 | 0.47 | 2.96 | 9.19 | 0.27 | 0.27 | 0.29 | 0.25 |
| **O3_17OH-Progesterone** | PSteroids | 0.72 | 0.49 | 0.63 | 0.47 | 0.60 | 0.67 | 0.54 | 0.48 | 1.05 | 0.66 |
| **O3_18OH-Cortisol** | PSteroids | 0.97 | 0.89 | 0.58 | 0.37 | 0.85 | 0.70 | 0.68 | 0.61 | 0.73 | 0.34 |
| **O3_18oxo-Cortisol** | PSteroids | 0.07 | 0.12 | 0.02 | 0.02 | 0.01 | 0.02 | 0.02 | 0.07 | 0.01 | 0.01 |
| **O3_21-deoxycortisol** | PSteroids | 0.04 | 0.04 | 0.02 | 0.04 | 0.18 | 0.87 | 0.02 | 0.05 | 0.02 | 0.04 |
| **O4_acortol** | USteroids | 207.62 | 230.34 | 223.49 | 328.36 | 429.53 | 610.36 | 132.87 | 86.56 | 115.03 | 69.59 |
| **O4_acortolone** | USteroids | 2203.54 | 2442.60 | 2067.30 | 4628.41 | 2713.30 | 1705.99 | 1743.44 | 1073.00 | 1580.15 | 877.48 |
| **O4_An** | USteroids | 2421.84 | 2267.82 | 1312.41 | 1107.42 | 744.17 | 900.34 | 1965.46 | 1733.74 | 2949.18 | 2054.44 |
| **O4_bcortol** | USteroids | 601.50 | 537.49 | 589.94 | 563.36 | 972.83 | 969.63 | 417.97 | 304.27 | 478.43 | 345.07 |
| **O4_bcortolone** | USteroids | 1014.13 | 1106.44 | 1243.28 | 4307.39 | 1223.60 | 802.89 | 800.62 | 530.30 | 846.90 | 516.88 |
| **O4_Cortisol** | USteroids | 2003.00 | 8650.81 | 1754.00 | 13817.04 | 391.87 | 607.81 | 91.82 | 115.38 | 71.42 | 51.26 |
| **O4_Cortisone** | USteroids | 158.40 | 124.62 | 137.96 | 261.75 | 259.60 | 232.12 | 91.73 | 63.08 | 102.95 | 70.03 |
| **O4_DHEA** | USteroids | 168.97 | 436.15 | 88.16 | 170.75 | 73.57 | 141.27 | 138.50 | 218.36 | 263.09 | 315.35 |
| **O4_Etio** | USteroids | 1682.47 | 1811.00 | 977.01 | 718.05 | 1482.73 | 1597.82 | 1286.36 | 931.81 | 1867.53 | 1352.16 |
| **O4_PD** | USteroids | 1195.06 | 1631.97 | 703.75 | 1393.49 | 1685.80 | 3279.68 | 558.65 | 985.77 | 1084.93 | 1308.57 |
| **O4_PT** | USteroids | 847.98 | 725.37 | 576.29 | 441.28 | 625.43 | 721.17 | 557.13 | 405.94 | 926.76 | 629.05 |
| **O4_THAldo** | USteroids | 395.50 | 1400.80 | 152.15 | 752.69 | 40.44 | 37.75 | 75.30 | 118.97 | 50.78 | 30.85 |
| **O4_THAs** | USteroids | 95.86 | 113.40 | 55.55 | 49.96 | 56.93 | 81.89 | 63.82 | 80.08 | 69.63 | 47.12 |
| **O4_THB** | USteroids | 193.51 | 209.11 | 136.90 | 119.54 | 242.20 | 272.04 | 146.72 | 230.71 | 122.42 | 73.33 |
| **O4_THDOC** | USteroids | 77.18 | 197.85 | 29.07 | 42.55 | 61.77 | 93.57 | 20.50 | 25.22 | 33.28 | 51.72 |
| **O4_THE** | USteroids | 4473.59 | 4097.52 | 3504.65 | 4373.95 | 4300.70 | 2397.95 | 3368.18 | 2601.78 | 3371.01 | 2440.46 |
| **O4_THF** | USteroids | 2845.02 | 3777.88 | 4436.93 | 21770.38 | 3838.67 | 3088.65 | 1797.49 | 1206.25 | 1549.57 | 907.02 |
| **O4_THS** | USteroids | 189.75 | 249.62 | 105.59 | 96.38 | 1043.03 | 2305.81 | 82.20 | 76.99 | 61.70 | 43.46 |
| **O4_11bOHAn** | USteroids | 835.78 | 1015.14 | 765.32 | 1386.68 | 845.70 | 992.17 | 688.89 | 417.24 | 661.98 | 380.24 |
| **O4_11bOHEt** | USteroids | 472.43 | 708.98 | 545.01 | 613.28 | 1323.27 | 1338.97 | 492.62 | 619.59 | 378.16 | 353.90 |
| **O4_11OxoEt** | USteroids | 527.38 | 590.43 | 436.15 | 382.26 | 698.83 | 539.10 | 517.70 | 393.92 | 433.93 | 415.58 |
| **O4_17-HP** | USteroids | 310.39 | 339.71 | 189.57 | 196.06 | 199.97 | 251.93 | 180.56 | 192.86 | 316.14 | 288.92 |
| **O4_18-OHF** | USteroids | 236.89 | 278.24 | 85.98 | 85.04 | 131.28 | 133.40 | 110.29 | 126.47 | 101.08 | 76.98 |
| **O4_5aTHB** | USteroids | 219.82 | 250.50 | 147.04 | 114.94 | 142.13 | 197.86 | 147.14 | 146.53 | 172.48 | 106.47 |
| **O4_5aTHF** | USteroids | 2662.86 | 2994.98 | 2249.20 | 4186.10 | 2878.50 | 1758.15 | 2076.46 | 1394.89 | 1858.56 | 968.81 |
| **O4_5-PD** | USteroids | 80.05 | 74.17 | 53.26 | 47.36 | 79.60 | 93.12 | 70.95 | 57.67 | 120.61 | 87.37 |
| **O4_5-PT** | USteroids | 379.91 | 364.52 | 192.30 | 202.46 | 245.80 | 429.47 | 230.78 | 218.07 | 355.80 | 251.04 |
| **O5_AAA** | PSmallMB | 201.08 | 42.65 | 177.83 | 30.51 | 181.56 | 34.37 | 205.26 | 47.52 | 170.72 | 29.41 |
| **O5_Ac-Orn** | PSmallMB | 0.68 | 0.63 | 0.75 | 0.49 | 1.00 | 0.76 | 0.85 | 0.66 | 0.90 | 1.05 |
| **O5_ADMA / Arg** | PSmallMB | 0.01 | 0.01 | 0.01 | 0.00 | 0.01 | 0.01 | 0.01 | 0.01 | 0.01 | 0.00 |
| **O5_Ala** | PSmallMB | 374.03 | 127.71 | 344.65 | 69.86 | 427.76 | 113.14 | 386.51 | 116.84 | 319.30 | 85.89 |
| **O5_alpha-AAA** | PSmallMB | 0.66 | 0.28 | 0.58 | 0.21 | 0.62 | 0.25 | 0.60 | 0.24 | 0.46 | 0.19 |
| **O5_Arg** | PSmallMB | 74.40 | 30.55 | 71.72 | 24.23 | 78.52 | 34.23 | 76.49 | 31.80 | 76.91 | 18.07 |
| **O5_Asn** | PSmallMB | 46.69 | 13.75 | 43.97 | 12.29 | 45.49 | 10.01 | 45.41 | 14.48 | 36.23 | 10.79 |
| **O5_Asp** | PSmallMB | 9.14 | 3.48 | 8.43 | 3.09 | 10.10 | 5.62 | 14.66 | 10.38 | 11.92 | 12.11 |
| **O5_BCAA** | PSmallMB | 477.41 | 136.96 | 438.01 | 96.46 | 429.44 | 94.05 | 470.84 | 116.62 | 422.33 | 83.00 |
| **O5_C0** | PSmallMB | 34.79 | 10.17 | 37.13 | 8.96 | 36.03 | 9.87 | 39.46 | 13.61 | 33.63 | 8.69 |
| **O5_C10** | PSmallMB | 0.24 | 0.13 | 0.24 | 0.10 | 0.27 | 0.26 | 0.22 | 0.10 | 0.21 | 0.15 |
| **O5_C10:1** | PSmallMB | 0.13 | 0.05 | 0.12 | 0.04 | 0.14 | 0.07 | 0.13 | 0.05 | 0.11 | 0.04 |
| **O5_C10:2** | PSmallMB | 0.04 | 0.01 | 0.04 | 0.01 | 0.04 | 0.01 | 0.05 | 0.02 | 0.04 | 0.01 |
| **O5_C12** | PSmallMB | 0.10 | 0.04 | 0.10 | 0.03 | 0.10 | 0.06 | 0.11 | 0.04 | 0.10 | 0.05 |
| **O5_C12:1** | PSmallMB | 0.09 | 0.04 | 0.09 | 0.03 | 0.09 | 0.03 | 0.09 | 0.04 | 0.08 | 0.03 |
| **O5_C14** | PSmallMB | 0.04 | 0.02 | 0.04 | 0.01 | 0.04 | 0.01 | 0.04 | 0.02 | 0.03 | 0.01 |
| **O5_C14:1** | PSmallMB | 0.07 | 0.05 | 0.07 | 0.03 | 0.07 | 0.04 | 0.07 | 0.04 | 0.06 | 0.03 |
| **O5_C14:1-OH** | PSmallMB | 0.01 | 0.01 | 0.01 | 0.01 | 0.01 | 0.00 | 0.02 | 0.01 | 0.01 | 0.00 |
| **O5_C14:2** | PSmallMB | 0.02 | 0.02 | 0.02 | 0.01 | 0.02 | 0.02 | 0.02 | 0.01 | 0.02 | 0.01 |
| **O5_C16** | PSmallMB | 0.13 | 0.05 | 0.12 | 0.04 | 0.11 | 0.03 | 0.12 | 0.05 | 0.09 | 0.02 |
| **O5_C16:1** | PSmallMB | 0.04 | 0.01 | 0.04 | 0.01 | 0.04 | 0.01 | 0.04 | 0.02 | 0.04 | 0.01 |
| **O5_C16:1-OH** | PSmallMB | 0.01 | 0.01 | 0.01 | 0.00 | 0.01 | 0.00 | 0.01 | 0.00 | 0.01 | 0.00 |
| **O5_C18** | PSmallMB | 0.04 | 0.02 | 0.04 | 0.01 | 0.04 | 0.02 | 0.05 | 0.02 | 0.04 | 0.01 |
| **O5_C18:1** | PSmallMB | 0.16 | 0.09 | 0.14 | 0.06 | 0.14 | 0.05 | 0.15 | 0.09 | 0.10 | 0.03 |
| **O5_C18:2** | PSmallMB | 0.07 | 0.04 | 0.05 | 0.02 | 0.07 | 0.03 | 0.06 | 0.04 | 0.04 | 0.01 |
| **O5_C2** | PSmallMB | 7.29 | 3.33 | 9.12 | 6.45 | 7.14 | 2.01 | 7.03 | 2.98 | 6.53 | 2.46 |
| **O5_C2 / C0** | PSmallMB | 0.22 | 0.14 | 0.25 | 0.15 | 0.20 | 0.06 | 0.18 | 0.05 | 0.21 | 0.12 |
| **O5_C3** | PSmallMB | 0.47 | 0.35 | 0.41 | 0.16 | 0.44 | 0.14 | 0.45 | 0.23 | 0.35 | 0.13 |
| **O5_C3-DC (C4-OH)** | PSmallMB | 0.07 | 0.07 | 0.07 | 0.04 | 0.07 | 0.03 | 0.06 | 0.03 | 0.06 | 0.03 |
| **O5_C4** | PSmallMB | 0.22 | 0.12 | 0.22 | 0.19 | 0.26 | 0.17 | 0.21 | 0.11 | 0.18 | 0.12 |
| **O5_C4:1** | PSmallMB | 0.03 | 0.01 | 0.04 | 0.04 | 0.03 | 0.01 | 0.04 | 0.01 | 0.03 | 0.01 |
| **O5_C5** | PSmallMB | 0.15 | 0.06 | 0.13 | 0.05 | 0.13 | 0.04 | 0.14 | 0.05 | 0.12 | 0.04 |
| **O5_C8** | PSmallMB | 0.14 | 0.07 | 0.14 | 0.06 | 0.16 | 0.15 | 0.13 | 0.05 | 0.12 | 0.08 |
| **O5_C9** | PSmallMB | 0.03 | 0.01 | 0.03 | 0.01 | 0.03 | 0.01 | 0.05 | 0.03 | 0.04 | 0.02 |
| **O5_Cit** | PSmallMB | 33.90 | 11.38 | 30.36 | 9.65 | 32.75 | 9.84 | 38.68 | 15.65 | 28.44 | 7.69 |
| **O5_Cit / Arg** | PSmallMB | 0.55 | 0.34 | 0.47 | 0.21 | 0.50 | 0.26 | 0.64 | 0.51 | 0.38 | 0.10 |
| **O5_Cit / Orn** | PSmallMB | 0.48 | 0.18 | 0.49 | 0.20 | 0.40 | 0.16 | 0.52 | 0.17 | 0.58 | 0.15 |
| **O5_CPT-I ratio** | PSmallMB | 0.01 | 0.00 | 0.01 | 0.00 | 0.00 | 0.00 | 0.00 | 0.00 | 0.00 | 0.00 |
| **O5_Creatinine** | PSmallMB | 88.09 | 24.47 | 84.20 | 36.51 | 78.98 | 14.95 | 83.85 | 21.61 | 79.95 | 13.64 |
| **O5_Essential AA** | PSmallMB | 962.91 | 226.21 | 876.41 | 151.08 | 895.09 | 167.88 | 928.76 | 192.77 | 818.65 | 132.31 |
| **O5_Fisher ratio** | PSmallMB | 2.37 | 0.41 | 2.47 | 0.40 | 2.39 | 0.40 | 2.32 | 0.37 | 2.49 | 0.37 |
| **O5_Gln** | PSmallMB | 549.34 | 122.19 | 540.26 | 117.04 | 562.22 | 110.66 | 499.77 | 143.17 | 512.80 | 206.15 |
| **O5_Glu** | PSmallMB | 67.06 | 36.65 | 60.29 | 36.03 | 62.35 | 39.24 | 131.62 | 106.33 | 106.75 | 146.42 |
| **O5_Glucogenic AA** | PSmallMB | 719.06 | 193.57 | 698.47 | 115.77 | 780.96 | 177.40 | 741.33 | 188.01 | 647.12 | 117.63 |
| **O5_Gly** | PSmallMB | 234.49 | 83.88 | 246.31 | 67.85 | 246.28 | 91.27 | 253.50 | 93.14 | 227.40 | 57.15 |
| **O5_H1** | PSmallMB | 5323.93 | 1778.99 | 5726.64 | 1811.06 | 5124.87 | 2068.17 | 4758.67 | 1350.01 | 4545.55 | 517.14 |
| **O5_His** | PSmallMB | 81.42 | 18.64 | 73.36 | 13.56 | 78.02 | 11.93 | 84.20 | 18.81 | 74.56 | 10.64 |
| **O5_Ile** | PSmallMB | 83.94 | 29.15 | 80.00 | 22.13 | 77.29 | 19.65 | 83.74 | 26.41 | 74.71 | 18.46 |
| **O5_Kynurenine / Trp** | PSmallMB | 0.04 | 0.01 | 0.05 | 0.02 | 0.04 | 0.01 | 0.05 | 0.01 | 0.04 | 0.01 |
| **O5_Leu** | PSmallMB | 151.84 | 46.75 | 140.96 | 34.57 | 137.84 | 31.80 | 151.85 | 41.57 | 133.53 | 28.01 |
| **O5_Lys** | PSmallMB | 175.17 | 42.75 | 169.84 | 29.92 | 185.34 | 44.86 | 173.28 | 38.19 | 143.78 | 28.88 |
| **O5_lysoPC a C14:0** | PSmallMB | 4.93 | 0.60 | 4.79 | 0.49 | 5.31 | 0.72 | 5.21 | 0.58 | 4.88 | 0.52 |
| **O5_lysoPC a C16:0** | PSmallMB | 68.57 | 20.44 | 61.41 | 16.80 | 73.59 | 20.14 | 77.03 | 24.02 | 59.57 | 18.29 |
| **O5_lysoPC a C16:1** | PSmallMB | 2.35 | 0.93 | 2.27 | 0.95 | 2.78 | 1.03 | 2.78 | 1.09 | 1.90 | 0.54 |
| **O5_lysoPC a C17:0** | PSmallMB | 1.56 | 0.59 | 1.39 | 0.48 | 1.62 | 0.52 | 1.76 | 0.68 | 1.38 | 0.48 |
| **O5_lysoPC a C18:0** | PSmallMB | 19.51 | 5.80 | 16.50 | 4.42 | 18.62 | 4.58 | 22.51 | 8.25 | 17.50 | 5.74 |
| **O5_lysoPC a C18:1** | PSmallMB | 17.51 | 6.54 | 15.93 | 5.17 | 18.98 | 6.54 | 19.23 | 6.10 | 17.05 | 4.56 |
| **O5_lysoPC a C18:2** | PSmallMB | 23.41 | 9.76 | 19.22 | 8.55 | 25.84 | 10.45 | 27.31 | 10.87 | 26.39 | 9.58 |
| **O5_lysoPC a C20:3** | PSmallMB | 2.52 | 0.93 | 2.18 | 0.70 | 2.55 | 0.82 | 2.56 | 0.68 | 2.12 | 0.61 |
| **O5_lysoPC a C20:4** | PSmallMB | 6.97 | 2.82 | 6.55 | 2.45 | 7.21 | 2.20 | 6.24 | 1.60 | 5.68 | 1.33 |
| **O5_Met** | PSmallMB | 25.81 | 8.16 | 22.28 | 5.88 | 22.94 | 5.26 | 22.10 | 9.36 | 20.05 | 7.05 |
| **O5_Met-SO** | PSmallMB | 0.66 | 0.24 | 0.65 | 0.21 | 0.83 | 0.64 | 3.34 | 3.95 | 3.29 | 5.66 |
| **O5_Met-SO / Met** | PSmallMB | 0.03 | 0.01 | 0.03 | 0.01 | 0.04 | 0.03 | 0.22 | 0.34 | 0.49 | 1.97 |
| **O5_MUFA (PC)** | PSmallMB | 247.40 | 69.08 | 240.38 | 53.41 | 264.49 | 70.19 | 264.16 | 69.32 | 210.77 | 50.81 |
| **O5_MUFA (PC) /**  **SFA (PC)** | PSmallMB | 9.34 | 1.80 | 9.35 | 1.67 | 9.96 | 1.52 | 8.69 | 1.88 | 8.74 | 1.69 |
| **O5_Non essential**  **AA** | PSmallMB | 1929.89 | 410.88 | 1850.07 | 290.98 | 1974.03 | 294.88 | 1996.22 | 354.98 | 1753.59 | 236.72 |
| **O5_Orn** | PSmallMB | 78.74 | 32.48 | 70.05 | 27.82 | 90.25 | 32.05 | 81.86 | 35.53 | 51.07 | 12.78 |
| **O5_Orn / Arg** | PSmallMB | 1.41 | 1.38 | 1.16 | 0.80 | 1.48 | 1.02 | 1.50 | 1.67 | 0.69 | 0.19 |
| **O5_PC aa C28:1** | PSmallMB | 2.26 | 0.62 | 2.25 | 0.67 | 2.60 | 0.81 | 2.67 | 0.79 | 2.04 | 0.52 |
| **O5_PC aa C30:0** | PSmallMB | 3.73 | 1.41 | 3.33 | 1.00 | 3.94 | 1.47 | 4.17 | 1.26 | 3.26 | 1.27 |
| **O5_PC aa C32:0** | PSmallMB | 11.07 | 2.68 | 10.63 | 2.21 | 10.59 | 3.11 | 11.53 | 2.72 | 8.77 | 1.87 |
| **O5_PC aa C32:1** | PSmallMB | 12.03 | 6.17 | 13.46 | 6.07 | 17.07 | 10.84 | 15.25 | 7.56 | 10.50 | 4.82 |
| **O5_PC aa C32:3** | PSmallMB | 0.36 | 0.10 | 0.35 | 0.10 | 0.42 | 0.12 | 0.52 | 0.21 | 0.34 | 0.08 |
| **O5_PC aa C34:1** | PSmallMB | 176.13 | 50.88 | 170.73 | 39.42 | 185.53 | 48.88 | 175.40 | 48.26 | 138.95 | 31.20 |
| **O5_PC aa C34:2** | PSmallMB | 293.67 | 76.36 | 259.77 | 52.24 | 298.12 | 69.65 | 294.01 | 56.06 | 239.23 | 38.73 |
| **O5_PC aa C34:3** | PSmallMB | 11.37 | 3.91 | 12.23 | 4.88 | 15.38 | 6.45 | 15.84 | 5.42 | 12.30 | 3.60 |
| **O5_PC aa C34:4** | PSmallMB | 1.50 | 0.58 | 1.40 | 0.57 | 2.00 | 1.04 | 1.72 | 0.56 | 1.24 | 0.49 |
| **O5_PC aa C36:0** | PSmallMB | 2.65 | 0.88 | 2.55 | 0.81 | 2.63 | 0.69 | 3.85 | 2.05 | 3.33 | 2.92 |
| **O5_PC aa C36:1** | PSmallMB | 37.74 | 11.16 | 34.65 | 9.13 | 38.83 | 10.72 | 42.75 | 12.59 | 32.45 | 8.03 |
| **O5_PC aa C36:2** | PSmallMB | 168.37 | 41.90 | 149.34 | 34.03 | 171.11 | 45.46 | 184.67 | 42.48 | 146.54 | 28.78 |
| **O5_PC aa C36:3** | PSmallMB | 107.24 | 28.58 | 97.38 | 24.12 | 113.58 | 33.52 | 115.14 | 28.18 | 86.19 | 19.37 |
| **O5_PC aa C36:4** | PSmallMB | 161.22 | 54.95 | 154.98 | 35.50 | 160.53 | 58.31 | 142.18 | 51.80 | 111.30 | 30.00 |
| **O5_PC aa C36:5** | PSmallMB | 20.56 | 12.27 | 23.29 | 12.65 | 23.80 | 12.42 | 25.93 | 13.79 | 15.46 | 7.17 |
| **O5_PC aa C36:6** | PSmallMB | 0.78 | 0.36 | 0.82 | 0.40 | 1.04 | 0.46 | 1.13 | 0.55 | 0.64 | 0.24 |
| **O5_PC aa C38:0** | PSmallMB | 2.87 | 1.11 | 2.71 | 0.93 | 2.63 | 0.79 | 3.35 | 1.35 | 2.81 | 1.72 |
| **O5_PC aa C38:3** | PSmallMB | 41.52 | 12.48 | 37.56 | 9.74 | 38.60 | 12.44 | 41.59 | 12.69 | 29.14 | 8.57 |
| **O5_PC aa C38:4** | PSmallMB | 91.77 | 30.04 | 87.86 | 20.66 | 82.33 | 30.80 | 80.51 | 32.04 | 61.12 | 19.29 |
| **O5_PC aa C38:5** | PSmallMB | 43.35 | 14.25 | 46.35 | 12.23 | 42.98 | 14.23 | 45.38 | 15.04 | 32.49 | 9.77 |
| **O5_PC aa C38:6** | PSmallMB | 73.26 | 29.99 | 77.22 | 26.66 | 70.05 | 26.01 | 68.87 | 24.17 | 44.07 | 14.12 |
| **O5_PC aa C40:1** | PSmallMB | 0.43 | 0.11 | 0.39 | 0.09 | 0.38 | 0.11 | 0.65 | 0.41 | 0.56 | 0.50 |
| **O5_PC aa C40:2** | PSmallMB | 0.41 | 0.15 | 0.34 | 0.10 | 0.35 | 0.15 | 0.94 | 0.84 | 0.72 | 0.88 |
| **O5_PC aa C40:3** | PSmallMB | 0.59 | 0.18 | 0.57 | 0.15 | 0.52 | 0.15 | 1.10 | 0.78 | 0.81 | 0.73 |
| **O5_PC aa C40:4** | PSmallMB | 3.00 | 0.96 | 2.68 | 0.72 | 2.62 | 1.05 | 3.19 | 1.15 | 2.39 | 0.72 |
| **O5_PC aa C40:5** | PSmallMB | 8.57 | 3.09 | 8.84 | 2.49 | 7.60 | 2.81 | 8.74 | 3.18 | 6.42 | 2.19 |
| **O5_PC aa C40:6** | PSmallMB | 25.24 | 10.22 | 25.81 | 8.99 | 20.87 | 8.22 | 24.07 | 9.52 | 14.34 | 4.91 |
| **O5_PC aa C42:0** | PSmallMB | 0.57 | 0.18 | 0.53 | 0.19 | 0.52 | 0.17 | 0.66 | 0.28 | 0.56 | 0.29 |
| **O5_PC aa C42:1** | PSmallMB | 0.30 | 0.09 | 0.26 | 0.08 | 0.27 | 0.09 | 0.40 | 0.22 | 0.32 | 0.23 |
| **O5_PC aa C42:2** | PSmallMB | 0.29 | 0.10 | 0.25 | 0.07 | 0.29 | 0.09 | 0.51 | 0.34 | 0.38 | 0.32 |
| **O5_PC aa C42:4** | PSmallMB | 0.21 | 0.06 | 0.18 | 0.05 | 0.18 | 0.08 | 0.38 | 0.28 | 0.25 | 0.21 |
| **O5_PC aa C42:5** | PSmallMB | 0.34 | 0.11 | 0.36 | 0.10 | 0.29 | 0.10 | 0.48 | 0.24 | 0.34 | 0.15 |
| **O5_PC aa C42:6** | PSmallMB | 0.38 | 0.11 | 0.38 | 0.11 | 0.32 | 0.10 | 0.51 | 0.22 | 0.37 | 0.15 |
| **O5_PC ae C30:0** | PSmallMB | 0.35 | 0.11 | 0.33 | 0.09 | 0.33 | 0.08 | 0.41 | 0.13 | 0.39 | 0.15 |
| **O5_PC ae C30:2** | PSmallMB | 0.28 | 0.04 | 0.27 | 0.03 | 0.29 | 0.04 | 0.36 | 0.13 | 0.31 | 0.11 |
| **O5_PC ae C32:1** | PSmallMB | 2.21 | 0.66 | 2.02 | 0.51 | 2.04 | 0.41 | 2.18 | 0.50 | 1.85 | 0.38 |
| **O5_PC ae C32:2** | PSmallMB | 0.56 | 0.17 | 0.55 | 0.15 | 0.59 | 0.14 | 0.65 | 0.21 | 0.50 | 0.12 |
| **O5_PC ae C34:0** | PSmallMB | 1.21 | 0.38 | 1.15 | 0.28 | 1.15 | 0.31 | 1.32 | 0.40 | 1.02 | 0.32 |
| **O5_PC ae C34:1** | PSmallMB | 7.66 | 2.18 | 7.46 | 1.66 | 7.79 | 1.62 | 8.04 | 2.05 | 6.78 | 1.31 |
| **O5_PC ae C34:2** | PSmallMB | 7.95 | 2.48 | 7.26 | 2.30 | 8.72 | 1.97 | 8.51 | 2.12 | 7.31 | 1.81 |
| **O5_PC ae C34:3** | PSmallMB | 5.51 | 1.91 | 4.87 | 1.91 | 5.82 | 1.77 | 5.93 | 1.63 | 5.28 | 1.54 |
| **O5_PC ae C36:0** | PSmallMB | 0.80 | 0.21 | 0.81 | 0.17 | 0.77 | 0.20 | 0.96 | 0.31 | 0.91 | 0.57 |
| **O5_PC ae C36:1** | PSmallMB | 5.99 | 1.70 | 5.96 | 1.41 | 6.83 | 1.77 | 10.39 | 6.05 | 9.62 | 8.91 |
| **O5_PC ae C36:2** | PSmallMB | 10.30 | 2.87 | 9.78 | 2.88 | 11.47 | 2.61 | 12.74 | 4.04 | 11.06 | 3.79 |
| **O5_PC ae C36:3** | PSmallMB | 5.21 | 1.52 | 4.81 | 1.46 | 5.61 | 1.25 | 5.80 | 1.44 | 4.75 | 1.14 |
| **O5_PC ae C36:4** | PSmallMB | 13.67 | 3.82 | 12.70 | 3.16 | 13.23 | 3.59 | 12.18 | 3.34 | 10.52 | 3.33 |
| **O5_PC ae C36:5** | PSmallMB | 10.82 | 3.50 | 10.25 | 2.62 | 11.04 | 3.11 | 9.52 | 2.77 | 7.75 | 2.19 |
| **O5_PC ae C38:0** | PSmallMB | 1.85 | 0.69 | 1.97 | 0.80 | 2.02 | 0.75 | 2.55 | 1.06 | 1.56 | 0.51 |
| **O5_PC ae C38:1** | PSmallMB | 0.60 | 0.22 | 0.46 | 0.20 | 0.49 | 0.61 | 2.68 | 3.04 | 2.46 | 4.33 |
| **O5_PC ae C38:2** | PSmallMB | 1.76 | 0.46 | 1.57 | 0.41 | 1.89 | 0.78 | 4.32 | 3.51 | 4.09 | 5.21 |
| **O5_PC ae C38:3** | PSmallMB | 3.34 | 0.99 | 3.15 | 0.82 | 3.58 | 1.26 | 6.22 | 3.96 | 4.30 | 3.18 |
| **O5_PC ae C38:4** | PSmallMB | 10.75 | 3.08 | 9.98 | 2.22 | 10.05 | 2.31 | 10.32 | 2.92 | 8.32 | 1.80 |
| **O5_PC ae C38:5** | PSmallMB | 14.53 | 4.01 | 13.48 | 2.81 | 13.60 | 3.23 | 12.91 | 3.56 | 10.78 | 2.78 |
| **O5_PC ae C38:6** | PSmallMB | 6.11 | 2.07 | 6.02 | 1.68 | 6.20 | 1.79 | 5.87 | 1.78 | 4.23 | 1.18 |
| **O5_PC ae C40:1** | PSmallMB | 1.40 | 0.40 | 1.32 | 0.34 | 1.42 | 0.37 | 2.00 | 0.94 | 1.56 | 0.98 |
| **O5_PC ae C40:2** | PSmallMB | 1.55 | 0.46 | 1.63 | 0.45 | 1.59 | 0.47 | 2.42 | 1.28 | 1.80 | 1.09 |
| **O5_PC ae C40:3** | PSmallMB | 1.04 | 0.29 | 0.96 | 0.23 | 1.10 | 0.70 | 2.58 | 2.14 | 1.59 | 1.50 |
| **O5_PC ae C40:4** | PSmallMB | 2.19 | 0.59 | 2.02 | 0.45 | 2.13 | 0.55 | 3.20 | 1.63 | 2.26 | 0.92 |
| **O5_PC ae C40:5** | PSmallMB | 2.92 | 0.78 | 2.84 | 0.61 | 2.83 | 0.67 | 4.25 | 2.04 | 2.86 | 0.91 |
| **O5_PC ae C40:6** | PSmallMB | 4.01 | 1.37 | 3.97 | 1.25 | 3.68 | 1.12 | 4.00 | 1.26 | 2.82 | 0.69 |
| **O5_PC ae C42:0** | PSmallMB | 0.70 | 0.12 | 0.72 | 0.13 | 0.68 | 0.12 | 0.82 | 0.25 | 0.76 | 0.33 |
| **O5_PC ae C42:1** | PSmallMB | 0.42 | 0.10 | 0.37 | 0.08 | 0.38 | 0.11 | 0.73 | 0.53 | 0.60 | 0.58 |
| **O5_PC ae C42:2** | PSmallMB | 0.56 | 0.15 | 0.53 | 0.13 | 0.56 | 0.12 | 0.87 | 0.51 | 0.63 | 0.42 |
| **O5_PC ae C42:3** | PSmallMB | 0.78 | 0.21 | 0.72 | 0.20 | 0.75 | 0.19 | 1.13 | 0.58 | 0.83 | 0.47 |
| **O5_PC ae C42:4** | PSmallMB | 0.77 | 0.23 | 0.72 | 0.22 | 0.76 | 0.21 | 0.98 | 0.41 | 0.77 | 0.26 |
| **O5_PC ae C42:5** | PSmallMB | 2.13 | 0.49 | 2.06 | 0.51 | 2.02 | 0.38 | 2.43 | 0.72 | 1.99 | 0.38 |
| **O5_PC ae C44:3** | PSmallMB | 0.15 | 0.04 | 0.14 | 0.04 | 0.15 | 0.04 | 0.31 | 0.23 | 0.22 | 0.16 |
| **O5_PC ae C44:4** | PSmallMB | 0.39 | 0.11 | 0.40 | 0.12 | 0.39 | 0.09 | 0.44 | 0.14 | 0.39 | 0.10 |
| **O5_PC ae C44:5** | PSmallMB | 1.72 | 0.57 | 1.64 | 0.61 | 1.53 | 0.41 | 1.40 | 0.45 | 1.38 | 0.37 |
| **O5_PC ae C44:6** | PSmallMB | 1.22 | 0.40 | 1.11 | 0.39 | 1.10 | 0.34 | 1.05 | 0.30 | 0.96 | 0.24 |
| **O5_Phe** | PSmallMB | 65.83 | 13.46 | 58.94 | 10.95 | 64.17 | 11.71 | 66.50 | 15.88 | 54.16 | 8.60 |
| **O5_Pro** | PSmallMB | 208.31 | 68.53 | 167.55 | 42.07 | 169.52 | 53.92 | 210.26 | 61.98 | 181.66 | 59.08 |
| **O5_PUFA (PC)** | PSmallMB | 1166.28 | 266.78 | 1093.98 | 192.86 | 1169.37 | 287.11 | 1181.18 | 263.30 | 907.41 | 159.78 |
| **O5_PUFA (PC) /**  **MUFA (PC)** | PSmallMB | 4.90 | 0.80 | 4.70 | 0.70 | 4.58 | 0.77 | 4.66 | 0.84 | 4.51 | 0.89 |
| **O5_PUFA (PC) /**  **SFA (PC)** | PSmallMB | 44.60 | 6.53 | 42.87 | 5.92 | 44.55 | 6.20 | 39.48 | 7.84 | 39.43 | 10.88 |
| **O5_Putrescine** | PSmallMB | 0.12 | 0.04 | 0.14 | 0.11 | 0.13 | 0.06 | 0.11 | 0.04 | 0.11 | 0.04 |
| **O5_Putrescine / Orn** | PSmallMB | 0.00 | 0.00 | 0.00 | 0.00 | 0.00 | 0.00 | 0.00 | 0.00 | 0.00 | 0.00 |
| **O5_SDMA / Arg** | PSmallMB | 0.01 | 0.01 | 0.01 | 0.01 | 0.01 | 0.01 | 0.01 | 0.01 | 0.01 | 0.00 |
| **O5_Ser** | PSmallMB | 110.95 | 32.00 | 108.49 | 27.11 | 107.10 | 27.58 | 102.48 | 28.65 | 99.74 | 21.22 |
| **O5_SFA (PC)** | PSmallMB | 26.53 | 6.17 | 25.91 | 5.25 | 26.63 | 6.47 | 31.05 | 8.40 | 25.03 | 8.58 |
| **O5_SM C16:0** | PSmallMB | 86.78 | 20.52 | 82.44 | 14.91 | 85.59 | 13.66 | 88.30 | 22.69 | 69.06 | 11.03 |
| **O5_SM C16:1** | PSmallMB | 13.93 | 3.45 | 13.17 | 3.17 | 15.93 | 3.72 | 14.77 | 4.72 | 10.64 | 2.02 |
| **O5_SM C18:0** | PSmallMB | 18.07 | 5.18 | 19.50 | 4.65 | 19.15 | 4.39 | 18.43 | 5.31 | 13.97 | 3.35 |
| **O5_SM C18:1** | PSmallMB | 9.17 | 2.84 | 9.80 | 2.67 | 10.59 | 2.61 | 9.66 | 3.50 | 6.85 | 1.64 |
| **O5_SM C20:2** | PSmallMB | 0.41 | 0.13 | 0.42 | 0.12 | 0.46 | 0.11 | 0.60 | 0.33 | 0.31 | 0.07 |
| **O5_SM C24:0** | PSmallMB | 18.48 | 5.07 | 16.13 | 3.64 | 16.54 | 3.42 | 18.56 | 4.50 | 14.31 | 3.25 |
| **O5_SM C24:1** | PSmallMB | 47.28 | 11.27 | 49.12 | 10.40 | 45.20 | 8.27 | 46.85 | 12.07 | 37.31 | 6.69 |
| **O5_SM (OH) C14:1** | PSmallMB | 5.24 | 1.48 | 5.04 | 1.45 | 5.74 | 1.47 | 5.85 | 1.87 | 4.41 | 1.15 |
| **O5_SM (OH) C16:1** | PSmallMB | 2.77 | 0.83 | 2.84 | 0.78 | 2.86 | 0.72 | 2.93 | 0.93 | 2.23 | 0.56 |
| **O5_SM (OH) C22:1** | PSmallMB | 11.55 | 3.22 | 10.22 | 2.51 | 10.88 | 2.14 | 11.79 | 3.53 | 8.54 | 2.11 |
| **O5_SM (OH) C22:2** | PSmallMB | 9.26 | 2.47 | 9.29 | 2.42 | 10.17 | 2.20 | 10.07 | 3.39 | 7.61 | 1.85 |
| **O5_SM (OH) C24:1** | PSmallMB | 1.23 | 0.41 | 1.08 | 0.28 | 0.99 | 0.19 | 1.18 | 0.38 | 0.93 | 0.24 |
| **O5_Spermidine** | PSmallMB | 0.13 | 0.10 | 0.12 | 0.07 | 0.14 | 0.16 | 0.07 | 0.04 | 0.07 | 0.03 |
| **O5_Spermidine /**  **Putrescine** | PSmallMB | 1.14 | 0.82 | 1.07 | 0.73 | 1.14 | 1.01 | 0.64 | 0.31 | 0.68 | 0.29 |
| **O5_Spermine /**  **Spermidine** | PSmallMB | 0.29 | 0.16 | 0.27 | 0.12 | 0.32 | 0.27 | 0.33 | 0.14 | 0.25 | 0.14 |
| **O5_t4-OH-Pro** | PSmallMB | 11.00 | 4.35 | 10.38 | 4.08 | 12.57 | 7.94 | 11.49 | 7.72 | 11.15 | 6.08 |
| **O5_Taurine** | PSmallMB | 56.65 | 20.94 | 58.91 | 22.39 | 55.04 | 19.27 | 65.48 | 25.88 | 42.25 | 9.68 |
| **O5_Thr** | PSmallMB | 154.23 | 51.46 | 129.93 | 31.03 | 138.65 | 40.08 | 132.71 | 35.07 | 121.82 | 31.38 |
| **O5_Total AA** | PSmallMB | 2891.18 | 602.90 | 2724.63 | 374.52 | 2869.57 | 414.04 | 2924.46 | 510.63 | 2571.52 | 329.06 |
| **O5_Total AC / C0** | PSmallMB | 0.30 | 0.17 | 0.32 | 0.15 | 0.29 | 0.09 | 0.25 | 0.06 | 0.29 | 0.14 |
| **O5_Total AC-DC /**  **Total AC** | PSmallMB | 0.02 | 0.01 | 0.02 | 0.01 | 0.02 | 0.01 | 0.02 | 0.01 | 0.03 | 0.01 |
| **O5_Total AC-OH /**  **Total AC** | PSmallMB | 0.02 | 0.01 | 0.02 | 0.00 | 0.02 | 0.00 | 0.02 | 0.00 | 0.02 | 0.01 |
| **O5_total DMA** | PSmallMB | 1.06 | 0.34 | 1.04 | 0.53 | 0.94 | 0.28 | 0.98 | 0.28 | 0.84 | 0.20 |
| **O5_Total DMA / Arg** | PSmallMB | 0.02 | 0.02 | 0.02 | 0.01 | 0.02 | 0.01 | 0.02 | 0.01 | 0.01 | 0.00 |
| **O5_Total lysoPC** | PSmallMB | 150.39 | 41.26 | 133.09 | 34.83 | 159.20 | 41.17 | 168.42 | 47.81 | 140.50 | 37.69 |
| **O5_Total lysoPC /**  **Total PC** | PSmallMB | 0.11 | 0.02 | 0.10 | 0.02 | 0.11 | 0.03 | 0.12 | 0.03 | 0.13 | 0.04 |
| **O5_Total PC** | PSmallMB | 1432.96 | 326.59 | 1358.61 | 234.66 | 1461.04 | 351.94 | 1477.04 | 325.49 | 1143.11 | 193.63 |
| **O5_Total PC aa** | PSmallMB | 1300.30 | 301.34 | 1232.02 | 216.83 | 1326.21 | 335.98 | 1325.04 | 298.61 | 1016.69 | 185.62 |
| **O5_Total PC ae** | PSmallMB | 131.55 | 30.95 | 126.02 | 25.80 | 134.91 | 24.79 | 151.57 | 39.78 | 127.07 | 33.75 |
| **O5_Total (PC+SM)** | PSmallMB | 1657.61 | 368.43 | 1577.79 | 258.13 | 1685.76 | 377.74 | 1707.34 | 372.96 | 1320.76 | 212.42 |
| **O5_Total SM** | PSmallMB | 225.53 | 51.55 | 219.77 | 40.20 | 224.66 | 36.33 | 229.83 | 59.19 | 176.42 | 29.58 |
| **O5_Total SM-non OH** | PSmallMB | 195.72 | 45.00 | 191.22 | 34.62 | 193.99 | 31.24 | 197.91 | 50.30 | 152.69 | 24.82 |
| **O5_Total SM-OH** | PSmallMB | 30.13 | 7.86 | 28.46 | 6.81 | 30.57 | 6.08 | 31.75 | 9.60 | 23.62 | 5.49 |
| **O5_Total SM-OH /**  **Total SM-non OH** | PSmallMB | 0.16 | 0.02 | 0.15 | 0.02 | 0.16 | 0.02 | 0.16 | 0.02 | 0.15 | 0.02 |
| **O5_Total SM /**  **Total PC** | PSmallMB | 0.16 | 0.02 | 0.17 | 0.03 | 0.16 | 0.02 | 0.16 | 0.03 | 0.16 | 0.03 |
| **O5_Total SM /**  **Total (SM+PC)** | PSmallMB | 0.14 | 0.02 | 0.14 | 0.02 | 0.14 | 0.02 | 0.14 | 0.02 | 0.14 | 0.02 |
| **O5_Trp** | PSmallMB | 63.78 | 15.62 | 57.01 | 11.06 | 54.28 | 11.63 | 64.23 | 14.68 | 58.49 | 11.58 |
| **O5_Tyr** | PSmallMB | 71.72 | 19.40 | 62.08 | 13.99 | 63.11 | 14.40 | 74.55 | 20.66 | 58.11 | 13.80 |
| **O5_Tyr / Phe** | PSmallMB | 1.10 | 0.23 | 1.06 | 0.16 | 0.99 | 0.16 | 1.13 | 0.17 | 1.08 | 0.20 |
| **O5_Val** | PSmallMB | 242.10 | 64.68 | 217.09 | 43.84 | 214.50 | 46.54 | 234.93 | 53.41 | 214.29 | 40.73 |
| **O5_(C2 + C3):C0** | PSmallMB | 0.23 | 0.14 | 0.26 | 0.14 | 0.22 | 0.06 | 0.19 | 0.05 | 0.22 | 0.12 |

**Supplementary Table 3:** Summary of samples and omics availability.

|  |  | **Omics Type** | | | | | | | | | **Disease Type** | | | |  |  |  |
| --- | --- | --- | --- | --- | --- | --- | --- | --- | --- | --- | --- | --- | --- | --- | --- | --- | --- |
| **Serial number** | **Number of Omics Available (out of 5)** | **PmiRNA** | | **PMetas** | | **PSteroids** | **USteroids** | | **PSmallMB** | | **CS** | **PA** | **PPGL** | **PHT** | **Sub.Total** | **NV** | **Total** |
| 1 | 5 | ✔ | | ✔ | | ✔ | ✔ | | ✔ | | 30 | 100 | 69 | 108 | 307 | 101 | 408 |
| 2 | 4 |  | | ✔ | | ✔ | ✔ | | ✔ | | 6 | 1 | 2 | 1 | 10 |  | 9 |
| 3 | 4 | ✔ | | ✔ | | ✔ |  | | ✔ | | 1 | 1 | 3 | 2 | 7 | 3 | 10 |
| 4 | 4 | ✔ | | ✔ | |  | ✔ | | ✔ | |  |  | 1 | 2 | 3 |  | 3 |
| 5 | 4 | ✔ | |  | | ✔ | ✔ | | ✔ | | 1 | 2 |  |  | 3 | 28 | 31 |
| 6 | 4 |  | | ✔ | | ✔ | ✔ | | ✔ | |  |  | 2 |  | 2 |  | 2 |
| 7 | 3 |  | |  | | ✔ | ✔ | | ✔ | |  | 1 |  |  | 1 |  | 1 |
| 8 | 3 | ✔ | |  | | ✔ |  | | ✔ | |  |  |  |  | 0 | 1 | 1 |
| 9 | 3 | ✔ | |  | |  | ✔ | | ✔ | | 2 | 7 | 1 |  | 10 |  | 10 |
| 10 | 2 |  | |  | |  | ✔ | | ✔ | |  | 1 |  |  | 1 |  | 1 |
| 11 | 2 | ✔ | |  | |  |  | | ✔ | | 1 |  |  |  | 1 |  | 1 |
| 12 | 1 |  | | ✔ | |  |  | |  | |  |  | 10 |  | 10 |  | 10 |
|  |  |  |  | |  | | |  | |  |  |  |  | **Grand Total:** | | | **487** |

**Supplementary Table 4:** Details of randomly partitioned training and testing datasets with Cushing’s syndrome (CS), primary aldosteronism (PA), pheochromocytoma or paraganglioma (PPGL) and primary hypertension (PHT).

| **Data** | **Disease** | **Sex** | | **Age Distribution** | | **Total Count** |
| --- | --- | --- | --- | --- | --- | --- |
|  |  | **Male** | **Female** | **Patient age >= 50** | **Patient age < 50** |  |
| *Training (80%)* | CS | 2 | 22 | 13 | 11 | 24 |
|  | PA | 46 | 34 | 31 | 49 | 80 |
|  | PPGL | 24 | 31 | 35 | 20 | 55 |
|  | PHT | 38 | 49 | 56 | 31 | 87 |
| *Testing (20%)* | CS | 1 | 5 | 2 | 4 | 6 |
|  | PA | 9 | 11 | 10 | 10 | 20 |
|  | PPGL | 6 | 8 | 4 | 10 | 14 |
|  | PHT | 9 | 12 | 13 | 8 | 21 |
|  | **Total** | **135** | **172** | **164** | **143** | **307** |

**Supplementary Table 5:** Details of classifier hyperparameters and settings used during model development.

| **Classifier** | **Hyperparameter**^1^ | **Type** | **Value** |
| --- | --- | --- | --- |
| J48 | batchSize | integer | 100 |
| J48 | binarySplits | logical | False |
| J48 | collapseTree | logical | True |
| J48 | confidenceFactor | real | 0.25 |
| J48 | debug | logical | False |
| J48 | doNotCheckCapabilities | logical | False |
| J48 | doNotMakeSplitPointActualValue | logical | False |
| J48 | minNumObj | integer | 2 |
| J48 | numDecimalPlaces | integer | 2 |
| J48 | numFolds | integer | 3 |
| J48 | reduceErrorPruning | logical | False |
| J48 | saveInstanceData | logical | False |
| J48 | seed | integer | 1 |
| J48 | subtreeRaising | logical | True |
| J48 | unpruned | logical | False |
| J48 | useLaplace | logical | False |
| J48 | useMDLcorrection | logical | True |
| NB | batchSize | integer | 100 |
| NB | debug | logical | False |
| NB | displayModelInOldFormat | logical | False |
| NB | doNotCheckCapabilities | logical | False |
| NB | numDecimalPlaces | integer | 2 |
| NB | useKernelEstimator | logical | False |
| NB | useSupervisedDiscretization | logical | False |
| IBk | KNN | integer | 1 |
| IBk | batchSize | integer | 100 |
| IBk | crossValidate | logical | False |
| IBk | debug | logical | False |
| IBk | distanceWeighting | String | No distance weighting |
| IBk | doNotCheckCapabilities | logical | False |
| IBk | meanSquared | logical | False |
| IBk | nearestNeighbourSearchAlgorithm | String | Euclidean Distance |
| IBk | numDecimalPlaces | integer | 2 |
| IBk | windowSize | integer | 0 |
| LB | ZMax | real | 3.0 |
| LB | batchSize | integer | 100 |
| LB | classifier | string | DecisionStump |
| LB | debug | logical | False |
| LB | doNotCheckCapabilities | logical | False |
| LB | likelihoodThreshold | real | -1.7976931348623157E308 |
| LB | numDecimalPlaces | integer | 2 |
| LB | numIterations | integer | 10 |
| LB | numThreads | integer | 1 |
| LB | poolSize | integer | 1 |
| LB | seed | integer | 1 |
| LB | shrinkage | real | 1.0 |
| LB | useEstimatedPriors | logical | False |
| LB | useResampling | logical | False |
| LB | weightThreshold | integer | 100 |
| LMT | batchSize | integer | 100 |
| LMT | convertNominal | logical | False |
| LMT | debug | logical | False |
| LMT | doNotCheckCapabilities | logical | False |
| LMT | doNotMakeSplitPointActualValue | logical | False |
| LMT | errorOnProbabilities | logical | False |
| LMT | fastRegression | logical | True |
| LMT | minNumInstances | integer | 15 |
| LMT | numBoostingIterations | Integer | -1 |
| LMT | numDecimalPlaces | Integer | 2 |
| LMT | splitOnResiduals | logical | False |
| LMT | useAIC | logical | False |
| LMT | weightTrimBeta | real | 0.0 |
| SL | batchSize | integer | 100 |
| SL | debug | logical | False |
| SL | doNotCheckCapabilities | logical | False |
| SL | errorOnProbabilities | logical | False |
| SL | heuristicStop | integer | 50 |
| SL | maxBoostingIterations | integer | 500 |
| SL | numBoostingIterations | integer | 10 |
| SL | numDecimalPlaces | integer | 2 |
| SL | useAIC | logical | False |
| SL | useCrossValidation | logical | True |
| SL | weightTrimBeta | real | 0.0 |
| RF | bagSizePercent | integer | 100 |
| RF | batchSize | integer | 100 |
| RF | breakTiesRandomly | logical | False |
| RF | calcOutOfBag | logical | False |
| RF | computeAttributeImportance | logical | False |
| RF | debug | logical | False |
| RF | doNotCheckCapabilities | logical | False |
| RF | maxDepth | integer | 0 |
| RF | numDecimalPlaces | integer | 2 |
| RF | numExecutionSlots | integer | 1 |
| RF | numFeatures | integer | 0 |
| RF | numIterations | integer | 100 |
| RF | outputOutOfBagComplexityStatistics | logical | False |
| RF | printClassifers | logical | False |
| RF | seed | integer | 1 |
| RF | storeOutOfBagPredictions | logical | False |
| SMO | batchSize | integer | 100 |
| SMO | buildCalibrationModels | logical | False |
| SMO | c | real | 1.0 |
| SMO | calibrator | string | Logistic |
| SMO | checksTurnedOff | logical | False |
| SMO | debug | logical | False |
| SMO | doNotCheckCapabilities | logical | False |
| SMO | epsilon | real | 1.0E-12 |
| SMO | filterType | string | Normalize |
| SMO | kernel | string | PolyKernel |
| SMO | numDecimalPlaces | integer | 2 |
| SMO | numFolds | integer | -1 |
| SMO | randomSeed | integer | 1 |
| SMO | toleranceParameter | real | 0.001 |

**Supplementary Table 6:** Classification results for training and testing set using top performing classifiers trained with and without balanced data for ALL-ALL disease combination.

|  |  |  | *Testing on Training set* | | | | | | | | | *Testing on Testing set* | | | | | | | | |
| --- | --- | --- | --- | --- | --- | --- | --- | --- | --- | --- | --- | --- | --- | --- | --- | --- | --- | --- | --- | --- |
| **Datatype** | **Classifier** | **Training dataset balanced (using synthetic data)** | **Correctly**  **Classified** | **Incorrectly**  **Classified** | **Total**  **Instances** | **Balanced**  **Accuracy (%)** | **Sensitivity (%)** | **Specificity (%)** | **AUC** | **F1** | **Kappa** | **Correctly**  **Classified** | **Incorrectly**  **Classified** | **Total**  **Instances** | **Balanced**  **Accuracy (%)** | **Sensitivity (%)** | **Specificity (%)** | **AUC** | **F1** | **Kappa** |
| MOmics | LB | N | 245 | 1 | 246 | 99.8 | 99.7 | 99.8 | 1 | 1 | 0.99 | 52 | 9 | 61 | 88.6 | 82.6 | 94.6 | 0.95 | 0.85 | 0.79 |
|  | LB | Y | 320 | 1 | 321 | 99.8 | 99.7 | 99.9 | 1 | 1 | 1 | 49 | 12 | 61 | 85.1 | 77.1 | 93 | 0.92 | 0.78 | 0.72 |
|  | RF | N | 246 | 0 | 246 | 100 | 100 | 100 | 1 | 1 | 1 | 53 | 8 | 61 | 89.6 | 83.9 | 95.2 | 0.95 | 0.86 | 0.81 |
|  | RF | Y | 321 | 0 | 321 | 100 | 100 | 100 | 1 | 1 | 1 | 54 | 7 | 61 | 91.9 | 88 | 95.9 | 0.95 | 0.89 | 0.84 |
|  | SL | N | 229 | 17 | 246 | 94.8 | 92.1 | 97.5 | 0.99 | 0.93 | 0.9 | 50 | 11 | 61 | 86.4 | 79.6 | 93.3 | 0.95 | 0.82 | 0.74 |
|  | SL | Y | 307 | 14 | 321 | 97.1 | 95.7 | 98.5 | 1 | 0.96 | 0.94 | 50 | 11 | 61 | 86.5 | 79.6 | 93.4 | 0.96 | 0.81 | 0.74 |
| PmiRNA | LB | N | 214 | 32 | 246 | 89.5 | 83.9 | 95.1 | 0.98 | 0.86 | 0.81 | 36 | 25 | 61 | 69.3 | 53.3 | 85.4 | 0.8 | 0.55 | 0.42 |
|  | LB | Y | 277 | 44 | 321 | 90.8 | 86.2 | 95.4 | 0.97 | 0.86 | 0.82 | 34 | 27 | 61 | 69 | 53.6 | 84.3 | 0.8 | 0.56 | 0.37 |
|  | RF | N | 246 | 0 | 246 | 100 | 100 | 100 | 1 | 1 | 1 | 37 | 24 | 61 | 67.4 | 49.2 | 85.6 | 0.84 | 0 | 0.43 |
|  | RF | Y | 321 | 0 | 321 | 100 | 100 | 100 | 1 | 1 | 1 | 43 | 18 | 61 | 79.4 | 69.3 | 89.4 | 0.86 | 0.71 | 0.58 |
|  | SL | N | 183 | 63 | 246 | 80.3 | 69.7 | 90.8 | 0.91 | 0.71 | 0.63 | 36 | 25 | 61 | 68.9 | 52.6 | 85.2 | 0.79 | 0.54 | 0.41 |
|  | SL | Y | 263 | 58 | 321 | 88.1 | 82.2 | 94 | 0.94 | 0.82 | 0.76 | 39 | 22 | 61 | 73.9 | 60.3 | 87.4 | 0.82 | 0.61 | 0.49 |
| PMetas | LB | N | 193 | 53 | 246 | 80.1 | 68.2 | 91.9 | 0.92 | 0.68 | 0.69 | 44 | 17 | 61 | 75 | 60.5 | 89.5 | 0.8 | 0 | 0.59 |
|  | LB | Y | 252 | 69 | 321 | 85.7 | 78.4 | 92.9 | 0.93 | 0.78 | 0.71 | 39 | 22 | 61 | 74.6 | 60.8 | 88.4 | 0.82 | 0.61 | 0.51 |
|  | RF | N | 246 | 0 | 246 | 100 | 100 | 100 | 1 | 1 | 1 | 41 | 20 | 61 | 72.5 | 57.3 | 87.6 | 0.84 | 0 | 0.52 |
|  | RF | Y | 321 | 0 | 321 | 100 | 100 | 100 | 1 | 1 | 1 | 38 | 23 | 61 | 71.9 | 56.6 | 87.2 | 0.83 | 0.57 | 0.48 |
|  | SL | N | 166 | 80 | 246 | 73.4 | 59 | 87.9 | 0.86 | 0.59 | 0.53 | 43 | 18 | 61 | 74.6 | 59.9 | 89.2 | 0.79 | 0 | 0.57 |
|  | SL | Y | 208 | 113 | 321 | 76.3 | 64.3 | 88.3 | 0.86 | 0.64 | 0.53 | 34 | 27 | 61 | 67.2 | 48.8 | 85.7 | 0.77 | 0 | 0.4 |
| PSteroids | LB | N | 203 | 43 | 246 | 87.3 | 81.1 | 93.6 | 0.98 | 0.83 | 0.75 | 41 | 20 | 61 | 76.7 | 65.3 | 88 | 0.87 | 0.67 | 0.53 |
|  | LB | Y | 272 | 49 | 321 | 90 | 85.1 | 94.8 | 0.98 | 0.85 | 0.8 | 39 | 22 | 61 | 72.2 | 56.9 | 87.6 | 0.85 | 0.57 | 0.49 |
|  | RF | N | 246 | 0 | 246 | 100 | 100 | 100 | 1 | 1 | 1 | 39 | 22 | 61 | 74.7 | 62.9 | 86.6 | 0.86 | 0.66 | 0.48 |
|  | RF | Y | 321 | 0 | 321 | 100 | 100 | 100 | 1 | 1 | 1 | 43 | 18 | 61 | 80.7 | 71.8 | 89.6 | 0.88 | 0.71 | 0.59 |
|  | SL | N | 170 | 76 | 246 | 76.1 | 63.4 | 88.8 | 0.88 | 0.65 | 0.55 | 40 | 21 | 61 | 75.7 | 63.4 | 87.9 | 0.88 | 0.62 | 0.51 |
|  | SL | Y | 226 | 95 | 321 | 80.4 | 70.7 | 90.1 | 0.9 | 0.7 | 0.6 | 39 | 22 | 61 | 75.4 | 62.8 | 88 | 0.88 | 0.6 | 0.5 |
| USteroids | LB | N | 217 | 29 | 246 | 91.5 | 87.3 | 95.7 | 0.98 | 0.88 | 0.83 | 37 | 24 | 61 | 71.9 | 57.9 | 85.8 | 0.81 | 0.61 | 0.44 |
|  | LB | Y | 276 | 45 | 321 | 90.7 | 86.2 | 95.3 | 0.98 | 0.86 | 0.81 | 33 | 28 | 61 | 67.8 | 51.4 | 84.2 | 0.75 | 0.51 | 0.36 |
|  | RF | N | 246 | 0 | 246 | 100 | 100 | 100 | 1 | 1 | 1 | 41 | 20 | 61 | 75.1 | 62.1 | 88.1 | 0.84 | 0.65 | 0.53 |
|  | RF | Y | 321 | 0 | 321 | 100 | 100 | 100 | 1 | 1 | 1 | 33 | 28 | 61 | 67.5 | 51.4 | 83.5 | 0.79 | 0.53 | 0.34 |
|  | SL | N | 188 | 58 | 246 | 82.7 | 73.8 | 91.6 | 0.93 | 0.76 | 0.66 | 33 | 28 | 61 | 67.7 | 51.7 | 83.6 | 0.8 | 0.52 | 0.35 |
|  | SL | Y | 254 | 67 | 321 | 86.2 | 79.3 | 93 | 0.95 | 0.8 | 0.72 | 33 | 28 | 61 | 69.5 | 55.2 | 83.7 | 0.79 | 0.55 | 0.35 |
| PSmallMB | LB | N | 212 | 34 | 246 | 90 | 85 | 95 | 0.98 | 0.86 | 0.8 | 32 | 29 | 61 | 64.9 | 47.1 | 82.8 | 0.75 | 0.49 | 0.31 |
|  | LB | Y | 279 | 42 | 321 | 91.3 | 87 | 95.6 | 0.98 | 0.87 | 0.83 | 35 | 26 | 61 | 69.5 | 53.8 | 85.3 | 0.79 | 0.53 | 0.4 |
|  | RF | N | 246 | 0 | 246 | 100 | 100 | 100 | 1 | 1 | 1 | 31 | 30 | 61 | 62.9 | 43.5 | 82.3 | 0.78 | 0.44 | 0.29 |
|  | RF | Y | 321 | 0 | 321 | 100 | 100 | 100 | 1 | 1 | 1 | 31 | 30 | 61 | 64.6 | 46.4 | 82.7 | 0.76 | 0.47 | 0.3 |
|  | SL | N | 187 | 59 | 246 | 81.8 | 72.1 | 91.4 | 0.94 | 0.73 | 0.66 | 40 | 21 | 61 | 72.9 | 58.2 | 87.6 | 0.79 | 0.59 | 0.5 |
|  | SL | Y | 261 | 60 | 321 | 87.6 | 81.4 | 93.8 | 0.95 | 0.81 | 0.75 | 38 | 23 | 61 | 71 | 55.7 | 86.4 | 0.78 | 0.57 | 0.46 |

**Supplementary Table 7:** Classification results for training and testing set using top performing classifiers trained with and without balanced data for EHT-PHT disease combination.

|  |  |  | *Testing on Training set* | | | | | | | | | *Testing on Testing set* | | | | | | | | |
| --- | --- | --- | --- | --- | --- | --- | --- | --- | --- | --- | --- | --- | --- | --- | --- | --- | --- | --- | --- | --- |
| **Datatype** | **Classifier** | **Training dataset balanced (using downsampling)** | **Correctly**  **Classified** | **Incorrectly**  **Classified** | **Total**  **Instances** | **Balanced**  **Accuracy (%)** | **Sensitivity (%)** | **Specificity (%)** | **AUC** | **F1** | **Kappa** | **Correctly**  **Classified** | **Incorrectly**  **Classified** | **Total**  **Instances** | **Balanced**  **Accuracy (%)** | **Sensitivity (%)** | **Specificity (%)** | **AUC** | **F1** | **Kappa** |
| MOmics | LB | N | 235 | 11 | 246 | 94.5 | 98.1 | 90.8 | 0.99 | 0.97 | 0.9 | 48 | 13 | 61 | 74.7 | 87.5 | 61.9 | 0.93 | 0.84 | 0.51 |
|  | LB | Y | 168 | 6 | 174 | 96.6 | 95.4 | 97.7 | 1 | 0.97 | 0.93 | 50 | 11 | 61 | 80.6 | 85 | 76.2 | 0.85 | 0.86 | 0.61 |
|  | RF | N | 246 | 0 | 246 | 100 | 100 | 100 | 1 | 1 | 1 | 53 | 8 | 61 | 81 | 100 | 61.9 | 0.92 | 0.91 | 0.68 |
|  | RF | Y | 174 | 0 | 174 | 100 | 100 | 100 | 1 | 1 | 1 | 50 | 11 | 61 | 79.5 | 87.5 | 71.4 | 0.92 | 0.86 | 0.6 |
|  | SL | N | 229 | 17 | 246 | 92.1 | 95.6 | 88.5 | 0.98 | 0.95 | 0.85 | 51 | 10 | 61 | 80.7 | 90 | 71.4 | 0.95 | 0.88 | 0.63 |
|  | SL | Y | 165 | 9 | 174 | 94.8 | 94.3 | 95.4 | 0.99 | 0.95 | 0.9 | 54 | 7 | 61 | 87.9 | 90 | 85.7 | 0.96 | 0.91 | 0.75 |
| PmiRNA | LB | N | 228 | 18 | 246 | 91.5 | 95.6 | 87.4 | 0.97 | 0.94 | 0.84 | 48 | 13 | 61 | 74.7 | 87.5 | 61.9 | 0.8 | 0.84 | 0.51 |
|  | LB | Y | 164 | 10 | 174 | 94.3 | 93.1 | 95.4 | 0.98 | 0.94 | 0.89 | 40 | 21 | 61 | 65.8 | 65 | 66.7 | 0.79 | 0.71 | 0.29 |
|  | RF | N | 246 | 0 | 246 | 100 | 100 | 100 | 1 | 1 | 1 | 51 | 10 | 61 | 76.2 | 100 | 52.4 | 0.88 | 0.89 | 0.59 |
|  | RF | Y | 174 | 0 | 174 | 100 | 100 | 100 | 1 | 1 | 1 | 52 | 9 | 61 | 85.4 | 85 | 85.7 | 0.88 | 0.88 | 0.68 |
|  | SL | N | 216 | 30 | 246 | 86.1 | 91.8 | 80.5 | 0.95 | 0.91 | 0.73 | 52 | 9 | 61 | 82 | 92.5 | 71.4 | 0.83 | 0.89 | 0.66 |
|  | SL | Y | 161 | 13 | 174 | 92.5 | 93.1 | 92 | 0.97 | 0.93 | 0.85 | 49 | 12 | 61 | 81.6 | 77.5 | 85.7 | 0.85 | 0.84 | 0.59 |
| PMetas | LB | N | 195 | 51 | 246 | 73.8 | 92.5 | 55.2 | 0.88 | 0.85 | 0.51 | 45 | 16 | 61 | 67.6 | 87.5 | 47.6 | 0.81 | 0.81 | 0.38 |
|  | LB | Y | 143 | 31 | 174 | 82.2 | 78.2 | 86.2 | 0.92 | 0.81 | 0.64 | 43 | 18 | 61 | 70.7 | 70 | 71.4 | 0.77 | 0.76 | 0.39 |
|  | RF | N | 246 | 0 | 246 | 100 | 100 | 100 | 1 | 1 | 1 | 44 | 17 | 61 | 69.7 | 77.5 | 61.9 | 0.82 | 0.78 | 0.39 |
|  | RF | Y | 174 | 0 | 174 | 100 | 100 | 100 | 1 | 1 | 1 | 42 | 19 | 61 | 72.9 | 60 | 85.7 | 0.8 | 0.72 | 0.4 |
|  | SL | N | 162 | 84 | 246 | 56.1 | 89.3 | 23 | 0.69 | 0.77 | 0.14 | 38 | 23 | 61 | 50.9 | 87.5 | 14.3 | 0.7 | 0.75 | 0.02 |
|  | SL | Y | 119 | 55 | 174 | 68.4 | 60.9 | 75.9 | 0.8 | 0.66 | 0.37 | 38 | 23 | 61 | 64.5 | 57.5 | 71.4 | 0.69 | 0.67 | 0.26 |
| PSteroids | LB | N | 210 | 36 | 246 | 84.8 | 86.8 | 82.8 | 0.92 | 0.88 | 0.68 | 45 | 16 | 61 | 72.1 | 77.5 | 66.7 | 0.84 | 0.79 | 0.43 |
|  | LB | Y | 145 | 29 | 174 | 83.3 | 79.3 | 87.4 | 0.92 | 0.83 | 0.67 | 40 | 21 | 61 | 64.7 | 67.5 | 61.9 | 0.75 | 0.72 | 0.28 |
|  | RF | N | 246 | 0 | 246 | 100 | 100 | 100 | 1 | 1 | 1 | 44 | 17 | 61 | 66.3 | 85 | 47.6 | 0.81 | 0.8 | 0.35 |
|  | RF | Y | 174 | 0 | 174 | 100 | 100 | 100 | 1 | 1 | 1 | 44 | 17 | 61 | 69.7 | 77.5 | 61.9 | 0.8 | 0.78 | 0.39 |
|  | SL | N | 194 | 52 | 246 | 75.8 | 86.2 | 65.5 | 0.84 | 0.84 | 0.53 | 45 | 16 | 61 | 68.7 | 85 | 52.4 | 0.84 | 0.81 | 0.39 |
|  | SL | Y | 139 | 35 | 174 | 79.9 | 75.9 | 83.9 | 0.84 | 0.79 | 0.6 | 44 | 17 | 61 | 69.7 | 77.5 | 61.9 | 0.81 | 0.78 | 0.39 |
| USteroids | LB | N | 210 | 36 | 246 | 82.7 | 91.8 | 73.6 | 0.93 | 0.89 | 0.67 | 46 | 15 | 61 | 68.8 | 90 | 47.6 | 0.83 | 0.83 | 0.41 |
|  | LB | Y | 153 | 21 | 174 | 87.9 | 80.5 | 95.4 | 0.95 | 0.87 | 0.76 | 34 | 27 | 61 | 64 | 37.5 | 90.5 | 0.73 | 0.53 | 0.22 |
|  | RF | N | 246 | 0 | 246 | 100 | 100 | 100 | 1 | 1 | 1 | 52 | 9 | 61 | 83.1 | 90 | 76.2 | 0.87 | 0.89 | 0.67 |
|  | RF | Y | 174 | 0 | 174 | 100 | 100 | 100 | 1 | 1 | 1 | 43 | 18 | 61 | 73 | 65 | 81 | 0.83 | 0.74 | 0.41 |
|  | SL | N | 201 | 45 | 246 | 79.9 | 86.2 | 73.6 | 0.9 | 0.86 | 0.6 | 50 | 11 | 61 | 79.5 | 87.5 | 71.4 | 0.87 | 0.86 | 0.6 |
|  | SL | Y | 146 | 28 | 174 | 83.9 | 80.5 | 87.4 | 0.92 | 0.83 | 0.68 | 43 | 18 | 61 | 73 | 65 | 81 | 0.8 | 0.74 | 0.41 |
| PSmallMB | LB | N | 223 | 23 | 246 | 88.3 | 96.2 | 80.5 | 0.97 | 0.93 | 0.79 | 47 | 14 | 61 | 73.5 | 85 | 61.9 | 0.85 | 0.83 | 0.48 |
|  | LB | Y | 165 | 9 | 174 | 94.8 | 95.4 | 94.3 | 0.99 | 0.95 | 0.9 | 46 | 15 | 61 | 74.5 | 77.5 | 71.4 | 0.79 | 0.81 | 0.47 |
|  | RF | N | 246 | 0 | 246 | 100 | 100 | 100 | 1 | 1 | 1 | 51 | 10 | 61 | 78.5 | 95 | 61.9 | 0.82 | 0.88 | 0.61 |
|  | RF | Y | 174 | 0 | 174 | 100 | 100 | 100 | 1 | 1 | 1 | 48 | 13 | 61 | 75.8 | 85 | 66.7 | 0.83 | 0.84 | 0.52 |
|  | SL | N | 214 | 32 | 246 | 83.4 | 95.6 | 71.3 | 0.93 | 0.9 | 0.7 | 47 | 14 | 61 | 72.3 | 87.5 | 57.1 | 0.87 | 0.83 | 0.47 |
|  | SL | Y | 155 | 19 | 174 | 89.1 | 90.8 | 87.4 | 0.95 | 0.89 | 0.78 | 46 | 15 | 61 | 75.6 | 75 | 76.2 | 0.82 | 0.8 | 0.48 |

**Supplementary Table 8:** Classification results for training and testing set using top performing classifiers trained for PA-PHT disease combination. The training dataset was balanced, therefore no synthetic samples or down-sampling approach was used.

|  |  | *Testing on Training set* | | | | | | | | | *Testing on Testing set* | | | | | | | | |
| --- | --- | --- | --- | --- | --- | --- | --- | --- | --- | --- | --- | --- | --- | --- | --- | --- | --- | --- | --- |
| **Datatype** | **Classifier** | **Correctly**  **Classified** | **Incorrectly**  **Classified** | **Total**  **Instances** | **Balanced**  **Accuracy (%)** | **Sensitivity (%)** | **Specificity (%)** | **AUC** | **F1** | **Kappa** | **Correctly**  **Classified** | **Incorrectly**  **Classified** | **Total**  **Instances** | **Balanced**  **Accuracy (%)** | **Sensitivity (%)** | **Specificity (%)** | **AUC** | **F1** | **Kappa** |
| MOmics | LB | 164 | 3 | 167 | 98.2 | 97.5 | 98.9 | 1 | 0.98 | 0.96 | 35 | 6 | 41 | 85.2 | 80 | 90.5 | 0.88 | 0.84 | 0.71 |
|  | RF | 167 | 0 | 167 | 100 | 100 | 100 | 1 | 1 | 1 | 35 | 6 | 41 | 85.6 | 95 | 76.2 | 0.95 | 0.86 | 0.71 |
|  | SL | 158 | 9 | 167 | 94.6 | 93.8 | 95.4 | 0.99 | 0.94 | 0.89 | 37 | 4 | 41 | 90.4 | 95 | 85.7 | 0.95 | 0.9 | 0.81 |
| PmiRNA | LB | 155 | 12 | 167 | 92.9 | 95 | 90.8 | 0.97 | 0.93 | 0.86 | 35 | 6 | 41 | 85.6 | 95 | 76.2 | 0.91 | 0.86 | 0.71 |
|  | RF | 167 | 0 | 167 | 100 | 100 | 100 | 1 | 1 | 1 | 32 | 9 | 41 | 78.2 | 85 | 71.4 | 0.85 | 0.79 | 0.56 |
|  | SL | 152 | 15 | 167 | 90.9 | 88.7 | 93.1 | 0.96 | 0.9 | 0.82 | 33 | 8 | 41 | 80.7 | 90 | 71.4 | 0.86 | 0.82 | 0.61 |
| PMetas | LB | 136 | 31 | 167 | 81.6 | 86.3 | 77 | 0.88 | 0.82 | 0.63 | 32 | 9 | 41 | 78.1 | 80 | 76.2 | 0.78 | 0.78 | 0.56 |
|  | RF | 167 | 0 | 167 | 100 | 100 | 100 | 1 | 1 | 1 | 27 | 14 | 41 | 65.8 | 65 | 66.7 | 0.76 | 0.65 | 0.32 |
|  | SL | 119 | 48 | 167 | 71.4 | 73.8 | 69 | 0.77 | 0.71 | 0.43 | 31 | 10 | 41 | 75.8 | 85 | 66.7 | 0.82 | 0.77 | 0.51 |
| PSteroids | LB | 149 | 18 | 167 | 89 | 82.5 | 95.4 | 0.96 | 0.88 | 0.78 | 32 | 9 | 41 | 78 | 75 | 81 | 0.85 | 0.77 | 0.56 |
|  | RF | 167 | 0 | 167 | 100 | 100 | 100 | 1 | 1 | 1 | 32 | 9 | 41 | 78.1 | 80 | 76.2 | 0.9 | 0.78 | 0.56 |
|  | SL | 144 | 23 | 167 | 86 | 81.2 | 90.8 | 0.91 | 0.85 | 0.72 | 34 | 7 | 41 | 83 | 85 | 81 | 0.85 | 0.83 | 0.66 |
| USteroids | LB | 158 | 9 | 167 | 94.7 | 96.3 | 93.1 | 0.98 | 0.94 | 0.89 | 31 | 10 | 41 | 75.4 | 65 | 85.7 | 0.84 | 0.72 | 0.51 |
|  | RF | 167 | 0 | 167 | 100 | 100 | 100 | 1 | 1 | 1 | 33 | 8 | 41 | 80.2 | 70 | 90.5 | 0.88 | 0.78 | 0.61 |
|  | SL | 146 | 21 | 167 | 87.2 | 81.2 | 93.1 | 0.93 | 0.86 | 0.75 | 29 | 12 | 41 | 70.2 | 50 | 90.5 | 0.7 | 0.62 | 0.41 |
| PSmallMB | LB | 150 | 17 | 167 | 89.8 | 88.7 | 90.8 | 0.97 | 0.89 | 0.8 | 33 | 8 | 41 | 80.6 | 85 | 76.2 | 0.86 | 0.81 | 0.61 |
|  | RF | 167 | 0 | 167 | 100 | 100 | 100 | 1 | 1 | 1 | 31 | 10 | 41 | 75.8 | 85 | 66.7 | 0.83 | 0.77 | 0.51 |
|  | SL | 143 | 24 | 167 | 85.7 | 86.3 | 85.1 | 0.94 | 0.85 | 0.71 | 33 | 8 | 41 | 80.7 | 90 | 71.4 | 0.88 | 0.82 | 0.61 |

**Supplementary Table 9:** Classification results for training and testing set using top performing classifiers trained with and without balanced data for PPGL-PHT disease combination.

|  |  |  | *Testing on Training set* | | | | | | | | | *Testing on Testing set* | | | | | | | | |
| --- | --- | --- | --- | --- | --- | --- | --- | --- | --- | --- | --- | --- | --- | --- | --- | --- | --- | --- | --- | --- |
| **Datatype** | **Classifier** | **Training dataset balanced (using synthetic data)** | **Correctly**  **Classified** | **Incorrectly**  **Classified** | **Total**  **Instances** | **Balanced**  **Accuracy (%)** | **Sensitivity (%)** | **Specificity (%)** | **AUC** | **F1** | **Kappa** | **Correctly**  **Classified** | **Incorrectly**  **Classified** | **Total**  **Instances** | **Balanced**  **Accuracy (%)** | **Sensitivity (%)** | **Specificity (%)** | **AUC** | **F1** | **Kappa** |
| MOmics | LB | N | 142 | 0 | 142 | 100 | 100 | 100 | 1 | 1 | 1 | 33 | 2 | 35 | 92.9 | 85.7 | 100 | 0.95 | 0.92 | 0.88 |
|  | LB | Y | 168 | 1 | 169 | 99.4 | 98.8 | 100 | 1 | 0.99 | 0.99 | 34 | 1 | 35 | 96.4 | 92.9 | 100 | 0.99 | 0.96 | 0.94 |
|  | RF | N | 142 | 0 | 142 | 100 | 100 | 100 | 1 | 1 | 1 | 34 | 1 | 35 | 96.4 | 92.9 | 100 | 0.99 | 0.96 | 0.94 |
|  | RF | Y | 169 | 0 | 169 | 100 | 100 | 100 | 1 | 1 | 1 | 34 | 1 | 35 | 96.4 | 92.9 | 100 | 0.99 | 0.96 | 0.94 |
|  | SL | N | 137 | 5 | 142 | 95.5 | 90.9 | 100 | 1 | 0.95 | 0.92 | 32 | 3 | 35 | 89.3 | 78.6 | 100 | 0.95 | 0.88 | 0.81 |
|  | SL | Y | 169 | 0 | 169 | 100 | 100 | 100 | 1 | 1 | 1 | 32 | 3 | 35 | 90.5 | 85.7 | 95.2 | 0.95 | 0.89 | 0.82 |
| PmiRNA | LB | N | 135 | 7 | 142 | 94 | 89.1 | 98.9 | 0.98 | 0.93 | 0.89 | 28 | 7 | 35 | 81 | 85.7 | 76.2 | 0.9 | 0.77 | 0.6 |
|  | LB | Y | 158 | 11 | 169 | 93.5 | 92.7 | 94.3 | 0.96 | 0.93 | 0.87 | 31 | 4 | 35 | 90.5 | 100 | 81 | 0.99 | 0.88 | 0.77 |
|  | RF | N | 142 | 0 | 142 | 100 | 100 | 100 | 1 | 1 | 1 | 30 | 5 | 35 | 84.5 | 78.6 | 90.5 | 0.95 | 0.81 | 0.7 |
|  | RF | Y | 169 | 0 | 169 | 100 | 100 | 100 | 1 | 1 | 1 | 30 | 5 | 35 | 85.7 | 85.7 | 85.7 | 0.97 | 0.83 | 0.71 |
|  | SL | N | 114 | 28 | 142 | 78.2 | 69.1 | 87.4 | 0.88 | 0.73 | 0.58 | 26 | 9 | 35 | 73.8 | 71.4 | 76.2 | 0.9 | 0.69 | 0.47 |
|  | SL | Y | 143 | 26 | 169 | 84.5 | 79.3 | 89.7 | 0.93 | 0.83 | 0.69 | 28 | 7 | 35 | 81 | 85.7 | 76.2 | 0.93 | 0.77 | 0.6 |
| PMetas | LB | N | 140 | 2 | 142 | 98.2 | 96.4 | 100 | 1 | 0.98 | 0.97 | 33 | 2 | 35 | 92.9 | 85.7 | 100 | 0.97 | 0.92 | 0.88 |
|  | LB | Y | 164 | 5 | 169 | 97 | 96.3 | 97.7 | 1 | 0.97 | 0.94 | 33 | 2 | 35 | 94 | 92.9 | 95.2 | 0.97 | 0.93 | 0.88 |
|  | RF | N | 142 | 0 | 142 | 100 | 100 | 100 | 1 | 1 | 1 | 33 | 2 | 35 | 92.9 | 85.7 | 100 | 0.98 | 0.92 | 0.88 |
|  | RF | Y | 169 | 0 | 169 | 100 | 100 | 100 | 1 | 1 | 1 | 34 | 1 | 35 | 96.4 | 92.9 | 100 | 0.97 | 0.96 | 0.94 |
|  | SL | N | 133 | 9 | 142 | 92.2 | 85.5 | 98.9 | 0.96 | 0.91 | 0.86 | 32 | 3 | 35 | 90.5 | 85.7 | 95.2 | 0.99 | 0.89 | 0.82 |
|  | SL | Y | 158 | 11 | 169 | 93.4 | 91.5 | 95.4 | 0.97 | 0.93 | 0.87 | 32 | 3 | 35 | 91.7 | 92.9 | 90.5 | 0.99 | 0.9 | 0.82 |
| PSteroids | LB | N | 119 | 23 | 142 | 83.1 | 80 | 86.2 | 0.92 | 0.79 | 0.66 | 22 | 13 | 35 | 57.1 | 28.6 | 85.7 | 0.56 | 0.38 | 0.16 |
|  | LB | Y | 143 | 26 | 169 | 84.7 | 86.6 | 82.8 | 0.91 | 0.85 | 0.69 | 20 | 15 | 35 | 54.8 | 42.9 | 66.7 | 0.55 | 0.44 | 0.1 |
|  | RF | N | 142 | 0 | 142 | 100 | 100 | 100 | 1 | 1 | 1 | 23 | 12 | 35 | 60.7 | 35.7 | 85.7 | 0.67 | 0.45 | 0.23 |
|  | RF | Y | 169 | 0 | 169 | 100 | 100 | 100 | 1 | 1 | 1 | 22 | 13 | 35 | 59.5 | 42.9 | 76.2 | 0.65 | 0.48 | 0.2 |
|  | SL | N | 108 | 34 | 142 | 73.1 | 60 | 86.2 | 0.83 | 0.66 | 0.48 | 23 | 12 | 35 | 59.5 | 28.6 | 90.5 | 0.64 | 0.4 | 0.21 |
|  | SL | Y | 132 | 37 | 169 | 78.1 | 78 | 78.2 | 0.84 | 0.78 | 0.56 | 21 | 14 | 35 | 56 | 35.7 | 76.2 | 0.63 | 0.42 | 0.12 |
| USteroids | LB | N | 120 | 22 | 142 | 83.3 | 78.2 | 88.5 | 0.93 | 0.8 | 0.67 | 21 | 14 | 35 | 58.3 | 50 | 66.7 | 0.66 | 0.5 | 0.17 |
|  | LB | Y | 150 | 19 | 169 | 88.7 | 86.6 | 90.8 | 0.94 | 0.88 | 0.77 | 23 | 12 | 35 | 63.1 | 50 | 76.2 | 0.65 | 0.54 | 0.27 |
|  | RF | N | 142 | 0 | 142 | 100 | 100 | 100 | 1 | 1 | 1 | 27 | 8 | 35 | 75 | 64.3 | 85.7 | 0.8 | 0.69 | 0.51 |
|  | RF | Y | 169 | 0 | 169 | 100 | 100 | 100 | 1 | 1 | 1 | 23 | 12 | 35 | 63.1 | 50 | 76.2 | 0.73 | 0.54 | 0.27 |
|  | SL | N | 109 | 33 | 142 | 73 | 56.4 | 89.7 | 0.83 | 0.65 | 0.48 | 25 | 10 | 35 | 67.9 | 50 | 85.7 | 0.72 | 0.58 | 0.38 |
|  | SL | Y | 135 | 34 | 169 | 79.9 | 80.5 | 79.3 | 0.88 | 0.8 | 0.6 | 28 | 7 | 35 | 79.8 | 78.6 | 81 | 0.72 | 0.76 | 0.59 |
| PSmallMB | LB | N | 135 | 7 | 142 | 95 | 94.5 | 95.4 | 0.99 | 0.94 | 0.9 | 25 | 10 | 35 | 70.2 | 64.3 | 76.2 | 0.77 | 0.64 | 0.4 |
|  | LB | Y | 159 | 10 | 169 | 94 | 92.7 | 95.4 | 0.99 | 0.94 | 0.88 | 25 | 10 | 35 | 69 | 57.1 | 81 | 0.7 | 0.62 | 0.39 |
|  | RF | N | 142 | 0 | 142 | 100 | 100 | 100 | 1 | 1 | 1 | 25 | 10 | 35 | 71.4 | 71.4 | 71.4 | 0.82 | 0.67 | 0.42 |
|  | RF | Y | 169 | 0 | 169 | 100 | 100 | 100 | 1 | 1 | 1 | 25 | 10 | 35 | 72.6 | 78.6 | 66.7 | 0.81 | 0.69 | 0.43 |
|  | SL | N | 130 | 12 | 142 | 91.4 | 90.9 | 92 | 0.97 | 0.89 | 0.82 | 25 | 10 | 35 | 69 | 57.1 | 81 | 0.84 | 0.62 | 0.39 |
|  | SL | Y | 156 | 13 | 169 | 92.4 | 93.9 | 90.8 | 0.98 | 0.92 | 0.85 | 24 | 11 | 35 | 67.9 | 64.3 | 71.4 | 0.84 | 0.62 | 0.35 |

**Supplementary Table 10:** Classification results for training and testing set using top performing classifiers trained with and without balanced data for CS-PHT disease combination.

|  |  |  | *Testing on Training set* | | | | | | | | | *Testing on Testing set* | | | | | | | | |
| --- | --- | --- | --- | --- | --- | --- | --- | --- | --- | --- | --- | --- | --- | --- | --- | --- | --- | --- | --- | --- |
| **Datatype** | **Classifier** | **Training dataset balanced (using synthetic data)** | **Correctly**  **Classified** | **Incorrectly**  **Classified** | **Total**  **Instances** | **Balanced**  **Accuracy (%)** | **Sensitivity (%)** | **Specificity (%)** | **AUC** | **F1** | **Kappa** | **Correctly**  **Classified** | **Incorrectly**  **Classified** | **Total**  **Instances** | **Balanced**  **Accuracy (%)** | **Sensitivity (%)** | **Specificity (%)** | **AUC** | **F1** | **Kappa** |
| MOmics | LB | N | 111 | 0 | 111 | 100 | 100 | 100 | 1 | 1 | 1 | 25 | 2 | 27 | 83.3 | 66.7 | 100 | 1 | 0.8 | 0.76 |
|  | LB | Y | 159 | 0 | 159 | 100 | 100 | 100 | 1 | 1 | 1 | 25 | 2 | 27 | 83.3 | 66.7 | 100 | 0.95 | 0.8 | 0.76 |
|  | RF | N | 111 | 0 | 111 | 100 | 100 | 100 | 1 | 1 | 1 | 25 | 2 | 27 | 83.3 | 66.7 | 100 | 0.96 | 0.8 | 0.76 |
|  | RF | Y | 159 | 0 | 159 | 100 | 100 | 100 | 1 | 1 | 1 | 24 | 3 | 27 | 81 | 66.7 | 95.2 | 0.94 | 0.73 | 0.66 |
|  | SL | N | 111 | 0 | 111 | 100 | 100 | 100 | 1 | 1 | 1 | 26 | 1 | 27 | 91.7 | 83.3 | 100 | 0.93 | 0.91 | 0.89 |
|  | SL | Y | 159 | 0 | 159 | 100 | 100 | 100 | 1 | 1 | 1 | 25 | 2 | 27 | 83.3 | 66.7 | 100 | 0.87 | 0.8 | 0.76 |
| PmiRNA | LB | N | 111 | 0 | 111 | 100 | 100 | 100 | 1 | 1 | 1 | 22 | 5 | 27 | 70.2 | 50 | 90.5 | 0.84 | 0.55 | 0.43 |
|  | LB | Y | 159 | 0 | 159 | 100 | 100 | 100 | 1 | 1 | 1 | 21 | 6 | 27 | 73.8 | 66.7 | 81 | 0.9 | 0.57 | 0.43 |
|  | RF | N | 111 | 0 | 111 | 100 | 100 | 100 | 1 | 1 | 1 | 22 | 5 | 27 | 64.3 | 33.3 | 95.2 | 0.93 | 0.44 | 0.35 |
|  | RF | Y | 159 | 0 | 159 | 100 | 100 | 100 | 1 | 1 | 1 | 21 | 6 | 27 | 73.8 | 66.7 | 81 | 0.9 | 0.57 | 0.43 |
|  | SL | N | 104 | 7 | 111 | 86.9 | 75 | 98.9 | 0.99 | 0.84 | 0.8 | 25 | 2 | 27 | 83.3 | 66.7 | 100 | 0.93 | 0.8 | 0.76 |
|  | SL | Y | 157 | 2 | 159 | 98.9 | 100 | 97.7 | 1 | 0.99 | 0.97 | 25 | 2 | 27 | 89.3 | 83.3 | 95.2 | 0.95 | 0.83 | 0.79 |
| PMetas | LB | N | 97 | 14 | 111 | 75.4 | 54.2 | 96.6 | 0.92 | 0.65 | 0.58 | 22 | 5 | 27 | 70.2 | 50 | 90.5 | 0.67 | 0.55 | 0.43 |
|  | LB | Y | 137 | 22 | 159 | 86.6 | 91.7 | 81.6 | 0.92 | 0.86 | 0.72 | 16 | 11 | 27 | 44 | 16.7 | 71.4 | 0.58 | 0.15 | -0.11 |
|  | RF | N | 111 | 0 | 111 | 100 | 100 | 100 | 1 | 1 | 1 | 22 | 5 | 27 | 70.2 | 50 | 90.5 | 0.74 | 0.55 | 0.43 |
|  | RF | Y | 159 | 0 | 159 | 100 | 100 | 100 | 1 | 1 | 1 | 17 | 10 | 27 | 58.3 | 50 | 66.7 | 0.69 | 0.38 | 0.13 |
|  | SL | N | 93 | 18 | 111 | 68.5 | 41.7 | 95.4 | 0.85 | 0.53 | 0.44 | 21 | 6 | 27 | 67.9 | 50 | 85.7 | 0.71 | 0.5 | 0.36 |
|  | SL | Y | 116 | 43 | 159 | 72.7 | 69.4 | 75.9 | 0.86 | 0.7 | 0.45 | 17 | 10 | 27 | 58.3 | 50 | 66.7 | 0.72 | 0.38 | 0.13 |
| PSteroids | LB | N | 109 | 2 | 111 | 95.8 | 91.7 | 100 | 1 | 0.96 | 0.95 | 25 | 2 | 27 | 83.3 | 66.7 | 100 | 0.94 | 0.8 | 0.76 |
|  | LB | Y | 152 | 7 | 159 | 95.4 | 93.1 | 97.7 | 0.99 | 0.95 | 0.91 | 25 | 2 | 27 | 89.3 | 83.3 | 95.2 | 0.96 | 0.83 | 0.79 |
|  | RF | N | 111 | 0 | 111 | 100 | 100 | 100 | 1 | 1 | 1 | 25 | 2 | 27 | 83.3 | 66.7 | 100 | 0.96 | 0.8 | 0.76 |
|  | RF | Y | 159 | 0 | 159 | 100 | 100 | 100 | 1 | 1 | 1 | 25 | 2 | 27 | 83.3 | 66.7 | 100 | 0.98 | 0.8 | 0.76 |
|  | SL | N | 98 | 13 | 111 | 75.9 | 54.2 | 97.7 | 0.92 | 0.67 | 0.6 | 24 | 3 | 27 | 75 | 50 | 100 | 0.98 | 0.67 | 0.61 |
|  | SL | Y | 139 | 20 | 159 | 87.2 | 84.7 | 89.7 | 0.96 | 0.86 | 0.75 | 25 | 2 | 27 | 89.3 | 83.3 | 95.2 | 0.98 | 0.83 | 0.79 |
| USteroids | LB | N | 111 | 0 | 111 | 100 | 100 | 100 | 1 | 1 | 1 | 24 | 3 | 27 | 75 | 50 | 100 | 0.79 | 0.67 | 0.61 |
|  | LB | Y | 157 | 2 | 159 | 98.6 | 97.2 | 100 | 1 | 0.99 | 0.97 | 25 | 2 | 27 | 89.3 | 83.3 | 95.2 | 0.97 | 0.83 | 0.79 |
|  | RF | N | 111 | 0 | 111 | 100 | 100 | 100 | 1 | 1 | 1 | 24 | 3 | 27 | 75 | 50 | 100 | 0.95 | 0.67 | 0.61 |
|  | RF | Y | 159 | 0 | 159 | 100 | 100 | 100 | 1 | 1 | 1 | 25 | 2 | 27 | 83.3 | 66.7 | 100 | 0.94 | 0.8 | 0.76 |
|  | SL | N | 105 | 6 | 111 | 89 | 79.2 | 98.9 | 1 | 0.86 | 0.83 | 24 | 3 | 27 | 75 | 50 | 100 | 0.99 | 0.67 | 0.61 |
|  | SL | Y | 158 | 1 | 159 | 99.4 | 100 | 98.9 | 1 | 0.99 | 0.99 | 24 | 3 | 27 | 75 | 50 | 100 | 0.95 | 0.67 | 0.61 |
| PSmallMB | LB | N | 108 | 3 | 111 | 95.3 | 91.7 | 98.9 | 1 | 0.94 | 0.92 | 21 | 6 | 27 | 61.9 | 33.3 | 90.5 | 0.79 | 0.4 | 0.27 |
|  | LB | Y | 157 | 2 | 159 | 98.7 | 98.6 | 98.9 | 1 | 0.99 | 0.97 | 20 | 7 | 27 | 71.4 | 66.7 | 76.2 | 0.77 | 0.53 | 0.36 |
|  | RF | N | 111 | 0 | 111 | 100 | 100 | 100 | 1 | 1 | 1 | 22 | 5 | 27 | 70.2 | 50 | 90.5 | 0.79 | 0.55 | 0.43 |
|  | RF | Y | 159 | 0 | 159 | 100 | 100 | 100 | 1 | 1 | 1 | 22 | 5 | 27 | 70.2 | 50 | 90.5 | 0.79 | 0.55 | 0.43 |
|  | SL | N | 102 | 9 | 111 | 88.8 | 83.3 | 94.3 | 0.97 | 0.82 | 0.76 | 22 | 5 | 27 | 70.2 | 50 | 90.5 | 0.86 | 0.55 | 0.43 |
|  | SL | Y | 150 | 9 | 159 | 94.5 | 95.8 | 93.1 | 0.97 | 0.94 | 0.89 | 21 | 6 | 27 | 73.8 | 66.7 | 81 | 0.85 | 0.57 | 0.43 |

**Supplementary Note 1**: Description of Plasma miRNA extraction

Levels of 173 human feature miRNAs were measured in plasma. Total RNA was isolated from 200µL EDTA-plasma using the miRNeasy Mini kit (QIAGEN, Manchester, UK) standard protocol. Samples were eluted in 30µL RNase-free water. 4µL of undiluted RNA was then reverse-transcribed to cDNA in a 20µL reaction volume using the Universal cDNA synthesis kit II (Exiqon, Vedbaek, Denmark) standard protocol. For quality control purposes, plasma samples were spiked with UniSp2, UniSp4, and UniSp5 RNAs before RNA isolation and RNA samples were spiked with cel-miR-39-3p and UniSp6 cDNA during reverse transcription using components from the miRCURY LNA™ Universal microRNA PCR System RNA Spike-in kit (Exiqon). Selected plasma miRNAs were quantified using Serum/Plasma Focus microRNA PCR Panels (384-well, V4.M, Exiqon) according to their standard protocol, in combination with ExiLENT SYBR® Green master mix (Exiqon) and ROX solution (Thermo Fisher, Renfrew, UK) on a Quantstudio 12K Flex Real-time PCR System (Thermo Fisher). Raw data generated by the QuantStudio System were analysed using GenEx software (v.6, MultiD Analyses, Vedbaek, Denmark). UniSp3 enabled interpolate calibration. Quality control checks were performed using spike-in data; samples where RNA isolation spike-in assays deviated > +/- 2 Ct from the mean across all samples, or cDNA synthesis spike-in assays deviated > +/- 1 Ct from the mean, were flagged, as were samples where fewer than 90% of miRNAs amplified. Samples flagged in 2 or more categories were excluded from further analysis. MiRNAs with >50% missing data were excluded from further analysis as a feature. Data normalisation was performed to enable direct comparison of sample results, using the five miRNAs most stably-expressed across the dataset, as identified by Normfinder software;^2^ these were hsa-miR-106a-5p, hsa-miR-425-5p, hsa-miR-222-3p, hsa-let-7g-5p and hsa-let-7i-5p. Non-detected miRNA values were imputed by assigning them the value (*max* + 1), where *max* was the maximum Ct detected for that miRNA across all samples. Imputation accounted for 1.95% of data points across all subjects, including normotensive volunteers. Of the 179 unique human miRNAs measured on the PCR Panels, 5 were used for normalisation and 1 (hsa-miR-208a-3p) was excluded on quality grounds; a total of 173 plasma miRNAs were therefore analysed as potential diagnostic features (Supplementary Table 2).

**Supplementary Reference**

1 Witten IH, Frank E, Hall MA, Pal CJ. Data Mining: Practical Machine Learning Tools and Techniques, 4 edition. Amsterdam: Morgan Kaufmann, 2016.

2 Andersen CL, Jensen JL, Ørntoft TF. Normalization of real-time quantitative reverse transcription-PCR data: a model-based variance estimation approach to identify genes suited for normalization, applied to bladder and colon cancer data sets. *Cancer Res* 2004; **64**: 5245–50.
